# An ontological framework for organising and describing behaviours: The Human Behaviour Ontology

**DOI:** 10.12688/wellcomeopenres.21252.1

**Published:** 2024-05-08

**Authors:** Paulina M. Schenk, Robert West, Oscar Castro, Emily Hayes, Janna Hastings, Marie Johnston, Marta M. Marques, Elizabeth Corker, Alison J. Wright, Gabriella Stuart, Lisa Zhang, Micaela Santilli, Susan Michie

**Affiliations:** 1 Centre for Behaviour Change, University College London, London, England, UK; 2 Department of Behavioural Science and Health, University College London, London, England, UK; 3 Future Health Technologies, Singapore-ETH Centre, Campus for Research Excellence and Technological Enterprise (CREATE), Singapore, Singapore; 4 Institute for Implementation Science in Health Care, University of Zurich, Zurich, Switzerland; 5 School of Medicine, University of St. Gallen, St. Gallen, Switzerland; 6 Swiss Institute of Bioinformatics, Lausanne, Switzerland; 7 Aberdeen Health Psychology Group, University of Aberdeen, Aberdeen, Scotland, UK; 8 Comprehensive Health Research Centre, National School of Public Health, NOVA University of Lisbon, Lisbon, Portugal; 9 Clinical and Applied Psychology Unit, Department of Psychology, The University of Sheffield, Sheffield, England, UK; 10 Rotherham Doncaster and South Humber NHS Foundation Trust, Doncaster, UK; 11 Institute of Pharmaceutical Science, King's College London, London, England, UK

**Keywords:** behaviour, behavior, human, ontology, categorisation, classification, framework, machine learning, artificial intelligence

## Abstract

**Background:**

Human behaviours have been classified in areas such as health, occupation and sustainability. We aimed to develop a more broadly applicable framework for behaviours to facilitate integrating evidence across domains.

**Methods:**

The Human Behaviour Ontology (HBO), a part of the Behaviour Change Intervention Ontology (BCIO), was developed by: (1) specifying the ontology’s scope, (2) identifying candidate classes from existing classifications, (3) refining the ontology by applying it to code behaviours in relevant literature, (4) conducting a stakeholder review with behavioural and ontology experts, (5) testing the inter-rater reliability of its use in annotating research reports, (6) finalising classes and adding relations between classes, and (7) publishing the ontology’s computer-readable version.

**Results:**

A class labelled ‘individual human behaviour’ was defined as “ *A bodily process of a human that involves co-ordinated contraction of striated muscles controlled by the brain*.” In Steps 1-4, the ontology’s initial version was developed, with 128 classes. The inter-rater reliability for applying this version in annotations was 0.63 for researchers familiar with it and, after minor adjustments to the ontology and annotation guidance, 0.74 for researchers unfamiliar with it. Following Steps 5-6, the ontology was published with 177 classes, including 128 individual human behaviour classes organised under upper-level classes relating to (1) experiences (e.g., playing), (2) expressive (e.g., laughing), (3) harm (e.g., self-injury behaviour), (4) health (e.g., undergoing vaccination), (5) life-function (e.g., breathing behaviour), (6) interacting with materials (e.g., consumption), (7) bodily care (e.g., washing), (8) position (e.g., walking), and (9) social environments (e.g., communication). The remaining 49 classes included: ‘individual human behaviour pattern’ for repeated behaviours, ‘population behaviour’, ‘population behaviour pattern’, behavioural attributes (e.g., impulsiveness), and abstinence from behaviour. Relations were also defined to represent timings, locations, participants, mental processes, functions, goals, and outcomes.

**Conclusions:**

The HBO potentially provides a coherent framework for describing human behaviours.

## Plain Language summary

Human behaviours have been classified in many different ways. Having a unifying framework for describing behaviours could help us describe behaviours across different scientific domains more consistently and so better integrate the findings of different research studies. However, current classification systems for behaviour are limited in their scope, for example only focusing on behaviours that affect health. The current study aimed to develop a classification system called an ‘ontology’, to specify human behaviours extensively and coherently. Ontologies include classes (representations of anything that exists in the world, such as objects or processes) on a specific topic, with labels and definitions, and specified relations between classes. The Human Behaviour Ontology was developed by: (1) defining human behaviour and so specifying what the ontology should cover, (2) identifying classes for different types of human behaviours, (3) trying out the ontology’s classes by applying them to code examples of behaviours, and updating the ontology to amend any problematic classes, (4) seeking feedback on the ontology from behavioural science and ontology experts and refining it accordingly, (5) testing whether researchers can consistently apply the ontology, (6) finalising the classes of the ontology and specifying how they are related and (7) making the ontology computer-readable and available online. As a result, an ontology with 177 classes was developed, with 128 classes focused on ‘individual human behaviour’ and its subclasses (e.g., ‘personal bodily care behaviour’). Other classes included ones to represent repeated behaviours, behavioural attributes (e.g., whether a behaviour is done intentionally), behaviours done by groups of people as well as to describe when, where, with who/what, with what outcome and for what purpose behaviours might occur. This ontology could enable clearer and more consistent communication about behaviours and the study of behaviours, and facilitate more effective integration of research findings about human behaviour.

## Introduction

Human behaviour plays a central role in creating and solving problems for humankind ( Van Bavel *et al.*, 2020; Hulscher *et al.*, 2010; Klotz *et al.*, 2019; Marteau, 2017). Human wellbeing and life expectancy at the individual and population level is affected by behaviours, for example tobacco and alcohol use, physical exercise, dietary behaviours, harmful interpersonal behaviours and/or infection control behaviours ( Katzmarzyk & Lee, 2012; Mehta & Myrskylä, 2017). Therefore, the solution to many societal, health and environmental challenges lies in our ability to understand, predict, and ultimately shape human behaviour ( Ashiru-Oredope & Hopkins, 2015; Ghebreyesus, 2021; Gifford *et al.*, 2011). Considerable advances have been made in understanding the causal pathways (e.g., through motivation, environment, and abilities) that lead to specific behaviours (e.g., Bauman *et al.*, 2012; Michael *et al.*, 2014), as well as how our genetic make-up interacts with our experiences to generate behaviours (e.g., Maes *et al.*, 2004).

Many classification frameworks have been developed to describe, report and synthesise evidence about behaviours of a certain type or for a specific application (e.g., Ainsworth *et al.*, 2011; Fortune *et al.*, 2021; McEachan *et al.*, 2010). For instance, the International Classification of Health Interventions (ICHI; Fortune *et al.*, 2021) provides categories for behaviours relating to disability and health, such as ‘physical activity behaviours’ and ‘eating behaviours’. Researchers can apply this classification to consistently label and define behaviours within the specific health domain, as well as to extract and organise evidence about these behaviours. An important limitation of such frameworks is their scope. They do not attempt to represent behaviours beyond their specified domains or provide a means to describe detailed behavioural outcomes in research studies, e.g., abstinence from tobacco use for 6 months ( Baird *et al.*, 2023). Such details are important to guide clearer reporting of behaviours across studies, as well as to synthesise evidence and make systematic generalisations about behaviours.

A major challenge to creating a unifying framework is that the same behaviour can be classified in multiple ways depending on the purpose of the framework. For example, for robotics and animation purposes, it is necessary to describe behaviours in terms of physical movements and timing in order for these behaviour to be emulated accurately (e.g., Boulic *et al.*, 1990; Cully *et al.*, 2015; Magnenat-Thalmann & Thalmann, 2005). When attempting to understand and influence behaviours, such as waste recycling, tobacco use and shopping, there is a need to go beyond the purely physical description and characterise behaviours in terms of their functions, goals, outcomes, or interactions with other people or the physical environment (e.g., Chen *et al.*, 2021; Gkoutos *et al.*, 2012; Large, 2013; McEachan *et al.*, 2010). Walking, for example, may be described as a behaviour related to recreation, locomotion, protest (in the case of marches), or health promotion ( Aarts *et al.*, 1997; Büschges *et al.*, 2008; Yang & Diez-Roux, 2017). To complicate matters further, behaviours are also often classified in terms of their social meaning or external context ( Gkoutos *et al.*, 2012), for example, referring to ‘prosocial’ and ‘antisocial’ behaviours (e.g., Malti & Krettenauer, 2013). There is also divergence in what researchers mean by the term ‘behaviour’. Most definitions refer to potentially observable muscular actions, although some researchers include thoughts or secretory bodily responses ( Calhoun & El Hady, 2023; Davis *et al.*, 2015; Levitis *et al.*, 2009).

***Ontologies*** provide a potentially useful way of characterising behaviours ( Baird *et al.*, 2023; Michie *et al.*, 2017; Michie *et al.*, 2020). Ontologies usually represent domains of interest in terms of ***classes*** of ***entities*** (anything that exists in the universe, such as objects, ***processes*** and attributes) ( Arp *et al.*, 2015) (see glossary for bold italicised terms in Table 1) and the classes’ properties, which are specified as ***relations*** with other classes. In other fields, such as biomedicine, ontologies have successfully served as unifying categorisation frameworks to communicate about and synthesise knowledge ( Gene Ontology Consortium, 2019).

**Table 1.** Glossary of terms.

Term	Definition	Source
**Annotation**	Process of coding, or tagging, parts of documents or data sets to identify the presence of ontology classes or items of information.	Michie *et al.* (2017)
**Annotation guidance manual**	Written guidance on how to identify and tag pieces of text from intervention evaluation reports with specific codes relating to classes in the ontology, using for example EPPI-Reviewer software.	Michie *et al.* (2017)
**Basic Formal Ontology (BFO)**	An upper-level ontology specifying foundational distinctions between different types of entity, such as between continuants and occurrents, developed to support integration, especially of data obtained through scientific research.	Arp *et al.* (2015)
**Class**	Classes in ontologies represent types of entities in the world. The terms ‘entity’ and ‘class’ are often used interchangeably to refer to the entities represented in an ontology. Classes can be arranged hierarchically by the specification of parent and child classes (see definition of **parent class**)	Arp *et al.* (2015)
**Entity**	Anything that exists or can be imagined, including objects, processes, and their attributes. This includes mental process, i.e., the process and content of cognitive representations, and emotions.	Arp *et al.* (2015)
**EPPI-Reviewer**	A web-based software program for managing and analysing data in all types of systematic review (meta-analysis, framework synthesis, thematic synthesis etc. It manages references, stores PDF files and facilitates qualitative and quantitative analyses. It also has a facility to annotate published papers.	Thomas, Brunton & Graziosi (2010) EPPI-Reviewer 4: http://eppi.ioe.ac.uk/eppireviewer4/ EPPI-Reviewer Web Version: https://eppi.ioe.ac.uk/eppireviewer-web/
**GitHub**	A web-based platform used as a repository for sharing code, allowing version control.	https://github.com/
**Inter-rater reliability**	Statistical representation of degree of similarity and dissimilarity of coding between two or more coders. If inter-rater reliability is high this suggests that ontology class definitions and labels are being interpreted similarly by the coders.	Gwet (2014)
**Interoperability**	Two systems are interoperable to the extent that the information in one system can be used in the other system. An ontology is interoperable with another ontology if it can be used together with the other ontology.	http://www.obofoundry.org/principles/fp-010-collaboration.html
**Issue tracker**	An online log for problems identified by users accessing and using an ontology.	https://obofoundry.org/principles/fp-020-responsiveness.html BCIO Issue Tracker: https://github.com/HumanBehaviourChangeProject/ontologies/issues
**Logically defined class**	A class that is defined by logical expressions or axioms specifying the conditions under which something would be included in it. Axioms – logical operators such as conjunction (AND) and disjunction (OR) – can be used to write logical expressions to define a class. For instance, evaluative belief about a behaviour can be defined as “evaluative belief AND belief about behaviour”, meaning that this class captures only entities that fall in both classes – ‘evaluative belief’ and ‘belief about behaviour’	( Meehan *et al.*, 2011)
**Sibling class**	Two or more classes are sibling classes when they are direct subclasses of the same parent class.	( Noy & McGuinness, 2001)
**Open Biological and Biomedical Ontology (OBO) Foundry**	A collective of ontology developers that are committed to collaboration and adherence to shared principles. The mission of the OBO Foundry is to develop a family of interoperable ontologies that are both logically well-formed and scientifically accurate.	Smith *et al.* (2007) www.obofoundry.org/
**Ontology**	A standardised representational framework providing a set of classes for the consistent description (or ‘annotation’ or ‘tagging’) of data and information across disciplinary and research community boundaries.	Arp *et al.* (2015)
**Parent class**	A class within an ontology that is hierarchically related to one or more child classes (subclasses) such that all members of the child class are also members of the parent class, and all properties of the parent class are also properties of the child class.	Arp *et al.* (2015)
**Process**	Something that takes place over time.	Arp *et al.* (2015)
**Relation**	The manner in which two classes are connected or linked.	Arp *et al.* (2015)
**ROBOT**	An automated command line tool for ontology workflows.	Jackson *et al.* (2019) http://robot.obolibrary.org
**Uniform Resource Identifier (URI)**	A string of western characters that uniquely identifies a document or item of information. It is used in ontologies to identify individual classes and relations within the ontology. URIs are limited to the western alphabet; the extension of these identifiers including non-western alphabet are called Internationalised Resource Identifiers (IRI). All ontology entries should have URIs that form part of URLs.	http://www.obofoundry.org/principles/fp-003-uris.html
**Uniform Resource Locator (URL)**	A type of IRI that specifies a web address for a document or locatable resource on the internet. Ontology entries should all have individual URLs so that they can easily be referenced and located.	http://www.obofoundry.org/principles/fp-003-uris.html
**Versioning**	A process that involves keeping a record of different versions of files (e.g., about ontologies). Ontologies that have been released are expected to change over time as they are developed and refined, leading to a series of different files. Consumers of ontologies must be able to specify exactly which ontology files they used to encode their data or build their applications and be able to retrieve unaltered copies of those files in perpetuity. Versioning is one of the OBO Foundry principles.	http://www.obofoundry.org/principles/fp-004-versioning.html
**Web Ontology Language (OWL)**	A formal language for describing ontologies. It provides methods to model classes of ‘things’, how they relate to each other and the properties they have. OWL is designed to be interpreted by computer programs and is extensively used in the Semantic Web where rich knowledge about web documents and the relations between them are represented using OWL syntax.	https://www.w3.org/TR/owl2-quick-reference/

An important feature of ontologies is that every class and type of relation between classes is given a unique ID in the form of a ***Uniform Resource Identifier (URI)*** that facilitates searches for that class and its use in automated processing of information, such as for evidence synthesis or predicting intervention outcomes ( Hastings, 2017; Hastings *et al.*, 2023; Matentzoglu *et al.*, 2018; Michie *et al.*, 2017). In addition, ontologies that adopt community-agreed best-practices can support creation of more coherent and clear classes (e.g., behaviours) that promote interoperability across different research groups, data sets and scientific domains ( Gene Ontology Consortium, 2015; http://obofoundry.org/). One such best practice is that the definition of a class should take the form of its ***parent class*** (the class just above it in the semantic hierarchy) plus features that differentiate the class of interest from the parent class and other ***sibling classes*** that share the same parent class ( Arp *et al.*, 2015; Michie *et al.*, 2019; Seppälä *et al.*, 2017). For instance, the class with the label ‘sitting’ may have a parent class ‘posture behaviour’, and so the definition would be ‘A posture behaviour in which the person’s weight is supported by their buttocks.’ Another best practice example is that ontology developers should draw on the classes in existing ontologies where possible ( http://obofoundry.org/principles/fp-010-collaboration.html) and collaborate to improve their ontologies’ ***interoperability***. Ontologies should also be maintained and updated as required ( Arp *et al.*, 2015; He *et al.*, 2018).

**Figure 1.**
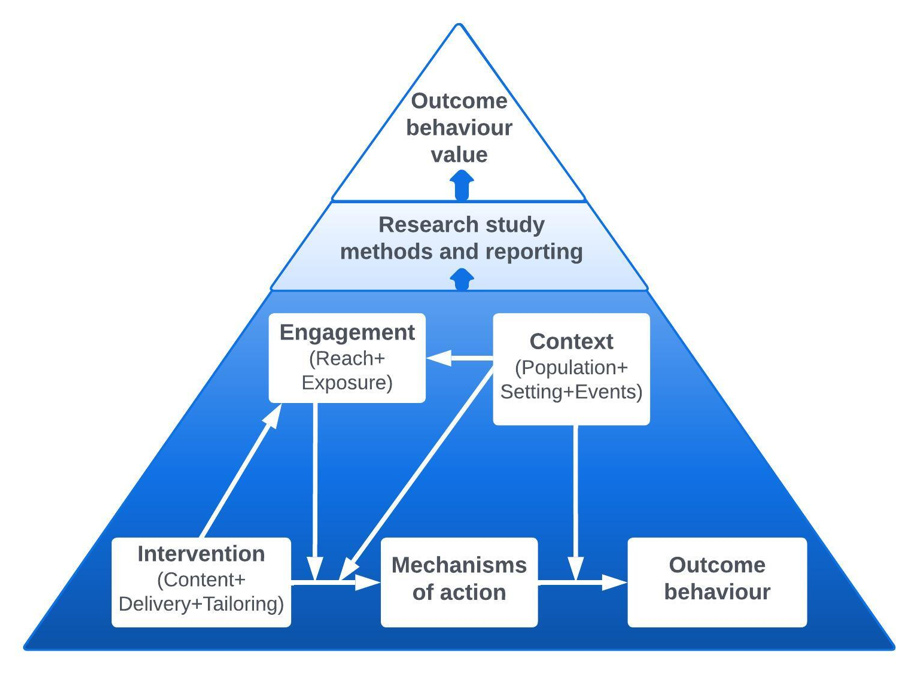
Schematic representation of the BCIO: key classes and causal connections. Source: Schenk *et al.*, 2023.

Some ontologies have been developed to capture behaviours in specific domains such as the Physical ACtivity Ontology (PACO) ( Braun *et al.*, 2023; Kim *et al.*, 2019). Other projects, such as the Tools for Understanding the Relation Between Behaviours using Ontologies (TURBBO) and the Human Behaviour-Change Project ( Michie *et al.*, 2017; Michie *et al.*, 2020), aim to represent behaviour more comprehensively. The TURBBO Project captures relations between a wide range of behaviours, for example mapping which behaviours (e.g., recycling and reducing food waste) are frequently correlated in studies ( https://sites.google.com/sheffield.ac.uk/turbbo/additional-resources#h.4k2unbgh9vh3). In parallel, the Human Behaviour-Change Project has developed the Behaviour Change Intervention Ontology (BCIO), which includes key classes about behaviour change interventions and their evaluations ( Michie *et al.*, 2017; Michie *et al.*, 2020; Wright *et al.*, 2020). One part of the BCIO is the Human Behaviour Ontology (HBO) described here, which aims to provide a framework for characterising behaviours extensively and in detail (see schematic representation of key classes in Figure 1).

## Aims

We aimed to develop an ontology (the Human Behaviour Ontology; HBO) that would (i) provide a systematic, extensive way of characterising human behaviours within behaviour change intervention scenarios, (ii) do so in a way that enabled computer readability, and (iii) be expandable as required to cover all behaviours of interest to ontology users.

## Methods

The HBO was developed using the established methods of the BCIO: an iterative process of seven steps ( Wright *et al.*, 2020). The steps were:

1.   Specifying the scope of the ontology

2.   Identifying candidate classes and developing preliminary definitions

3.   Refining the ontology by applying it to relevant literature 

4.   Expert stakeholder review

5.   Testing ***inter-rater reliability*** of researchers applying the ontology to annotate research reports

6.   Revising classes and adding relations between classes

7.   Making the ontology machine-readable and available online

The reporting of the ontology’s development follows the Minimum Information for Reporting an Ontology (MIRO) guidelines ( Matentzoglu *et al.*, 2018).

### Step 1: Specifying the scope of the HBO

The scope of the HBO was determined by discussion within the study team, given the aim of characterising the full set of behavioural variables used in research and as targets of behavioural interventions. 

### Step 2: Identifying candidate classes and developing their preliminary definitions

To produce a set of candidate classes and definitions, we started with a top-down approach. This included reviewing existing classification systems of human behaviour that were identified via (i) two widely used ontology repositories, Ontology Lookup Service ( www.ebi.ac.uk/ols4) and BioPortal ( https://bioportal.bioontology.org/), using the term ‘behaviour/behavior’, (ii) searches in Google Scholar using the terms ‘behaviour ontology’ and ‘behavior ontology’, and (iii) published classifications of human behaviour(s) of which the research team were aware. Table 2 shows the classification frameworks reviewed. We also searched for relevant categories in Roget’s Thesaurus ( Roget, 1997) and Medical Subject Headings (MeSH; https://meshb.nlm.nih.gov/search; Lipscomb, 2000) by using ‘behavior’ as the search term. We also hand-searched the content tables of the following academic journals: Annals of Behavioral Medicine and Nature Human Behaviour. The team then discussed what additional classes might be needed to capture behaviours more fully and to create a hierarchical classification of the behaviours, using ***Basic Formal Ontology (BFO)*** as the upper-level framework ( Arp *et al.*, 2015).

**Table 2.** Classification frameworks reviewed in developing the Human Behaviour Ontology.

Classification framework	Scope of behaviours in framework	Classification structure and/or application	Ontology *	Identified in Step 2 or 3 **
A fuzzy ontology for semantic modelling and recognition of human behaviour ( Díaz Rodríguez *et al.*, 2014)	Behaviours in general but with a focus on those that can be performed in offices	A formal application of fuzzy logic to ontological modelling of human activities	Yes	Step 2
A multi-domain ontology on healthy ageing for the characterization of older adults status and behaviour ( Mastropietro *et al.*, 2023)	Functionality and social behaviours in health ageing	An application of an ontology for physiological and behavioural characterisation of older adults	Yes	Step 2
Aging neuro-behavior ontology ( Martínez-Santiago *et al.*, 2020)	Behaviours relating to disability associated with ageing	Ontology describing the cognitive processes and behaviours involved in day-to-day living and whose performance usually decline with age	Yes	Step 2
An Automatic Ontology-Based Approach to Support Logical Representation of Observable and Measurable Data for Healthy Lifestyle Management: Proof-of-Concept Study ( Chatterjee *et al.*, 2021)	Behaviours that relate to sensor measurements (e.g., fitness trackers) in support of lifestyle management eHealth interventions	An ontology for behaviours, behavioural attributes, and physiological factors related to sensor measurements	Yes	Step 2
Characteristics of health-related behaviours ( McEachan *et al.*, 2010)	Behaviours relating to health and disease	Framework focused on identifying the characteristics of behaviour that are relevant to reliably discriminate health behaviours	No	Step 2
Common-sense taxonomy of health behaviours ( Nudelman & Shiloh, 2015)	Behaviours relating to health and disease	Taxonomy of health behaviours	No	Step 2
Compendium of Physical Activities ( Ainsworth *et al.*, 2011)	Behaviours relating to physical exercise	The Compendium provides a coding scheme linking categories and types of physical activity with their respective Metabolic Equivalent of Task (MET) intensity values	No	Step 2
Development and Validation of a Functional Behavioural Assessment Ontology to Support Behavioural Health Interventions ( Merlo *et al.*, 2018)	Behaviours that are relevant for the assessment of and management of problem behaviours	An ontology for variables and behaviours relating to functional behavioural assessment	Yes	Step 2
The International Classification of Health Interventions (ICHI) ( Fortune *et al.*, 2021)	Behaviours relating to disability, health and disease	The International Classification of Health Interventions (ICHI) is part of the WHO Family of International Classifications (e.g., ICF, ICD) and aims to provide a common language and structure for describing and capturing information about health promotion interventions	No	Step 2
NCI Thesaurus OBO Edition ( Balhoff *et al.*, 2017)	Behaviours relating to cancer	Reference terminology that includes broad coverage of the cancer domain	No	Step 2
PCLiON: An Ontology for Data Standardization and Sharing of Prostate Cancer Associated Lifestyles ( Chen *et al.*, 2021)	Behaviours relating to lifestyle risk factors in prostate cancer	Ontology describing specific behaviours of relevance to cancer	Yes	Step 2
Seven characteristics of living things ( Large, 2013)	Behaviours relating to survival and reproduction	High level classification of behaviour according to function	No	Step 2
The ‘Neurobehaviour ontology’ ( Gkoutos *et al.*, 2012)	Behaviours that are shared with animals (e.g., sexual behaviours, aggression, fear-related behaviours)	An ontology of behaviours developed within the biomedical domain for annotating the behaviour of animals. Includes several behaviours of relevance for humans too	Yes	Step 2
Addiction Ontology (AddictO) ( Hastings *et al.*, 2020)	Behaviours relating to addiction and its management or prevention	Ontology representing all of the constructs that researchers, practitioners and policy makers want to refer to in the field of addiction.	Yes	Step 3
ICD-11 ( Almeida *et al.*, 2020)	Behaviours relating to disability, health and disease	The International Classification of Diseases and Related Health Problems (ICD) aims to provide a global standard for coding health information and causes of death	No	Step 3
ICF-Behave V.1.0 ( Larsen *et al.*, 2021)	Behaviours relating to disability, health and disease	Taxonomy of behaviours in the context of the WHO international classification of functioning disability and health (ICF)	No	Step 3
Physical ACtivity Ontology (PACO) ( Kim *et al.*, 2019)	Behaviours relating to physical exercise	Ontology developed to support structuring and standardising heterogeneous descriptions of physical activities	Yes	Step 3
Townsville Residential Energy Demand (TRED) program behaviours	Behaviours relating to sustainability	List of behaviours that lead to reduction in home energy consumption identified through literature search and expert panel	No	Step 3

*A classification framework was considered to be an ontology when their authors referred to it as such and the classification system was available in Web Ontology Language (OWL). **Two searches were run to identify classification frameworks, one in Step 2 and the other in Step 3

Ontological definitions for the classes were drafted based on dictionaries, existing ontological definitions and group discussions informed by guidelines for ontological definitions ( Michie *et al.*, 2019; Seppälä *et al.*, 2017). In terms of the mechanics of ontology development, classes were originally set out in an Excel spreadsheet, with each class organised in a separate row with columns for its label, definition, parent class, informal definition, examples and relations.

### Step 3: Refining the ontology by applying it to relevant literature

We investigated whether the ontology structure was stable and comprehensive enough to allow characterisation of a wide range of behaviours in a coherent way through an iterative process of literature ***annotation***, team discussion, and revision. We identified examples of behaviours from three sources and assessed whether they could be annotated within the existing HBO structure. The sources were (i) five additional classification frameworks that were identified in an updated search, using the same method described in Step 2 (see Table 2), (ii) published systematic reviews of behaviour change interventions, and (iii) abstracts of studies published in journals addressing a breadth of human behaviours.

To identify the systematic reviews, a search using the terms ‘systematic review’ and ‘behaviour/behavior’ at the title level was performed in Web of Science, the Cochrane Library and ProQuest (February 2020). Fifteen reviews were included, covering a range of domains, such as environment, sexual health, education, and clinical practice. To identify abstracts, a search using the term ‘behavio*’ was performed in SCImago (a database of scientific journals) to generate a list of journals (September 2020). The 224 journals identified were divided into ‘broad’ (e.g., Nature Human Behaviour) or ‘specific’ (e.g., Journal of Consumer Behaviour). Five ‘broad’ journals were randomly selected and 20 abstracts from their last published issues (including ‘in press’ or ‘online first’) were retrieved for analysis (i.e., 100 abstracts in total). A list of these systematic reviews and abstracts can be found in: https://osf.io/hsvp4.

To test whether a behaviour found in a source could be clearly captured by classes in the ontology, two researchers (EH & OC) independently annotated mentions of behaviours (referred to as ‘example behaviours’) from these three sources, using the HBO prototype developed in Step 2. For example, from one abstract about political behaviour, the term ‘voting’ was annotated as belonging to the class ‘political behaviour’ in the HBO. When a source included synonyms for a behaviour (e.g., ‘taking medication’ and ‘taking prescribed drug’), this behaviour was annotated only once. During the annotation process, the researchers considered whether:

- Any class definitions needed to be reworded,
- The HBO structure needed to be altered,
- Any behaviours could not be captured by classes in the ontology, indicating the need for new classes.

Discrepancies in annotations were resolved by discussion between the two annotators or, if required, the whole research team. To avoid overpopulating the ontology with too many classes before having a stable structure, new classes were only added if they captured several of the extracted behaviours. For example, specific behaviours, such as toothbrushing and hair brushing, could be captured under a broader class of behaviours to take care of one’s body. These specific classes of behaviour were then included as examples in the higher-level classes to which they belonged. This was done under the principle that such examples could be added as classes in their own right once the structure of the ontology was clearly established and if users identified a need for them.

### Step 4: Expert stakeholder review

We invited 94 behaviour change experts and four ontology experts (see Michie *et al.*, 2020; Norris *et al.*, 2021) to review the ontology. Behaviour change experts were invited from a list of those who: (i) provided feedback on previous projects at the UCL Centre for Behaviour Change and indicated willingness to be contacted for future projects, or (ii) expressed interest in being involved in the Human Behaviour-Change Project stakeholder initiatives. To be eligible, these experts needed to have a doctoral degree in behavioural science or a related discipline. Experts from both 'well-represented' countries (UK, USA, Canada, Australia, the Netherlands) and 'less-represented' countries (e.g., Chile and France) were randomly selected to provide feedback. Ontology experts were suggested by JH, the Human Behaviour-Change Project’s ontology expert. Recruitment continued until at least 10 participants completed the stakeholder review; participation in the study was completely voluntary and not remunerated. For this stakeholder review, ethical approval was granted by University College London’s Research Ethics Committee (CEHP/2020/579) in February 2020. Participants provided informed written consent via an online Qualtrics survey before starting the review.

To support participants familiarising themselves with the HBO and the review task, the experts watched three introductory videos explaining what ontologies are, with a focus on the HBO ( https://vimeo.com/721051844; https://vimeo.com/726324041; https://vimeo.com/726779845). Feedback on the HBO was collected through an online questionnaire, using Qualtrics ^TM^ software ( https://www.qualtrics.com; see complete survey here: https://osf.io/dcmq4). Participants were presented with class labels, definitions, parent classes and, where relevant, their informal definitions and examples. Participants were asked to provide feedback through open-ended questions on:

- Clarity: whether the labels and definitions could be understood by experts who did not develop the HBO,
- Representativeness: whether the ontology comprehensively covered constructs of interest, i.e., whether any classes were missing, and
- Structure: whether any classes need to be reorganised in the ontology’s structure.

The research team extracted and logged each piece of participant feedback from Qualtrics ( https://www.qualtrics.com/uk/). The team then discussed how to address each issue raised, recording decisions to revise the ontology based on feedback or the rationale for not making revisions. The HBO was updated based on the discussions.

### Step 5: Testing inter-rater reliability of researchers applying the ontology to annotate research reports

To investigate whether the HBO could be reliably used to classify behaviours, we evaluated the inter-rater reliability (IRR) of researchers applying the updated ontology to annotate (i.e., code) mention of behaviours in 100 papers on the web-based software, ***EPPI-Reviewer*** v4 ( Thomas *et al.*, 2010; Thomas *et al.*, 2020). An open access alternative for this annotation software is PDFAnno ( Shindo *et al.*, 2018). For the ontology to be applicable to a wide range of different behaviours, these papers were chosen to cover (i) studies investigating or exploring behaviours, (ii) behaviour change intervention development and/or evaluation or (iii) descriptions of a model, theory or framework related to behaviour.

These papers were identified by running a search on the database Open Alex ( Priem *et al.*, 2022) in September 2022. The search retrieved papers that cited either the Theoretical Domains Framework ( Cane *et al.*, 2012), a framework synthesising broad influences on behaviour, or the Behaviour Change Techniques Taxonomy v1 (BCTTv1; Michie *et al.*, 2013). These two papers were used as starting point, as they have been a widely cited and used in a protocols, intervention development and evaluations related to behaviours ( Birken *et al.*, 2017; Corker *et al.*, 2023).

As recent papers were more likely to include clearer descriptions of behaviour, only papers published 2 years before the search (2020–2022) were included, resulting in 2532 papers after duplicates were removed. Sets of 30-50 papers’ titles and abstracts, and then full texts were screened until at least 100 eligible papers were identified. The inclusion criteria was to:

- Include a mention of human behaviour, and
- Report this behaviour within the context of (i) a study investigating or exploring behaviour, (ii) an intervention development or evaluation process, or (iii) behavioural model, theory or framework.

Additional details about the method for identifying suitable papers to annotate can be found in: https://osf.io/csw65. Altogether, 110 eligible papers were identified, of which 100 were randomly selected for annotation. The full list of papers annotated can be found in: https://osf.io/rdw9f.

IRR was assessed in two stages. First, 50 papers were independently annotated by two researchers involved in the development of the ontology (PS & GS) on EPPI-Reviewer v4 ( Thomas *et al.*, 2010; Thomas *et al.*, 2020). Fifty papers were selected to provide a 10–15% margin of error around the estimated inter-rater agreement value ( Gwet, 2014). Second, two behaviour change experts unfamiliar with the ontology but with annotation experience (LZ & MS) annotated the remaining 50 papers. IRR was calculated with Krippendorff's alpha ( Hayes & Krippendorff, 2007) using Python 3.6 ( Finnerty & Moore, 2020).

Alpha values above 0.67 indicate acceptable IRR ( Krippendorff, 2009; Krippendorff, 2011), with values below this threshold indicating that the class labels and definitions are likely to be interpreted differently by researchers. If the IRR was lower than 0.67 in an annotation stage, the two annotators reviewed their annotation disagreements across 50 reports and suggested changes to the ontology or its annotation guidance. The wider research team provided feedback on these suggestions, and updates were made to the ontology or annotation guidance accordingly.

### Step 6: Revising classes and adding relations between classes

To structure the classes in the HBO, the research team formally specified hierarchical relations between classes. For such relations, a principle is to ensure that each class has only one parent class. While it is not a strict requirement in ontologies to have a single parent class, it is a recommendation ( Arp *et al.*, 2015), and we adopted the recommendation for the BCIO as far as possible because it makes the ontology easier to maintain and browse. When selecting parent classes for a class in the HBO, behaviours were specified in terms of a behavioural class to which a behaviour *always* belongs, although it may sometimes belong to other classes. For example, ‘writing’ is *sometimes* a ’communication behaviour’, but *always* an ‘object-using behaviour’ (as writing always involves the use of ‘something’ with which to write). Therefore, ‘object-using behaviour’ would be selected as the parent class of ‘writing’ in the HBO.

Additional relations needed to be specified between classes, as a hierarchical classification system on its own would not be adequate to characterise the way that behaviours are described in the research literature. The same class of behaviour (e.g., ‘walking’) can be used to characterise different types of behaviour. For instance, walking on a treadmill could be done purely for physical exercise, whereas walking to a store would be a transportation behaviour. In the ontology, one way of characterising behaviours more precisely is by defining relations between classes, including behavioural attributes. From combinations of these relations, ***logically defined classes*** can be created where needed. For example, different types of walking behaviour can be specified more precisely through logical definitions: ‘walking’ that *occurs in* some ‘park’. In this example, *occurs in* is a defined relation that can link a behaviour to a range of contexts in the Setting Ontology within BCIO ( Norris *et al.*, 2020). Therefore, the research team decided to include a set of relations between classes in the ontology. In accordance with best practice ( Arp *et al.*, 2015), the relations were imported where possible from Basic Formal Ontology (BFO) and the Relations Ontology (RO) (which sits under BFO) ( Smith *et al.*, 2005). Where new relations had to be defined, they were specified as subclasses of relations in BFO or RO.

We also proposed an initial list of ‘behavioural attributes’ by drawing on the ‘Big Question’ addressed by the Human Behaviour-Change Project ( www.humanbehaviourchange.org): “What works, compared with what, for what behaviours, how well, for how long, with whom, in what setting, and why?” ( Michie *et al.*, 2017). This list was also informed by published characteristics of health-related behaviours ( McEachan *et al.*, 2010) and published ‘applied behavioural analysis’ literature ( Cooper *et al.*, 2020). Behavioural attributes were broadly defined as characteristics of a behaviour that help specify it. This definition was purposefully kept broad to identify various details that should be specified about a behaviour and thereby be included in the HBO.

To refine the behavioural attributes, two researchers independently applied these attributes to annotate 50 scales measuring some aspect of behaviour (see details of method to identify measurement scales in: https://osf.io/n7vhb). The researchers compared their annotations and discussed discrepancies, raising issues with the wider research team where necessary. These discussions informed changes to the behavioural attributes, e.g., labels and definitions were updated, or behavioural attributes were added. The research team then discussed how each attribute should be formally captured in the ontology, either as a new class and/or a relation between a behaviour and some other class. Ontological labels and definitions were written for these classes and relations.

To improve the structure and navigability of the ontology, we reviewed the upper-level classes to identify overarching parent classes. Where needed, new overarching classes were developed, labelled, defined and specified as parent classes of relevant classes.

### Step 7: Making the ontology machine-readable and available online

When the HBO content was ready for its initial release, its content was converted from the spreadsheet format into ***Web Ontology Language (OWL)*** ( Antoniou & van Harmelen, 2004) format, using a custom script which uses the ***ROBOT*** ontology toolkit library ( Jackson *et al.*, 2019). The OWL format is a standard format, thereby allowing the ontology to be compatible with other ontologies, and viewed and visualised on ontology software, such as Protégé ( Musen, 2015). A ROBOT template is a comma-separated values (CSV) file that can prepared using spreadsheet software (e.g., Excel) for conversion from spreadsheet columns into OWL language and metadata attributes. In the input template spreadsheet, we used separate columns to capture the unique semantic-free class IDs (e.g., BCIO:01023), labels, definitions, parent class, informal definitions, relations with other classes, comments, examples and synonyms. The OWL version of the HBO is stored on the Human Behaviour Change Project’s project ***GitHub*** repository ( https://github.com/HumanBehaviourChangeProject). The GitHub repository supports ***versioning*** the ontologies and has an ***issue tracker***, which enables ontology users to provide feedback on issues to be addressed in future releases of the ontology. Finding and examining individual classes in the ontology was made possible by building a bespoke website BCIOSearch ( https://BCIOsearch.org). Visualising the hierarchical relations between classes was made possible by building the bespoke BCIOVisualise website ( https://bciovis.hbcptools.org/). These tools are available via the BCIO website ( https://www.bciontology.org/).

## Results

### Step 1: Specifying the scope of the Human Behaviour Ontology

The scope of the HBO was the full range of potentially observable human behaviours investigated in the research literature. This includes partially overlapping behavioural domains relating to immediate survival, hygiene, longer-term health, mental wellbeing, reproduction, transport, teaching and learning, economic activity, social activity, professional activity, environmental sustainability, social interaction, crime, violence, security, leisure, play, religion, culture and the arts.

Building on the definition of behaviour previously generated based on feedback from 24 behavioural science experts ( Davis *et al.*, 2015), the ontological definition of a class labelled ‘individual human behaviour’ was:

*“A bodily process of a human that involves co-ordinated contraction of striated muscles controlled by the brain.”*

The scope of the HBO was anything that fell within, or would be required to characterise classes that fall within, this class.

We excluded beliefs, intentions, decisions, perceptions and feelings. These were classified in the BCIO as mental processes, cognitive representations and dispositions rather than behaviours (see the Mechanism of Action Ontology within BCIO; Schenk *et al.*, 2023). Also excluded from the ontology were physical processes undertaken by bodies that: are not controlled by the brain (although they might be influenced by it) (e.g., spinal reflexes); do not involve striated (voluntary) muscles (e.g., peristalsis); or involve the activities of glands (e.g., sweating, salivating). Thus, for example ‘sleep’ was not in scope but behaviours related to sleep such as ‘going to bed’ and ‘lying down’ were.

### Step 2: Identifying key classes and developing their preliminary definitions

The first iteration of the HBO recognised that a distinction needed to be made between behaviours of individuals and of populations. Ontologically these are different things. Individual occurrences of a behaviour are single processes whereas population behaviours are multiple processes. Population behaviours are characterised in terms of incidence and prevalence (e.g., cigarette smoking prevalence). In principle, any specific occurrence of a behaviour would have a parallel population version. To avoid doubling the number of classes, it was decided to specify all the classes at the level of individual occurrences and focus on the subclasses of ‘individual human behaviour’. However, we noted down ‘population behaviour’ to be added in later steps, to allow relevant subclasses to be created as required in future iterations of the ontology.

In this step, the classes of individual human behaviour were organised in a five-level hierarchical structure containing 85 classes, with 61 classes relating to behaviours and 24 classes related to functions that may be realised by some of these behaviours (e.g., life maintenance, life enhancement, environment management, learning, social integration). Each class was connected to its parent class with an ‘is_a’ relation ( Smith *et al.*, 2005), e.g., ‘alcohol consumption’ *is_a* ‘consumption behaviour’. Behaviours were organised at this stage within 11 upper-level classes: functional, locomotive, posture, sexual, expressive, physical impact, grooming, goal-oriented, interpersonal, socially-evaluated, and object-involving (see these classes in: https://osf.io/v869m).

### Step 3: Refining the ontology by applying it to relevant literature

Based on the 334 example behaviours identified in the research literature, the labels and/or definitions of 51 classes were updated. In addition, 35 classes were added to the ontology. Most of these classes were broad enough to capture more specific examples of behaviour. However, some of these were added due to the need to distinguish between single occurrences of a behaviour and repeated occurrences of the same behaviour (behaviour patterns). While an ‘individual human behaviour’ is a single occurrence, behaviour patterns are multiple processes distributed over time. Behaviour patterns have characteristics such as frequency and temporal patterning (e.g., daily cigarette consumption). To avoid adding too much detail to the ontology, broad classes for behaviour patterns were specified (e.g., ‘behaviour pattern’) and subclasses for tobacco-related behaviour pattern classes were created as an example use case.

Of the 334 example behaviours, 217 could be captured under an existing class in the ontology, and were added as examples of these classes. For example, ‘urinating’ was added as an example of ‘excretion function behaviour’. A further 27 behaviours were allocated as examples for eight new classes in the ontology (e.g., ‘voting’ was added as an example of ‘political behaviour’). The remaining behaviours could not be allocated because they: (i) were too generic (e.g., ‘planning’; 23 behaviours), (ii) did not fit into the current definition of behaviour (e.g., ‘psychomotor agitation’; 18 behaviours), (iii) were duplicates (i.e., the class already existed in the ontology; 16 behaviours), (iv) were a grouping of behaviours rather than a single behaviour (e.g., ‘activities of daily living’; 13 behaviours), or (v) were too specific to be useful examples and were otherwise captured in the ontology (e.g., ‘switching on’; 6 behaviours) (see details of the behaviours that were recorded as examples or not allocated in: https://osf.io/7scz6).

At the end of this step, the ontology consisted of 120 classes across eight hierarchical levels (see classes in: https://osf.io/v9ymr).

### Step 4: Expert stakeholder review

Ten experts (eight behavioural scientists and two ontology experts) agreed to participate in the review and met the eligibility criteria. These experts worked in institutions based in: Australia (n = 1), Chile (n = 1), France (n = 1), Ireland (n = 1), United Kingdom (n = 2) and United States (n = 4). Participant comments and the steps taken to address each comment or the rationale for not addressing specific comments were recorded (see this record in: https://osf.io/r8g6a). In response to the comments, 25 class labels, 42 class definitions and the parent classes of 26 classes were updated. For 19 classes, examples were added or changed; for 23, comments were added or changed; for five, informal definitions were added.

Fifty-one classes were removed from the ontology as it was considered that they were underspecified or overcomplicated the ontology. This included removing the formal use of functions and most classes related to these. In particular, the subclasses of function were removed from the ontology. However, function-related behaviours were kept as classes, if they were not captured by other behavioural classes.

The classes ‘outcome behaviour’ and ‘goal-related behaviour’ were removed from the ontology as these constructs were judged to be better represented using the relations ‘has behavioural goal’ and ‘has behavioural outcome’ (see details in Step 6).

To address participants’ comments regarding missing classes, 59 classes were added, resulting in an ontology with 128 classes (see updated version of the ontology in: https://osf.io/awgtp). As the remaining three function-related classes in the ontology did not capture behaviours, this left 125 classes to be included in the next step.

### Step 5: Testing inter-rater reliability of researchers applying the ontology to annotate research reports

The inter-rater reliability for two researchers familiar with the ontology was 0.63 which was marginally lower than the set threshold of 0.67 (see https://osf.io/95rbx). Annotation discrepancies were examined, and changes to the ontology and the ***annotation guidance manual*** were made (see details: https://osf.io/yrnph). Most changes were to the annotation guidance, but examples were added or changed for three classes and a synonym was added to one class. Four classes (e.g., ‘alcohol consumption’) were added to the ontology.

Using the updated version of the ontology and annotation guidance, two researchers unfamiliar with the ontology had an acceptable inter-rater reliability (α = 0.74) (see https://osf.io/ktfys). The ontology at this stage had 129 behavioural classes, with 34 serving as upper-level classes such as ‘antisocial behaviour’, ‘economic behaviour’, ‘environmental system management behaviour’, ‘personal bodily care behaviour’ and ‘providing healthcare’. Including the three function-related classes, the ontology had 132 individual human behaviour classes in total at the end of this step (see https://osf.io/rxcuf).

### Step 6: Revising classes and adding relations between classes

It was apparent that 34 upper-level classes of behaviour was unwieldy and it was possible to group these into just nine classes relating to: (1) experiences (e.g., enjoyment behaviour, such as playing), (2) expressive (e.g., laughing), (3) harm (e.g., self-injury behaviour), (4) health (e.g., utilising healthcare), (5) life function (e.g., breathing behaviour), (6) interacting with materials and objects (e.g., consumption behaviour), (7) personal bodily care (e.g., bodily hygiene behaviour), (8) position and location (e.g., travel behaviour) and (9) the social environment (e.g., human communication behaviour) (see definitions and their immediate subclasses in Table 3). Of the nine classes, seven were new classes added to the ontology.

**Table 3.** Upper-level behavioural classes and their immediate subclasses in the HBO.

Upper-level behavioural classes (Level 1)	Behavioural subclasses (Level 2)	Definition
experience-related behaviour *BCIO:050436*		An individual human behaviour that relates to something the person experiences.
	avoidance behaviour *BCIO:036073*	An experience-related behaviour that involves taking defensive action in order to avoid stimuli judged as aversive by the person.
	creative expression behaviour *BCIO:036021*	An experience-related behaviour that involves consciously using some capability to create or shape an aspect of the environment to express an idea or emotion.
	distress minimisation behaviour *BCIO:050421*	An experience-related behaviour that involves avoiding, reducing or escaping anxiety, stress, sorrow, shame and unhappiness.
	enjoyment behaviour *BCIO:036047*	An experience-related behaviour that is performed to experience pleasure or satisfaction.
	learning behaviour *BCIO:036008*	An experience-related behaviour that involves improving knowledge or skill.
	mind-body behaviour *BCIO:036041*	An experience-related behaviour that aims to create a sense of interconnectedness between the mind and the body.
	sexual behaviour *BCIO:036030*	An experience-related behaviour that involves sexual arousal.
	spiritual behaviour *BCIO:036112*	An experience-related behaviour that is performed in line with a belief system that is grounded in reverence for a supernatural power or powers and provides meaning in life.
expressive behaviour *BCIO:050457*		An individual human behaviour that conveys a thought or feeling.
	crying *BCIO:050456*	An expressive behaviour that involves tears, and facial expressions of distress.
	creative expression behaviour *BCIO:036021*	An expressive behaviour that involves consciously using some capability to create or shape an aspect of the environment to express an idea or emotion.
	facial expression behaviour *BCIO:050458*	An expressive behaviour that involves the muscles of the face.
	gesticulatory expressive behaviour *BCIO:050459*	An expressive behaviour involving a movement of the limbs, head or torso.
	human communication behaviour *BCIO:036034*	An expressive behaviour that involves the transmission of information between two or more people.
	laughing *BCIO:050374*	An expressive behaviour showing elation, amusement or scorn by means of facial expressions and repeated sharp exhalations.
	vocalisation behaviour *BCIO:050442*	An expressive behaviour involving vibration of the vocal chords.
harmful behaviour *BCIO:036075*		An individual human behaviour that causes net harm.
	harmful behaviour to others *BCIO:050398*	A harmful behaviour that involves interacting with another animal * and thereby causing harm to its health, wellbeing or social functioning.
	self-injury behaviour *BCIO:036014*	A harmful behaviour that involves intentionally causing oneself physical harm.
health-related behaviour *BCIO:050437*		An individual human behaviour that relates to health of oneself or others.
	physical performance behaviour *BCIO:036042*	A health-related behaviour that involves maintenance or improvement of flexibility, strength, balance or cardiovascular fitness.
	providing healthcare *BCIO:050413*	A health-related behaviour that involves assessing, monitoring, improving or maintaining an aspect of another person’s health.
	self-monitoring an aspect of health *BCIO:050410*	A health-related behaviour that uses a method to monitor and record an indicator of one’s health or wellbeing.
	utilising healthcare *BCIO:050399*	A health-related behaviour that involves interacting with a healthcare provider in order to assess, monitor, improve or maintain an aspect of one's health.
life function-related behaviour ^#^ *BCIO:050438*		An individual human behaviour that serves vital bodily functions.
	breathing behaviour *BCIO:036057*	A life function-related behaviour involves providing an appropriate level of oxygenation to body tissues.
	excretion behaviour *BCIO:036054*	A life function-related behaviour that involves eliminating excess or harmful chemicals produced by bodily functions.
	reproductive behaviour *BCIO:036056*	A life function-related behaviour that involves producing offspring based on combining DNA of two or more people.
material entity-related behaviour *BCIO:050439*		An individual human behaviour that relates to a material entity.
	consumption behaviour ^†^ *BCIO:036061*	A material entity-related behaviour that involves ingesting material into the body.
	environmental system management behaviour *BCIO:036007*	A material-entity related behaviour that involves creating, maintaining, adapting or destroying aspects of the physical or social environment system.
	object-using behaviour *BCIO:036027*	A material-entity related behaviour that uses a non-living object.
	physical contact behaviour *BCIO:050426*	A material-entity related behaviour that makes physical contact with something.
personal bodily care behaviour *BCIO:036024*		An individual human behaviour that relates to the person’s hygiene, comfort or appearance.
	appearance-based bodily behaviour *BCIO:050372*	A personal bodily care behaviour that attends to making changes to one's body to achieve a desired appearance.
	bodily hygiene behaviour *BCIO:050368*	A personal bodily care behaviour that attends to hygiene by cleaning or washing oneself or parts of the body.
	dressing behaviour *BCIO:050371*	A personal bodily care behaviour that involves wearing clothes providing comfort and protecting oneself from ambient conditions.
	sun protective behaviour *BCIO:050411*	A personal bodily care behaviour that involves protecting one's skin or eyes from the damaging effects of the sun.
position-related behaviour *BCIO:050440*		An individual human behaviour that relates to the enactor's posture or location.
	locomotive behaviour *BCIO:036026*	A position-related behaviour in which muscles are used by a person to move themselves relative to the immediate environment or part of it.
	posture behaviour *BCIO:036029*	A position-related behaviour that involves adopting a body configuration in relation to the immediate environment.
	travel behaviour *BCIO:036059*	A position-related behaviour that involves changing physical location.
socially-related behaviour *BCIO:050441*		An individual human behaviour that relates to the social environment.
	antisocial behaviour *BCIO:036072*	A socially-related behaviour that a population judges to be is contrary to the laws or accepted current norms of social conduct within a specific social context and causes annoyance and or disapproval in others.
	economic behaviour *BCIO:036035*	A socially-related behaviour that involves the production, acquisition, distribution or exchange of money, goods or services.
	inter-personal behaviour ^‡^ *BCIO:036025*	A socially-related behaviour that involves an interaction between two or more people.
	nurture behaviour *BCIO:036086*	A socially-related behaviour that involves meeting the physical, psychological or social needs of another living being to promote its development.
	political behaviour *BCIO:036089*	A socially-related behaviour that aims to bring about or oppose political or social change.
	pro-social behaviour *BCIO:036066*	A socially-related behaviour that a population judges to accord with current norms of positive social conduct.
	social organisation behaviour *BCIO:036011*	A socially-related behaviour that involves a person contributing to the functioning of a social structure or a person in relation to a social structure.

^*^The term ‘animal’ is used in ontologies to refer to any animal, including humans, who are categorised as animals. ^#^the nutrition function is covered by consumption behaviour. †The parent class of ‘consumption behaviour’ was changed from ‘object-using behaviour’ to ‘material entity-related behaviour’. ‡For the class ‘human communication behaviour’, two parent classes were recorded: ‘interpersonal behaviour’ and ‘expressive behaviour’. However, in the hierarchy, its parent class will be shown as ‘expressive behaviour’.

Changes were made to some class definitions to specify them more clearly (see the log of changes to the upper-level classes in https://osf.io/2wdsa). In addition, six classes (e.g., ‘crying’, ‘facial expression behaviour’ and ‘vocalisation behaviour’) were added to more fully capture expressive behaviours. The class ‘impulsive behaviour’ was removed, and ‘impulsiveness’ was noted down as a potential behaviour attribute instead. The class ‘animal life function’ was also removed from the ontology, as the HBO is specific to humans. In addition, the classes for ‘population behaviour’, ‘population behavioural pattern’ and ‘human behaviour’ (a logical class to capture any human behaviour, including individual and population behaviours) were added. This resulted in 146 classes.

***Adding behavioural attributes and relations*.** To capture additional characteristics of behaviour that help specify them (e.g., timing or location), the research team generated an initial list of attributes (see https://osf.io/dmu52). Examples were location, duration, frequency and physical exertion required during the performance of behaviours. Two researchers applied these attributes to annotate items in measures of behaviour; annotation issues were recorded and resolved (see https://osf.io/945nb). This resulted in 18 putative behavioural attributes, with the addition of three new attributes: ‘behaviour starting timepoint’, ‘behaviour end timepoint’ and ‘behaviour target person’. Through additional reviews of the attributes, the research team:

1. Captured 22 classes based on the list of behavioural attributes to include in the ontology, and further developed nine classes (‘behaviour attribute’, ‘impulsiveness’ ‘reflectiveness’, ‘intentionality’, ‘behaviour disposition’, ‘abstinence from a behaviour’, ‘abstinence duration’, ‘abstinence start point’, ‘abstinence end point’), thereby adding 31 classes to the ontology.
2. Represented nine relations based on the list of attributes, and further specified four relations (e.g., ‘has behavioural goal’) between ‘individual human behaviour’ and ontology classes lying outside of this ontology, and one to specify abstinence duration. (‘has abstinence duration’) resulting in 14 non-hierarchical relations being specified,
3. Removed two attributes that did not capture sufficiently clear or unique aspect of behaviour.

Where no relevant relation could be identified from the Relation Ontology ( Smith *et al.*, 2005), such as for ‘has behavioural goal’ and ‘has behavioural outcome’, new relations were developed. These relations were specified to link a behaviour to a particular external class. For instance, to capture that the outcome of behaviour is the emotion ‘happiness’ from the Emotion Ontology ( Hastings *et al.*, 2011), the following relation can be specified: ‘individual human behaviour’ *has_behavioural_outcome* ‘happiness’ ( http://purl.obolibrary.org/obo/MFOEM_000042). As the outcomes and goals of behaviours are context-dependent, these relations were not linked to specific classes, but were designed to be used flexibly by ontology users.

The resulting ontology had 177 classes, organised onto eight hierarchical levels, made up as follows (see Human Behaviour Ontology version 1: https://osf.io/dc7jm/):

1.   The class ‘individual human behaviour’ and 127 classes organised under it (128 classes overall).

2.   The class ‘behavioural attribute’ and 20 classes under it (21 classes overall; see Table 4).

3.   The 10 classes used to characterise behaviours or abstinence from behaviour (see Table 5).

4.   The class ‘function’ and its subclass ‘human life function’ (two classes).

5.   The class ‘individual human behaviour pattern’, its two upper-level classes (i.e., its parent class and the subsequent upper-level class), and five lower-level classes illustrating its use in relation to tobacco use behaviour (eight classes overall).

6.   The class ‘individual human behaviour change’ and four lower-level classes illustrating the use of the class in relation to tobacco use cessation (five classes overall).

7.   The ‘population behaviour’, ‘population behaviour pattern’ and ‘human behaviour’ classes (three classes).

**Table 4.** Behavioural attributes in the Human Behaviour Ontology.

Label	Definition	Parent class	Informal definition	Comment
behavioural attribute *BCIO:050435*	A process attribute of an individual human behaviour.	process attribute	An attribute of a behaviour.	-
behavioural form *BCIO:050430*	A behavioural attribute that is the physical way in which a behaviour is enacted.	behavioural attribute	The way in which the behaviour is performed, including the shape of one’s muscles and skeletal alignment during the behaviour.	-
physical exertion expended on a behaviour *BCIO:050432*	A behavioural attribute that is the level of musculoskeletal work expended on the behaviour to be enacted.	behavioural attribute	The physical effort required to perform a behaviour.	-
high physical exertion expended on behaviour *BCIO:050465*	Physical exertion expended on a behaviour that is high.	physical exertion expended on a behaviour		High physical exertion will mean different things depending on how this concept is operationalised. Therefore, when using this class, you would need to operationalise it for the relevant context (e.g., specify what high exertion means based on the measurement you use).
moderate physical exertion expended on behaviour *BCIO:050473*	Physical exertion expended on a behaviour that is medium.	physical exertion expended on a behaviour		Moderate physical exertion will mean different things depending on how this concept is operationalised. Therefore, when using this class, you would need to operationalise it for the relevant context (e.g., specify what moderate exertion means based on the measurement you use).
low physical exertion expended on behaviour *BCIO:050469*	Physical exertion expended on a behaviour that is low.	physical exertion expended on a behaviour		Low physical exertion will mean different things depending on how this concept is operationalised. Therefore, when using this class, you would need to operationalise it for the relevant context (e.g., specify what low exertion means.
mental exertion expended on a behaviour *BCIO:050431*	A behavioural attribute that is the level of mental effort expended on the behaviour to be enacted.	behavioural attribute	The mental effort required to perform a behaviour.	-
high mental exertion expended on a behaviour *BCIO:050464*	Mental exertion expended on a behaviour that is high.	mental exertion expended on a behaviour	High mental exertion will mean different things depending on how this concept is operationalised. Therefore, when using this class, you would need to operationalise it for the relevant context (e.g., specify what high exertion means based on the measurement you use).	
moderate mental exertion expended on a behaviour *BCIO:050472*	Mental exertion expended on a behaviour that is medium.	mental exertion expended on a behaviour	Moderate mental exertion will mean different things depending on how this concept is operationalised. Therefore, when using this class, you would need to operationalise it for the relevant context (e.g., specify what moderate exertion means based on the measurement you use).	
low mental exertion expended on a behaviour *BCIO:050468*	Mental exertion expended on a behaviour that is low.	mental exertion expended on a behaviour	Low mental exertion will mean different things depending on how this concept is operationalised. Therefore, when using this class, you would need to operationalise it for the relevant context (e.g., specify what low exertion means based on the measurement you use).	
cognitive exertion expended on a behaviour *BCIO:050433*	Mental exertion expended on a behaviour where the exertion involves cognitive processes.	mental exertion expended on a behaviour	The effort relating to thinking required to perform a behaviour.	-
high cognitive exertion expended on a behaviour *BCIO:050462*	Cognitive exertion expended on a behaviour that is high.	cognitive exertion expended on a behaviour		High cognitive exertion will mean different things depending on how this concept is operationalised. Therefore, when using this class, you would need to operationalise it for the relevant context (e.g., specify what high exertion means based on the measurement you use).
moderate cognitive exertion expended on a behaviour *BCIO:050470*	Cognitive exertion expended on a behaviour that is medium.	cognitive exertion expended on a behaviour		Moderate cognitive exertion will mean different things depending on how this concept is operationalised. Therefore, when using this class, you would need to operationalise it for the relevant context (e.g., specify what moderate exertion means based on the measurement you use).
low cognitive exertion expended on a behaviour *BCIO:050466*	Cognitive exertion expended on a behaviour that is low.	cognitive exertion expended on a behaviour		Low cognitive exertion will mean different things depending on how this concept is operationalised. Therefore, when using this class, you would need to operationalise it for the relevant context (e.g., specify what low exertion means based on the measurement you use).
emotional management exertion expended on a behaviour *BCIO:050434*	Mental exertion expended on a behaviour where the exertion involves control over emotions or their expression.	mental exertion expended on a behaviour	The effort a person has to exert to manage their emotions when performing a behaviour.	-
high emotional management exertion expended on a behaviour *BCIO:050463*	Emotional management exertion expended on a behaviour that is high.	emotional management exertion expended on a behaviour		High emotional management exertion will mean different things depending on how this concept is operationalised. Therefore, when using this class, you would need to operationalise it for the relevant context (e.g., specify what high exertion means based on the measurement you use).
moderate emotional management exertion expended on a behaviour *BCIO:050471*	Emotional management exertion expended on a behaviour that is medium.	emotional management exertion expended on a behaviour		Moderate emotional management exertion will mean different things depending on how this concept is operationalised. Therefore, when using this class, you would need to operationalise it for the relevant context (e.g., specify what moderate exertion means based on the measurement you use).
low emotional management exertion expended on a behaviour BCIO:050467	Emotional management exertion expended on a behaviour that is low.	emotional management exertion expended on a behaviour		Low emotional management exertion will mean different things depending on how this concept is operationalised. Therefore, when using this class, you would need to operationalise it for the relevant context (e.g., specify what low exertion means based on the measurement you use).
impulsiveness *BCIO:036076*	A behavioural attribute that is to what extent the behaviour is a direct emotional, habitual or instinctive reaction to something.	behavioural attribute	How far a behaviour is enacted without thinking.	This class is a dimension and can be construed as the obverse of reflectiveness and so operationalised in terms of acting without thinking.
reflectiveness *BCIO:050444*	A behavioural attribute that is to what extent the behaviour is a response to reflective motivation or thinking.	behavioural attribute	How far a behaviour is enacted after thinking about it and its consequences.	This class is a dimension and involves any conscious thought processes that lead to a behaviour in some way, even if those processes are themselves influenced by emotional processes and biases.
intentionality *BCIO:050447*	A behavioural attribute that is the extent to which the behaviour is caused by a behavioural intention.	behavioural attribute	How far a behaviour is enacted as a direct result of a conscious intention to enact it.	This class is a dimension and is differentiated from reflectiveness because a behaviour may be fully intentional but involve little reflective thought, e.g., when driving carelessly. In this class the intention relates to the behaviour itself. If a person intends to do one thing but accidentally does something else it does not count as intentional in this class, e.g., if someone intends to injure someone else and ends up killing them, that would not count as intentionally killing them.

**Table 5.** Additional classes for characterising behaviours or abstinence from behaviour.

Label	Definition	Parent class	Informal definition
number of behavioural occurrences *BCIO:050429*	A data item that is about the number of times a behaviour has occurred.	data item	Number of times a person performs a behaviour.
behavioural frequency *BCIO:050428*	A data item that is about the number of times a behaviour occurs in a time period.	data item	Number of times a person performs a behaviour within a specific period.
behavioural duration *BCIO:050455*	A temporal interval within which an individual human behaviour occurs.	one-dimensional temporal region (temporal interval)	The time between the start and end of a behaviour.
behaviour starting point *BCIO:050454*	A temporal region that is the start of an individual human behaviour.	temporal region	A time point when a behaviour starts.
behaviour end point *BCIO:050453*	A temporal region that is the end of an individual human behaviour.	temporal region	A time point when a behaviour ends.
abstinence from a behaviour *BCIO:050451*	A personal attribute in which a person does not engage in a behaviour during a time period.	personal attribute	Not performing a behaviour for some period of time.
abstinence duration *BCIO:050449*	A temporal interval during which a person is abstinent from a behaviour.	one-dimensional temporal region (temporal interval)	The time a person is abstinent from a behaviour.
abstinence start point *BCIO:050452*	A temporal region that is the start of an abstinence period.	temporal region	A time point when a person starts being abstinent from a behaviour.
abstinence end point *BCIO:050450*	A temporal region that is the end of an abstinence period.	temporal region	A time point when a person stops abstaining from a behaviour.
behavioural disposition *BCIO:050416*	A bodily disposition that is realised as some behaviour.	bodily disposition	A tendency to behave in a particular way.

It also had 15 relations: the ‘is a’ relation, the ‘has behavioural attribute’ relation, and 13 other relations (see Table 6). It was determined that, in order to represent many of the behaviours of interest in behavioural science, it would be necessary to create classes using logical expressions that combine classes and relations ( Köhler *et al.*, 2011). For example, a class ‘moderate physical activity’ would be defined by the expressions (‘physical performance behaviour’) and (‘has behavioural attribute’ ‘moderate physical exertion expended on a behaviour’).

**Table 6.** Relations in the Human Behaviour Ontology.

Label	Definition * (domain/range ^#^)	Parent class	Informal definition
starts *RO:0002223*	inverse of starts with (occurrent/occurrent)	temporally related to *RO:0002222*	A relation that specifies a timepoint at which a behaviour starts.
ends *RO:0002229*	inverse of ends with (occurrent/occurrent)	temporally related to *RO:0002222*	A relation that specifies a timepoint at which a behaviour ends.
occupies temporal region *BFO:0000155*	p occupies_temporal_region t. This is a primitive relation between an occurrent p and the temporal region t upon which the spatiotemporal region p occupies_spatiotemporal_region projects. (occurrent/occurrent)	exists at *BFO:0000108*	A relation that specifies the duration of a behaviour.
happens during *RO:0002092*	X happens_during Y if: (start(Y) before_or_simultaneous_with start(X)) AND (end(X) before_or_simultaneous_with end(Y)). (occurrent/occurrent)	temporally related to *RO:0002222*	A relation that specifies that the behaviour occurs during a specified temporal interval.
has abstinence period *BCIOR:000009*	A relation that links abstinence from a behaviour to a temporal region during which this personal attribute is true. (abstinence from a behaviour/abstinence duration)	relation	A relation that specifies the duration for which a person is abstinent from a behaviour.
occurs in *BFO:0000066*	b occurs_in c =def b is a process and c is a material entity or immaterial entity & there exists a spatiotemporal region r and b occupies_spatiotemporal_region r.& for all(t) if b exists_at t then c exists_at t & there exist spatial regions s and s’ where & b spatially_projects_onto s at t& c is occupies_spatial_region s’ at t& s is a proper_continuant_part_of s’ at t (occurrent/independent continuant)	relation	A relation that specifies a place where a behaviour happens.
has behavioural attribute *BCIOR:000010*	A relation that links an individual human behaviour to a behavioural attribute. (individual human behaviour/behavioural attribute)	relation	A relation that specifies the characteristics of a behaviour, such as its frequency, the effort it requires, its form and whether it’s intentional.
serves behavioural function *BCIOR:000011*	Realises the human life function of an individual human behaviour. (individual human behaviour/human life function)	realises *BFO:0000055*	A relation that specifies the biological or social function of a behaviour.
has behavioural goal *BCIOR:000012*	Causally influenced by a cognitive representation of something the behaviour could bring about. (individual human behaviour/cognitive representation)	causally influenced by *RO:0002559*	A relation that specifies the goal (as a cognitive representation) of a behaviour.
has behavioural outcome *BCIOR:000013*	Causally relation between two entities in which a behaviour brings into existence, causes to occur, destroys, prevents from occurring, or changes an entity. (individual human behaviour/entity)	causal relation between entities *RO:0002506*	A relation that specifies the outcome of a behaviour.
is enacted by *BCIOR:000014*	Has participant that relates a behaviour to the person enacting the behaviour. (individual human behaviour/person)	has participant *RO:0000057*	A relation that specifies a person performing a behaviour.
uses *BCIOR:000015*	Has participant that relates a behaviour to a material entity that the person enacting the behaviour intends to enable or facilitate the behaviour. (individual human behaviour/material entity)	has participant *RO:0000057*	A relation that specifies some object or substance that is used in a behaviour.
has behavioural companion *BCIOR:000016*	Has participant that relates a behaviour to another sentient being that accompanies the person enacting the behaviour. (individual human behaviour/animal)	has participant *RO:0000057*	A relation that specifies a person or animal with whom the person(s) performs the behaviour.
has behavioural target *BCIOR:000017*	Has participant that relates the behaviour to an object that the person enacting the behaviour intends to influence. (individual human behaviour/person)	has participant *RO:0000057*	A relation that specifies an object, person or animal that a person is trying to influence with their behaviour.

^*^Definitions of relations imported from other ontologies can appear obscure, technical or tautological. We decided to import them anyway and provide informal definitions to help users to grasp their meaning. ^#^Domain refers to classes that can be the subject of a relation. Range refers to classes that can be the object of a relation.

### Step 7: Making the ontology machine-readable and available online

The revised version of the HBO, with its 177 classes and 15 relations, was deployed on OSF ( https://osf.io/dc7jm/), GitHub ( https://github.com/HumanBehaviourChangeProject/ontologies/tree/master/Behaviour) and individual items were made accessible through BCIO Search ( https://www.bciosearch.org/) and visualised through BCIOVisualise ( https://bciovis.hbcptools.org/). The ontology is a live document, and further classes and relations will be added and updated on the ontology’s versions on GitHub, BCIOSearch and BCIOVisualise. The ontology can be applied to behaviour change intervention reports with an annotation guidance, refined in Step 5 (available at https://osf.io/6e2c7). A bespoke BCIO website ( https://www.bciontology.org/) has been created to provide easy access to ontology tools and to bring together information about all the ontologies, including the HBO, into one place.

## Discussion

The Human Behaviour Ontology (HBO) consisted of 177 classes and 15 relations. The classes were organised onto eight hierarchical levels to specify behaviours and details about them, as part of the BCIO ( Michie *et al.*, 2017; Michie *et al.*, 2020). The upper-level subclasses of ‘individual human behaviour’ were: (1) experience-related behaviour, (2) expressive behaviour, (3) harmful behaviour, (4) health-related behaviour, (5) life function-related behaviour, (6) material entity-related behaviour, (7) personal bodily care behaviour, (8) position-related behaviour, and (9) socially-related behaviour. In addition, classes to characterise behaviours (e.g., ‘behavioural attribute’ and ‘number of behavioural occurrences’), and to broadly capture individual human behaviour patterns, population behaviour and population behaviour patterns and abstinence from behaviour were included. Only a small fraction of behaviours that can be conceived of, or used in behavioural research, were included at this stage, but the ontology was designed to provide a framework that could form a coherent basis for defining any of these, either as simple subclasses of behaviours in the existing ontology or as logically defined classes in which classes and relations are combined to form expressions.

Inter-rater reliability was found to be α = 0.63 for those familiar with the ontology and, following updates to the ontology and annotation guidance, acceptable for those unfamiliar with the ontology (α = 0.74). This suggests that the HBO and associated annotation guidance can be applied with at least acceptable consistency.

Several of the classification systems of behaviour identified as part of this study are useful for specifying behaviours within a certain domain, e.g., the International Classification of Health Interventions (ICHI; Fortune *et al.*, 2021) for behaviours relating to health. Building on these frameworks, the scope of the HBO is more extensive, covering behaviours relating to various domains, such as inter-personal and social dynamics, environment, and economics. The HBO can thus serve as a shared language to clearly specify, label and define behaviours when investigating and reporting them. For instance, by using this ontology when writing protocols, researchers can describe behaviours they are intending to investigate more precisely and use unique identifiers to unambiguously specify their target behaviour(s). With its extensive behavioural classes and detail, the ontology can also support categorising behaviours more precisely when synthesising evidence from various sources and predicting outcomes of interventions. The ontology’s technical facilities also enable developing algorithms for searching, information extraction and prediction about behaviour ( Hastings *et al.*, 2023; Michie *et al.*, 2017). Therefore, the ontology presents a basis for a potentially unifying classification about behaviour that is also computer-readable, with opportunities for ontology users to provide feedback to refine the classes and structure ( Arp *et al.*, 2015; He *et al.*, 2018).

The HBO forms part of the BCIO, currently comprising 11 other component ontologies: behaviour change techniques ( Marques *et al.*, 2023), mechanisms of action ( Schenk *et al.*, 2023), mode of delivery ( Marques *et al.*, 2020), source of delivery ( Norris *et al.*, 2021), setting ( Norris *et al.*, 2020), style of delivery ( Wright *et al.*, 2023), dose (in preparation), schedule of delivery (in preparation), engagement (in preparation), fidelity (in preparation), and target population (in preparation). These ontologies can be used together to organise and synthesise detailed evidence about various aspects of behaviour change intervention scenarios and their evaluations. Classes in the HBO can also be linked and reused by ontologies beyond the BCIO, such as the Addiction Ontology ( Hastings *et al.*, 2020).

As part of the Human Behaviour-Change Project, a foundry for ontologies in behavioural and social sciences has been established ( https://www.bssofoundry.org/). Collaborations formed through the BSSO Foundry can be used to support the development of alignments between ontological frameworks on behaviour. For instance, the TURBBO Project has reused some classes from the HBO in their ontology, which specifies relations between behaviours ( https://sites.google.com/sheffield.ac.uk/turbbo/additional-resources#h.4k2unbgh9vh3). In turn, the relations specified and refined in the TURBBO Project can inform enhancements to the relations between HBO classes. More specific ontologies in the field, e.g., about physical activity ( Braun *et al.*, 2023; Kim *et al.*, 2019), could use the HBO’s classes (e.g., ‘physical performance behaviour’), where relevant, and add more granular classes needed for their application (e.g., swimming).

### Strengths and limitations

A strength of the HBO’s development is that it drew on diverse resources, including existing behavioural frameworks and other ontologies, published studies on behaviours and expert feedback. Thus, a range of perspectives was considered when developing and organising the 177 classes included in the ontology. This means that the HBO is likely to be a useful tool to a wide audience interested in behavioural science ( Amith *et al.*, 2018; http://obofoundry.org/principles/fp-010-collaboration.html). Another strength was structuring the HBO by drawing on the Basic Formal Ontology ( Arp *et al.*, 2015), as this enables future collaborations with other ontology developers using the same upper-level structure (e.g., Carlier *et al.*, 2022; Hastings *et al.*, 2020). The use of ontological relations, beyond the hierarchical relations used in taxonomies, also allows users to characterise behaviours in detail. Some examples of applying the ontology’s classes are provided in Table 7. However, it should be noted that ontology users might need to suggest new classes to capture the exact information they are interested in.

**Table 7.** Examples for using classes and relations in the HBO and other ontologies to specify behaviours in detail.

No	Description of behaviour	Behaviour expressed through formal relations between classes
1	A person participates in physical therapy in a healthcare setting for three hours.	• participating in physical therapy (BCIO:050405) ***is enacted by*** (BCIOR:000014) person (MF:0000016) • participating in physical therapy (BCIO:050405) ***occurs in*** (BFO:0000066) health care facility (OMRSE:00000102) • participating in physical therapy (BCIO:050405) ***occupies temporal region*** behavioural duration (BCIO:050455)
2	A woman walks in a park four times a week with her friend, which increases her happiness.	• walking (BCIO:036108) ***is enacted by*** (BCIOR:000014) person (MF:0000016) ***has attribute*** (RO:0000053) female gender (BCIO:015099) • walking (BCIO:036108) ***occurs in*** (BFO:0000066) park (ENVO:00000562) • walking (BCIO:036108) ***has behavioural companion*** (BCIOR:000016) person (MF:0000016) ***has role*** (RO:0000087) friend (BCIO:010101) • walking (BCIO:036108) ***has behavioural outcome*** **(**BCIOR:000013) happiness (MFOEM:000042)
3	A healthcare professional prescribes a medication to a patient in order for him/her/them to fully recover from their condition.	• prescribing medication (BCIO:050355) ***is enacted*** by person (MF:0000016) ***has role*** (RO:0000087) health professional (BCIO:010008) • prescribing medication (BCIO:050355) ***has behavioural target*** (BCIOR:000017) person ***has role*** (RO:0000087) patient role (OBI:0000093) • prescribing medication (BCIO:050355) ***has behavioural goal*** complete remission (OGMS:0000120)

When developing the ontology, a key challenge was deciding how much detail needed to be added to the ontology without making it too complex and thereby difficult to use. Complex ontologies developed as part of the BCIO, such as the Mode of Delivery Ontology ( Marques *et al.*, 2020) and the Mechanism of Action Ontology ( Schenk *et al.*, 2023), were found to be more difficult to reliably apply than ontologies with simpler structures, such as the Intervention Source ( Norris *et al.*, 2021) and Setting ( Norris *et al.*, 2020) Ontologies. For this reason, we attempted to capture behaviours through broad classes, such as economic and political behaviour. However, more granular classes will be needed when reporting or synthesising information about specific behavioural domains. The examples recorded for these behavioural classes in the HBO can provide a starting point for ontology users to suggest new classes on the GitHub repository in the future. Moreover, other ontology developers could expand on these behavioural classes for their specific applications, and suggest additions to the HBO. We encourage all users who want to add more granular classes to suggest them to the HBO team, through the GitHub Issues Tracker ( https://github.com/ontology-tools/onto-spread-ed/issues) or the contact facility on the BCIO website ( https://www.bciontology.org/)

As behaviours can be classified in various ways that are useful in different contexts, there is often overlap between classes. For instance, the class ‘walking’ is always a ‘locomotive behaviour’ but can sometimes also be a ‘travel behaviour’ or a ‘physical performance behaviour’. We only specified parent-child class relations (‘is_a’) if this relation would always hold, e.g., ‘walking’ is_a ‘locomotive behaviour’. Our annotation guidance can support ontology users to consistently apply these classes, e.g., annotating the most specific applicable class or annotating two classes where relevant to capture more information about a behaviour. Such guidance can be tailored based on the aims of a particular research project. Finally, while the relations between behaviours and other classes were discussed in detail and iteratively refined, they were not tested in a specific use case. Future studies would need to investigate to what extent these relations can be understood and reliably applied, particularly by users who are new to ontologies.

This is the first published version of the ontology. The development and maintenance of an ontology is an iterative process; no ontology is ever ‘finished’. The HBO will continue to evolve and is intended to provide a framework within which new classes can be added as required, or definitions can be updated. In some cases, based on the sources we drew on, behaviours have been defined to a level of granularity that would be adequate to characterise a behaviour while in others, classes need to be added as required. We hope that other domain experts will develop more parts of the HBO. To contribute new classes to the ontology, users should suggest the new class by creating an ‘Issue’ on the GitHub portal for the BCIO ( https://github.com/HumanBehaviourChangeProject/ontologies/issues). As the content of ontologies can change over time, we recommend that ontology users report the publication date of the ontology version that they applied to their work. Users should also always report the unique identifier since class labels and definitions can change over time (e.g., based on user feedback or new scientific evidence), but the unique identifier remains the same.

## Conclusion

The HBO is a coherent classification framework that characterises a wide range of human behaviours in the context of behaviour change interventions and beyond. This ontology can be used for detailed and precise reporting, evidence synthesis about behaviours and predicting intervention outcomes. Through refinements based on feedback from its users and collaborations with the developers of other behavioural ontologies, the HBO will become more reflective of wider views about behaviour. This ontology contributes to building a more unified and clear language to communicate about behaviours and hence advancing the science of behaviour and behaviour change.

## Ethics and consent

For this stakeholder review, ethical approval was granted by University College London’s Research Ethics Committee (CEHP/2020/579) in February 2020. Participants provided informed written consent via an online Qualtrics survey before starting the review.

## Data Availability

Open Science Framework: Human Behaviour-Change Project. https://doi.org/10.17605/OSF.IO/EFP4X ( West *et al.*, 2020) This project contains the following underlying data: Expert feedback on Human Behaviour Ontology; Raw feedback received from behavioural science and ontology experts; https://osf.io/r8g6a It should be noted that the stakeholders who are named in this section provided permission to be named. Open Science Framework: Human Behaviour-Change Project. https://doi.org/10.17605/OSF.IO/EFP4X ( West *et al.*, 2020) This project contains the following extended data: The list of systematic reviews and abstracts used in Step 3 to refine the ontology by applying it to relevant literature; https://osf.io/hsvp4 Expert feedback survey; Full survey provided to behaviour science experts in the review in Step 4; https://osf.io/dcmq4 The details of the method to identify papers to annotate behaviours with the Human Behaviour Ontology in Step 5; https://osf.io/csw65 Papers used in development of the Human Behaviour Ontology in Step 5 to test inter-rater reliability using the ontology; https://osf.io/rdw9f The details of the method to identify measurement scales to annotate behaviour attributes in Step 6; https://osf.io/n7vhb The classes hierarchically organised in the initial version of the Human Behaviour Ontology in Step 2; https://osf.io/v869m Record of the allocation of example behaviours that were extracted from the literature in Step 3; https://osf.io/7scz6 The classes hierarchically organised in the Human Behaviour Ontology at the end of Step 3; https://osf.io/v9ymr The classes hierarchically organised in the Human Behaviour Ontology at the end of Step 4; https://osf.io/awgtp Inter-rater reliability testing for annotations by researchers familiar with the Human Behaviour Ontology; https://osf.io/95rbx The issues recorded by researchers familiar with the Human Behaviour Ontology when applying it to annotate behaviours in interventions reports in Step 5 and responses to these issues; https://osf.io/yrnph Inter-rater reliability testing for annotations by researchers unfamiliar with the Human Behaviour Ontology; https://osf.io/ktfys The classes hierarchically organised in the Human Behaviour Ontology at the end of Step 5; https://osf.io/rxcuf Log of changes made to upper-level classes in the Human Behaviour Ontology in Step 6; https://osf.io/2wdsa Initial list of behaviour attributes generated in Step 6; https://osf.io/dmu52 The issues recorded when applying the behaviour attributes to annotate measures of behaviour in Step 6 and responses to these issues; https://osf.io/945nb Coding guidelines; Manual for coding using the Human Behaviour Ontology; https://osf.io/6e2c7 The first complete version of the Human Behaviour Ontology; https://osf.io/dc7jm/ OSF page for the Human Behaviour-Change Project; Homepage for all outputs across the project; https://osf.io/h4sdy/ Zenodo: HumanBehaviourChangeProject/ontologies: HumanBehaviourChangeProject/ontologies: Upper-Level, Setting, Mode of Delivery & Source ontologies. https://zenodo.org/doi/10.5281/zenodo.3824322 ( Hastings *et al.*, 2021) Data are available under the terms of the Creative Commons Attribution 4.0 International license (CC-BY 4.0). Source code used to calculate alpha for IRR available from: https://github.com/HumanBehaviourChangeProject/Automation-InterRater-Reliability. Archived code at time of publication: https://doi.org/10.5281/zenodo.3833816 ( Finnerty & Moore, 2020) License: GNU General Public License v3.0 only
