# An ontological framework for organising and describing behaviours: The Human Behaviour Ontology

**DOI:** 10.12688/wellcomeopenres.21252.2

**Published:** 2025-06-30

**Authors:** Paulina M. Schenk, Robert West, Oscar Castro, Emily Hayes, Janna Hastings, Marie Johnston, Marta M. Marques, Elizabeth Corker, Alison J. Wright, Gabriella Stuart, Lisa Zhang, Micaela Santilli, Susan Michie

**Affiliations:** 1 Centre for Behaviour Change, University College London, London, England, UK; 2 Department of Behavioural Science and Health, University College London, London, England, UK; 3 Future Health Technologies, Singapore-ETH Centre, Campus for Research Excellence and Technological Enterprise (CREATE), Singapore, Singapore; 4 Institute for Implementation Science in Health Care, University of Zurich, Zurich, Switzerland; 5 School of Medicine, University of St. Gallen, St. Gallen, Switzerland; 6 Swiss Institute of Bioinformatics, Lausanne, Switzerland; 7 Aberdeen Health Psychology Group, University of Aberdeen, Aberdeen, Scotland, UK; 8 Comprehensive Health Research Centre, National School of Public Health, NOVA University of Lisbon, Lisbon, Portugal; 9 Clinical and Applied Psychology Unit, Department of Psychology, The University of Sheffield, Sheffield, England, UK; 10 Rotherham Doncaster and South Humber NHS Foundation Trust, Doncaster, UK; 11 Institute of Pharmaceutical Science, King's College London, London, England, UK

**Keywords:** behaviour, behavior, human, ontology, categorisation, classification, framework, machine learning, artificial intelligence

## Abstract

**Background:**

Human behaviours have been classified in domains such as health, occupation and sustainability. We aimed to develop a broadly applicable behavioural framework to facilitate integrating evidence across domains.

**Methods:**

The Human Behaviour Ontology (HBO), a part of the Behaviour Change Intervention Ontology (BCIO), was developed by: (1) specifying its scope, (2) identifying candidate classes from existing classifications, (3) refining it by annotating behaviours in relevant literature, (4) a stakeholder review with behavioural and ontology experts, (5) testing the inter-rater reliability of its use in annotating research reports, (6) refining classes and their relations, (7) reviewing its coverage of behaviours in theories and (8) publishing its computer-readable version.

**Results:**

The initial ontology contained 128 classes (Steps 1–4), achieving an inter-rater reliability of 0.63 for familiar researchers and 0.74 after minor adjustments (to the ontology and guidance) for unfamiliar researchers. Following Steps 6–7, the published ontology included 230 classes, with six upper-level behavioural classes: human behaviour, individual human behaviour, individual human behaviour pattern, individual human behaviour change, population behaviour and population behaviour pattern. ‘Individual human behaviour’ was defined as “ *a bodily process of a human that involves co-ordinated contraction of striated muscles controlled by the brain*”, with its 159 subclasses organised across high-level classes relating to: experiences (e.g., playing); expression (e.g., laughing); reflectiveness; harm (e.g., self-injury behaviour); harm prevention; coping; domestic activities; goals; habits; health (e.g., undergoing vaccination); life-function (e.g., breathing behaviour); interactions with materials (e.g., consumption); bodily care (e.g., washing); position (e.g., postural behaviour); social environments (e.g., communication); and behavioural substitution. Additional classes needed for characterising behaviours (e.g., frequency and duration), their attributes and behavioural abstinence were included. Relations were defined for timings, locations, participants, mental processes, functions, goals and outcomes.

**Conclusions:**

The HBO provides an extensive and detailed framework for describing human behaviours.

## Plain Language summary

Human behaviours have been classified in many different ways. Having a unifying framework for describing behaviours could help us describe behaviours across different scientific domains more consistently and so better integrate the findings of different research studies. However, current classification systems for behaviour are limited in their scope, for example only focusing on behaviours that affect health. The current study aimed to develop a classification system called an ‘ontology’, to specify human behaviours extensively and coherently. Ontologies include classes (representations of anything that exists in the world, such as objects or processes) on a specific topic, with labels and definitions, and specified relations between classes. The Human Behaviour Ontology was developed by: (1) defining human behaviour and so specifying what the ontology should cover, (2) identifying classes for different types of human behaviours, (3) trying out the ontology’s classes by applying them to code examples of behaviours, and updating the ontology to amend any problematic classes, (4) seeking feedback on the ontology from behavioural science and ontology experts and refining it accordingly, (5) testing whether researchers can consistently apply the ontology, (6) finalising the classes of the ontology and specifying how they are related and (7) making the ontology computer-readable and available online. As a result, an ontology with 177 classes was developed, with 128 classes focused on ‘individual human behaviour’ and its subclasses (e.g., ‘personal bodily care behaviour’). Other classes included ones to represent repeated behaviours, behavioural attributes (e.g., whether a behaviour is done intentionally), behaviours done by groups of people as well as to describe when, where, with who/what, with what outcome and for what purpose behaviours might occur. This ontology could enable clearer and more consistent communication about behaviours and the study of behaviours, and facilitate more effective integration of research findings about human behaviour.

## Introduction

Human behaviour plays a central role in creating and solving problems for humankind ( Van Bavel *et al.*, 2020; Hulscher *et al.*, 2010; Klotz *et al.*, 2019; Marteau, 2017). Human wellbeing and life expectancy at the individual and population level is affected by behaviours, for example tobacco and alcohol use, physical exercise, dietary behaviours, harmful interpersonal behaviours and/or infection control behaviours ( Katzmarzyk & Lee, 2012; Mehta & Myrskylä, 2017). Therefore, the solution to many societal, health and environmental challenges lies in our ability to understand, predict and ultimately influence human behaviour ( Ashiru-Oredope & Hopkins, 2015; Ghebreyesus, 2021; Gifford *et al.*, 2011). Considerable advances have been made in understanding the causal pathways (e.g., through motivation, environment and abilities) that lead to specific behaviours (e.g., Bauman *et al.*, 2012; Michael *et al.*, 2014), as well as how our genetic make-up interacts with our experiences to generate behaviours (e.g., Maes *et al.*, 2004).

Many classification frameworks have been developed to describe, report and synthesise evidence about behaviours of a certain type or for a specific application (e.g., Ainsworth *et al.*, 2011; Fortune *et al.*, 2021; McEachan *et al.*, 2010). For instance, the International Classification of Health Interventions (ICHI; Fortune *et al.*, 2021) provides categories for behaviours relating to disability and health, such as ‘physical activity behaviours’ and ‘eating behaviours’. Researchers can apply this classification to consistently label and define behaviours within the specific health domain, as well as to extract and organise evidence about these behaviours. An important limitation of such frameworks is their scope. They do not attempt to represent behaviours beyond their specified domains or provide a means to describe detailed behavioural outcomes in research studies, e.g., abstinence from tobacco use for 6 months ( Baird *et al.*, 2023). Such details are important to guide clearer reporting of behaviours across studies, as well as to synthesise evidence and make systematic generalisations about behaviours.

A major challenge to creating a unifying framework is that the same behaviour can be classified in multiple ways depending on the purpose of the framework. For example, for robotics and animation purposes, it is necessary to describe behaviours in terms of physical movements and timing in order for these behaviour to be emulated accurately (e.g., Boulic *et al.*, 1990; Cully *et al.*, 2015; Magnenat-Thalmann & Thalmann, 2005). When attempting to understand and influence behaviours, such as waste recycling, tobacco use and shopping, there is a need to go beyond the purely physical description and characterise behaviours in terms of their functions, goals, outcomes, or interactions with other people or the physical environment (e.g., Chen *et al.*, 2021; Gkoutos *et al.*, 2012; Large, 2013; McEachan *et al.*, 2010). Walking, for example, may be described as a behaviour related to recreation, locomotion, protest (in the case of marches) or health promotion ( Aarts *et al.*, 1997; Büschges *et al.*, 2008; Yang & Diez-Roux, 2017). To complicate matters further, behaviours are also often classified in terms of their social meaning or external context ( Gkoutos *et al.*, 2012), for example, referring to ‘prosocial’ and ‘antisocial’ behaviours (e.g., Malti & Krettenauer, 2013). There is also divergence in what researchers mean by the term ‘behaviour’. Most definitions refer to potentially observable muscular actions, although some researchers include thoughts or secretory bodily responses ( Calhoun & El Hady, 2023; Davis *et al.*, 2015; Levitis *et al.*, 2009).

***Ontologies*** provide a potentially useful way of characterising behaviours ( Baird *et al.*, 2023; Michie *et al.*, 2017; Michie *et al.*, 2020). Ontologies represent domains of interest in terms of ***classes*** of ***entities*** (anything that exists in the universe, such as objects, ***processes*** and attributes; see glossary for bold italicised terms in Table 1) and the classes’ properties, which are specified as ***relations*** with other classes ( Arp *et al*., 2015). In other fields, such as biomedicine, ontologies have successfully served as unifying categorisation frameworks to communicate about and synthesise knowledge ( Gene Ontology Consortium, 2019).

**Table 1.** Glossary of terms.

Term	Definition	Source
**Annotation**	Process of coding, or tagging, parts of documents or data sets to identify the presence of ontology classes or items of information.	Michie *et al.* (2017)
**Annotation ** **guidance manual**	Written guidance on how to identify and tag pieces of text from intervention evaluation reports with specific codes relating to classes in the ontology, using for example EPPI-Reviewer software.	Michie *et al.* (2017)
**Basic Formal ** **Ontology (BFO)**	An upper-level ontology specifying foundational distinctions between different types of entity, such as between continuants and occurrents, developed to support integration, especially of data obtained through scientific research.	Arp *et al.* (2015)
**Class**	Classes in ontologies represent types of entities in the world. The terms ‘entity’ and ‘class’ are often used interchangeably to refer to the entities represented in an ontology. Classes can be arranged hierarchically by the specification of parent and child classes (see definition of **parent class**)	Arp *et al.* (2015)
**Entity**	Anything that exists or can be imagined, including objects, processes, and their attributes. This includes mental process, i.e., the process and content of cognitive representations, and emotions.	Arp *et al.* (2015)
**EPPI-Reviewer**	A web-based software program for managing and analysing data in all types of systematic review (meta-analysis, framework synthesis, thematic synthesis etc. It manages references, stores PDF files and facilitates qualitative and quantitative analyses. It also has a facility to annotate published papers.	Thomas, Brunton & Graziosi (2010) EPPI-Reviewer 4: http://eppi.ioe.ac.uk/eppireviewer4/ EPPI-Reviewer Web Version: https://eppi.ioe.ac.uk/eppireviewer-web/
**GitHub**	A web-based platform used as a repository for sharing code, allowing version control.	https://github.com/
**Inter-rater ** **reliability**	Statistical representation of degree of similarity and dissimilarity of coding between two or more coders. If inter-rater reliability is high this suggests that ontology class definitions and labels are being interpreted similarly by the coders.	Gwet (2014)
**Interoperability**	Two systems are interoperable to the extent that the information in one system can be used in the other system. An ontology is interoperable with another ontology if it can be used together with the other ontology, meaning they are semantically consistent.	http://www.obofoundry.org/principles/fp-010-collaboration.html
**Issue tracker**	An online log for issues identified by users accessing and using an ontology.	https://obofoundry.org/principles/fp-020-responsiveness.html BCIO Issue Tracker: https://github.com/HumanBehaviourChangeProject/ontologies/issues
**Logically defined ** **class**	A class that is defined by a logical expression or axiom specifying the conditions under which something would be included in it. Axioms – logical operators such as conjunction (AND), disjunction (OR), and negation (NOT) – can be used to write logical expressions to define a class. For instance, evaluative belief about a behaviour can be defined as “evaluative belief AND belief about behaviour”, meaning that this class captures only entities that fall in both classes – ‘evaluative belief’ and ‘belief about behaviour’	( Meehan *et al.*, 2011)
**Sibling class**	Two or more classes are sibling classes when they are direct subclasses of the same parent class.	( Noy & McGuinness, 2001)
**Open Biological ** **and Biomedical ** **Ontology (OBO) ** **Foundry**	A collective of ontology developers that are committed to collaboration and adherence to shared principles. The mission of the OBO Foundry is to develop a family of interoperable ontologies that are both logically well-formed and scientifically accurate.	Smith *et al.* (2007) www.obofoundry.org/
**Ontology**	A standardised representational framework providing a set of classes and relations for the consistent description of data and information across disciplinary, research or sectoral community boundaries.	Arp *et al.* (2015)
**Parent class**	A class within an ontology that is hierarchically related to one or more child classes (subclasses) such that all members of the child class are also members of the parent class, and all properties of the parent class are also properties of the child class.	Arp *et al.* (2015)
**Process**	Something that takes place over time.	Arp *et al.* (2015)
**Relation**	The manner in which two classes are connected or linked.	Arp *et al.* (2015)
**ROBOT**	An automated command line tool for ontology workflows.	Jackson *et al.* (2019) http://robot.obolibrary.org
**Uniform Resource** ** Identifier (URI)**	A string of western characters that uniquely identifies a document or item of information. It is used in ontologies to identify individual classes and relations within the ontology. URIs are limited to the western alphabet; the extension of these identifiers including non- western alphabet are called Internationalised Resource Identifiers (IRI). All ontology entries should have URIs that form part of URLs.	http://www.obofoundry.org/principles/fp-003-uris.html
**Uniform Resource ** **Locator (URL)**	A type of IRI that specifies a web address for a document or locatable resource on the internet. Ontology entries should all have individual URLs so that they can easily be referenced and located.	http://www.obofoundry.org/principles/fp-003-uris.html
**Versioning**	A process that involves keeping a record of different versions of files (e.g., about ontologies). Ontologies that have been released are expected to change over time as they are developed and refined, leading to a series of different files. Consumers of ontologies must be able to specify exactly which ontology files they used to encode their data or build their applications and be able to retrieve unaltered copies of those files in perpetuity. Versioning is one of the OBO Foundry principles.	http://www.obofoundry.org/principles/fp-004-versioning.html
**Web Ontology ** **Language (OWL)**	A formal language for describing ontologies. It provides methods to model classes of ‘things’, how they relate to each other and the properties they have. OWL is designed to be interpreted by computer programs and is extensively used in the Semantic Web through a dedicated Resource Description Framework (RDF) representation of the language. These representations can be used to make inferences about ontologies (based on the relations and properties) and check them for consistency.	https://www.w3.org/TR/owl2-quick-reference/

An important feature of ontologies is that every class and type of relation between classes is given a unique ID in the form of a ***Uniform Resource Identifier (URI)*** that facilitates searches for that class and its use in automated processing of information, such as for evidence synthesis or predicting intervention outcomes ( Hastings, 2017; Hastings *et al.*, 2023; Matentzoglu *et al.*, 2018; Michie *et al.*, 2017). In addition, ontologies that adopt community-agreed best-practices can support creation of more coherent and clear classes (e.g., behaviours) that promote interoperability across different research groups, data sets and scientific domains ( Gene Ontology Consortium, 2015; http://obofoundry.org/). One such best practice is that the definition of a class should take the form of its ***parent class*** (the class just above it in the semantic hierarchy) plus features that differentiate the class of interest from the parent class and other ***sibling classes*** that share the same parent class ( Arp *et al.*, 2015; Michie *et al.*, 2019; Seppälä *et al.*, 2017). For instance, the class with the label ‘sitting’ may have a parent class ‘posture behaviour’, and so the definition would be ‘A posture behaviour in which the person’s weight is supported by their buttocks.’ Another best practice example is that ontology developers should draw on the classes in existing ontologies where possible ( http://obofoundry.org/principles/fp-010-collaboration.html) and collaborate to improve their ontologies’ ***interoperability***. Ontologies should also be maintained and updated as required ( Arp *et al.*, 2015; He *et al.*, 2018).

**Figure 1.**
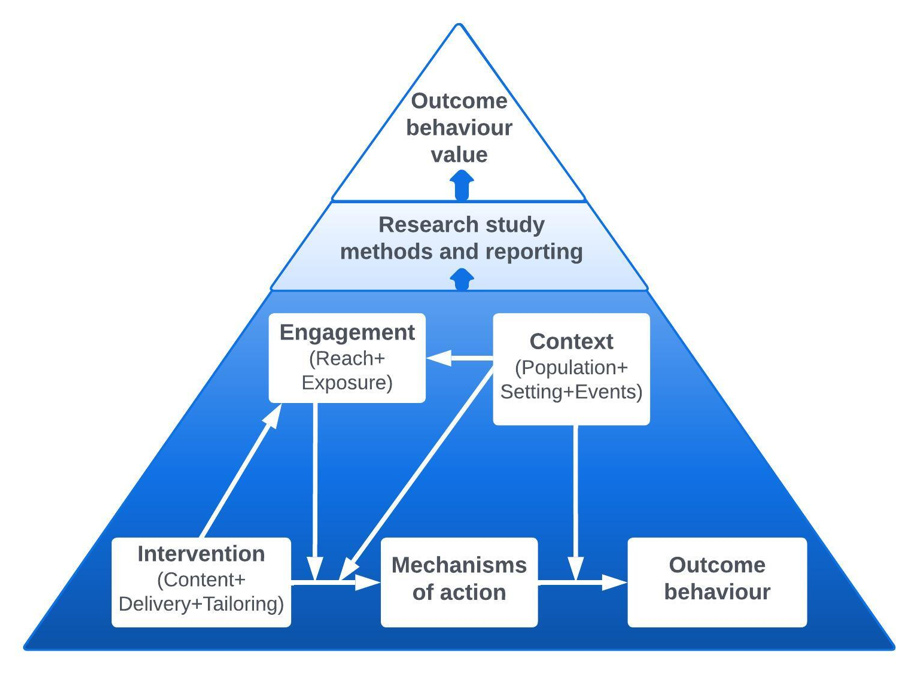
Schematic representation of the BCIO: key classes and causal connections. Source: Schenk *et al.*, 2024.

Some ontologies have been developed to capture behaviours in specific domains such as the Physical ACtivity Ontology (PACO) and COntextualised and Personalised Physical activity and Exercise Recommendations (COPPER) Ontology ( Braun *et al.*, 2023; Braun *et al*., 2025; Kim *et al.*, 2019). Other projects, such as the Tools for Understanding the Relation Between Behaviours using Ontologies (TURBBO) and the Human Behaviour-Change Project ( Michie *et al.*, 2017; Michie *et al.*, 2020), aim to represent data about behaviour more comprehensively. The TURBBO Project investigates relations between a wide range of behaviours and the application of ontologies to organise data, for example, mapping which behaviours (e.g., recycling and reducing food waste) are frequently correlated in studies ( https://sites.google.com/sheffield.ac.uk/turbbo/additional-resources#h.4k2unbgh9vh3). In parallel, the Human Behaviour-Change Project has developed the Behaviour Change Intervention Ontology (BCIO), which includes key classes about behaviour change interventions and their evaluations ( Michie *et al.*, 2017; Michie *et al.*, 2020; Wright *et al.*, 2020). One part of the BCIO is the Human Behaviour Ontology (HBO) described here, which aims to provide a framework for characterising behaviours extensively and in detail (see schematic representation of key classes in Figure 1).

## Aims

We aimed to develop an ontology (the Human Behaviour Ontology; HBO) that would (i) provide a systematic, extensive way of characterising human behaviours within behaviour change intervention scenarios, (ii) do so in a way that enabled computer readability and (iii) be expandable as required to cover all behaviours of interest to ontology users.

## Methods

The HBO was developed by drawing on the established methods of the BCIO ( Wright *et al.*, 2020). This involved an iterative process of eight steps:

1.   Specifying the scope of the ontology

2.   Identifying candidate classes and developing preliminary definitions

3.   Refining the ontology by applying it to relevant literature 

4.   Expert stakeholder review

5.   Testing ***inter-rater reliability*** of researchers applying the ontology to annotate research reports

6.   Revising classes and adding relations between classes

7.   Reviewing the ontology’s coverage by annotating constructs in behavioural theories

8.   Making the ontology machine-readable and available online

The reporting of the ontology’s development follows the Minimum Information for Reporting an Ontology (MIRO) guidelines ( Matentzoglu *et al.*, 2018).

### Step 1: Specifying the scope of the ontology

The scope of the HBO was determined by discussion within the study team, given the aim of characterising behavioural variables used in research and as targets of behavioural interventions. 

### Step 2: Identifying candidate classes and developing their preliminary definitions

To produce a set of candidate classes and definitions, we started with a top-down approach. This approach intended to capture a wide range of broad candidate classes to create a stable and broad structure of the ontology, to which more detailed classes could be added later on. To do this, we reviewed existing classification systems of human behaviour that were identified via (i) two widely used ontology repositories, Ontology Lookup Service ( www.ebi.ac.uk/ols4) and BioPortal ( https://bioportal.bioontology.org/), using the term ‘behaviour/behavior’, (ii) searches in Google Scholar using the terms ‘behaviour ontology’ and ‘behavior ontology’, and (iii) published classifications of human behaviour(s) of which the research team were aware. Table 2 shows the classification frameworks reviewed. We also searched for relevant categories in Roget’s Thesaurus ( Roget, 1997) and Medical Subject Headings (MeSH; https://meshb.nlm.nih.gov/search; Lipscomb, 2000) by using ‘behavior’ as the search term. In addition, we hand-searched the content tables of the following academic journals: Annals of Behavioral Medicine and Nature Human Behaviour. The team then discussed what additional classes might be needed to capture behaviours more fully and to create a hierarchical classification of the behaviours, using ***Basic Formal Ontology (BFO)*** as the upper-level framework ( Arp *et al.*, 2015).

**Table 2.** Classification frameworks reviewed in developing the Human Behaviour Ontology.

Classification framework	Scope of behaviours in framework	Classification structure and/or application	Ontology *	Identified in Step 2 or 3 **
A fuzzy ontology for semantic modelling and recognition of human behaviour ( Díaz Rodríguez *et al.*, 2014)	Behaviours in general but with a focus on those that can be performed in offices	A formal application of fuzzy logic to ontological modelling of human activities	Yes	Step 2
A multi-domain ontology on healthy ageing for the characterization of older adults status and behaviour ( Mastropietro *et al.*, 2023)	Functionality and social behaviours in health ageing	An application of an ontology for physiological and behavioural characterisation of older adults	Yes	Step 2
Aging neuro-behavior ontology ( Martínez-Santiago *et al.*, 2020)	Behaviours relating to disability associated with ageing	Ontology describing the cognitive processes and behaviours involved in day-to-day living and whose performance usually decline with age	Yes	Step 2
An Automatic Ontology-Based Approach to Support Logical Representation of Observable and Measurable Data for Healthy Lifestyle Management: Proof-of-Concept Study ( Chatterjee *et al.*, 2021)	Behaviours that relate to sensor measurements (e.g., fitness trackers) in support of lifestyle management eHealth interventions	An ontology for behaviours, behavioural attributes, and physiological factors related to sensor measurements	Yes	Step 2
Characteristics of health-related behaviours ( McEachan *et al.*, 2010)	Behaviours relating to health and disease	Framework focused on identifying the characteristics of behaviour that are relevant to reliably discriminate health behaviours	No	Step 2
Common-sense taxonomy of health behaviours ( Nudelman & Shiloh, 2015)	Behaviours relating to health and disease	Taxonomy of health behaviours	No	Step 2
Compendium of Physical Activities ( Ainsworth *et al.*, 2011)	Behaviours relating to physical exercise	The Compendium provides a coding scheme linking categories and types of physical activity with their respective Metabolic Equivalent of Task (MET) intensity values	No	Step 2
Development and Validation of a Functional Behavioural Assessment Ontology to Support Behavioural Health Interventions ( Merlo *et al.*, 2018)	Behaviours that are relevant for the assessment of and management of problem behaviours	An ontology for variables and behaviours relating to functional behavioural assessment	Yes	Step 2
The International Classification of Health Interventions (ICHI) ( Fortune *et al.*, 2021)	Behaviours relating to disability, health and disease	The International Classification of Health Interventions (ICHI) is part of the WHO Family of International Classifications (e.g., ICF, ICD) and aims to provide a common language and structure for describing and capturing information about health promotion interventions	No	Step 2
NCI Thesaurus OBO Edition ( Balhoff *et al.*, 2017)	Behaviours relating to cancer	Reference terminology that includes broad coverage of the cancer domain	No	Step 2
PCLiON: An Ontology for Data Standardization and Sharing of Prostate Cancer Associated Lifestyles ( Chen *et al.*, 2021)	Behaviours relating to lifestyle risk factors in prostate cancer	Ontology describing specific behaviours of relevance to cancer	Yes	Step 2
Seven characteristics of living things ( Large, 2013)	Behaviours relating to survival and reproduction	High level classification of behaviour according to function	No	Step 2
The ‘Neurobehaviour ontology’ ( Gkoutos *et al.*, 2012)	Behaviours that are shared with animals (e.g., sexual behaviours, aggression, fear- related behaviours)	An ontology of behaviours developed within the biomedical domain for annotating the behaviour of animals. Includes several behaviours of relevance for humans too	Yes	Step 2
Addiction Ontology (AddictO) ( Hastings *et al.*, 2020)	Behaviours relating to addiction and its management or prevention	Ontology representing all of the constructs that researchers, practitioners and policy makers want to refer to in the field of addiction.	Yes	Step 3
ICD-11 ( Almeida *et al.*, 2020)	Behaviours relating to disability, health and disease	The International Classification of Diseases and Related Health Problems (ICD) aims to provide a global standard for coding health information and causes of death	No	Step 3
ICF-Behave V.1.0 ( Larsen *et al.*, 2021)	Behaviours relating to disability, health and disease	Taxonomy of behaviours in the context of the WHO international classification of functioning disability and health (ICF)	No	Step 3
Physical ACtivity Ontology (PACO) ( Kim *et al.*, 2019)	Behaviours relating to physical exercise	Ontology developed to support structuring and standardising heterogeneous descriptions of physical activities	Yes	Step 3
Townsville Residential Energy Demand (TRED) program behaviours	Behaviours relating to sustainability	List of behaviours that lead to reduction in home energy consumption identified through literature search and expert panel	No	Step 3

*A classification framework was considered to be an ontology when their authors referred to it as such and the classification system was available in Web Ontology Language (OWL). **Two searches were run to identify classification frameworks, one in Step 2 and the other in Step 3

Ontological definitions for the classes were drafted based on dictionaries, existing ontological definitions and group discussions informed by guidelines for ontological definitions ( Michie *et al.*, 2019; Seppälä *et al.*, 2017). To organise these classes, preliminary hierarchical relations between parent and child classes were specified: Researchers identified broader classes which could serve as parents, and more specific classes that shared all their characteristics and so were considered their subclasses. In terms of the mechanics of ontology development, classes were originally set out in an Excel spreadsheet, with each class organised in a separate row with columns for its label, definition, parent class, informal definition, examples and relations.

### Step 3: Refining the ontology by applying it to relevant literature

We investigated whether the ontology structure was stable and comprehensive enough to allow characterisation of a wide range of behaviours through an iterative process of literature ***annotation***, team discussion, and revision. We identified examples of behaviours from three sources and assessed whether they could be annotated within the existing HBO structure. The sources were (i) five additional classification frameworks that were identified in an updated search, using the same method described in Step 2 (see Table 2), (ii) published systematic reviews of behaviour change interventions, and (iii) abstracts of studies published in journals addressing a breadth of human behaviours.

To identify the systematic reviews, a search using the terms ‘systematic review’ and ‘behaviour/behavior’ at the title level was performed in Web of Science, the Cochrane Library and ProQuest (February 2020). Fifteen reviews were included, covering a range of domains, such as environment, sexual health, education, and clinical practice. To identify abstracts, a search using the term ‘behavio*’ was performed in SCImago (a database of scientific journals) to generate a list of journals (September 2020). The 224 journals identified were divided into ‘broad’ (e.g., Nature Human Behaviour) or ‘specific’ (e.g., Journal of Consumer Behaviour). Five ‘broad’ journals were randomly selected and 20 abstracts from their last published issues (including ‘in press’ or ‘online first’) were retrieved for analysis (i.e., 100 abstracts in total). A list of these systematic reviews and abstracts can be found in: https://osf.io/hsvp4.

To test whether a behaviour found in a source could be clearly captured by classes in the ontology, two researchers (EH & OC) independently annotated mentions of behaviours (referred to as ‘example behaviours’) from these three sources, using the HBO prototype developed in Step 2. For example, from one abstract about political behaviour, the term ‘voting’ was annotated as belonging to the class ‘political behaviour’ in the HBO. When a source included synonyms for a behaviour (e.g., ‘taking medication’ and ‘taking prescribed drug’), this behaviour was annotated only once. During the annotation process, the researchers considered whether:

- Any class definitions needed to be reworded,
- The HBO structure needed to be altered,
- Any behaviours could not be captured by classes in the ontology, indicating the need for new classes.

Discrepancies in annotations were resolved by discussion between the two annotators or, if required, the whole research team. To avoid overpopulating the ontology with too many classes before having a stable structure, new classes were only added if they captured several of the extracted behaviours. For example, specific behaviours, such as toothbrushing and hair brushing, could be captured under a broader class of behaviours to take care of one’s body. These specific annotated behaviours were then included as examples in the higher-level classes to which they belonged. This was done under the principle that such examples could be added as classes in their own right once the structure of the ontology was clearly established and if users identified a need for them.

### Step 4: Expert stakeholder review

We invited 94 behaviour change experts and four ontology experts (see Michie *et al.*, 2020; Norris *et al.*, 2021) to review the ontology. Behaviour change experts were invited from a list of those who: (i) provided feedback on previous projects by the Centre for Behaviour Change at University College London and indicated willingness to be contacted for future projects, or (ii) expressed interest in being involved in the Human Behaviour-Change Project stakeholder initiatives. To be eligible, these experts needed to have a doctoral degree in behavioural science or a related discipline. Experts from both 'well-represented' countries (UK, USA, Canada, Australia, the Netherlands) and 'less-represented' countries (e.g., Chile and France) were randomly selected to provide feedback. Ontology experts were suggested by JH, the Human Behaviour-Change Project’s ontology expert. Recruitment continued until at least 10 participants completed the stakeholder review; participation in the study was completely voluntary and not remunerated. For this stakeholder review, ethical approval was granted by University College London’s Research Ethics Committee (CEHP/2020/579) in February 2020. Participants provided informed written consent via an online Qualtrics survey before starting the review.

To support participants familiarising themselves with the HBO and the review task, the experts watched three introductory videos explaining what ontologies are, with a focus on the HBO ( https://vimeo.com/721051844; https://vimeo.com/726324041; https://vimeo.com/726779845). Feedback on the HBO was collected through an online questionnaire, using Qualtrics ^TM^ software ( https://www.qualtrics.com; see complete survey here: https://osf.io/dcmq4). Participants were presented with class labels, definitions, parent classes and, where relevant, their informal definitions and examples. Participants were asked to provide feedback through open-ended questions on:

- Clarity: whether the labels and definitions could be understood by experts who did not develop the HBO,
- Representativeness: whether the ontology comprehensively covered constructs of interest, i.e., whether any classes were missing, and
- Structure: whether any classes need to be reorganised in the ontology’s structure.

The research team extracted and logged each piece of participant feedback from Qualtrics ( https://www.qualtrics.com/uk/). The team then discussed how to address each issue raised, recording decisions to revise the ontology based on feedback or the rationale for not making revisions. The HBO was updated based on the discussions.

### Step 5: Testing inter-rater reliability of researchers applying the ontology to annotate research reports

To investigate whether the HBO could be reliably used to classify behaviours, we evaluated the inter-rater reliability (IRR) of researchers applying the updated ontology to annotate (i.e., code) mention of behaviours. In line with the methods for developing other lower-level ontologies of the BCIO ( Wright *et al*., 2020), we annotated these behaviours in 100 papers on the web-based software, ***EPPI-Reviewer*** v4 ( Thomas *et al.*, 2010; Thomas *et al.*, 2020). An open access alternative for this annotation software is PDFAnno ( Shindo *et al.*, 2018). 

Relevant papers were identified by running a search on the database Open Alex ( Priem *et al.*, 2022) in September 2022. This database was released in 2022 and presents an open access alternative to other commonly used academic databases with a wide range of publications (with metadata for 209 million works, including journal articles and books) ( Price, 2024). In the search, we identified papers that:

(1) cited either the Theoretical Domains Framework ( Cane *et al.*, 2012), a framework synthesising broad influences on behaviour, or the Behaviour Change Techniques Taxonomy v1 (BCTTv1; Michie *et al.*, 2013) These two papers were used as starting point, as they have been a widely cited and used in protocols, intervention development and evaluations related to behaviours ( Birken *et al.*, 2017; Corker *et al.*, 2023). The search identified papers that cited the TDF or BCTTv1 in any part of the paper, meaning that these frameworks did not need to be used in the study.
(2) were published 2 years before the search (2020–2022), assuming that recent papers were more likely to include clearer descriptions of behaviour.

After selecting papers published within 2020 to 2022 and removing duplicates, 2532 papers were identified. For the ontology to be applicable to a wide range of different behaviours, we aimed to identify papers that fit into the following criteria:

- Mention of specific human behaviour, and
- Report this behaviour within the context of (i) a study investigating or exploring behaviour, (ii) an intervention development or evaluation process, or (iii) behavioural model, theory or framework.
- Not be a book chapter, a systematic, scoping or literature review or a thesis or dissertation.

Sets of 30–50 papers’ titles and abstracts, and then full texts (for papers whose inclusion could not be assessed based on the title and abstract alone) were screened against the inclusion criteria, until at least 100 eligible papers were identified. Additional details about the method for identifying suitable papers to annotate can be found in: https://osf.io/csw65. Altogether, 110 eligible papers were identified, of which 100 were randomly selected for annotation. The full list of papers annotated can be found in: https://osf.io/rdw9f.

IRR was assessed in two stages. First, 50 papers were independently annotated by two researchers involved in the development of the ontology (PS & GS) on EPPI-Reviewer v4 ( Thomas *et al.*, 2010; Thomas *et al.*, 2020). Fifty papers were selected to provide a 10–15% margin of error around the estimated inter-rater agreement value ( Gwet, 2014). Second, two behavioural science researchers unfamiliar with the ontology but with annotation experience (LZ & MS) annotated the remaining 50 papers. IRR was calculated with Krippendorff's alpha ( Hayes & Krippendorff, 2007) using Python 3.6 ( Finnerty & Moore, 2020).

Alpha values above 0.67 indicate acceptable IRR ( Krippendorff, 2009; Krippendorff, 2011), with values below this threshold indicating that the class labels and definitions are likely to be interpreted differently by researchers. If the IRR was lower than 0.67 in an annotation stage, the two annotators reviewed their annotation disagreements across 50 reports and suggested changes to the ontology or its ***annotation guidance manual***. This also meant that if the IRR in Stage 1 fell below the threshold, the ontology and its annotation guidance were revised before proceeding to annotations in Stage 2. The wider research team provided feedback on these suggestions, and updates were made to the ontology or annotation guidance accordingly.

### Step 6: Revising classes and adding relations between classes

To structure the classes in the HBO, the research team formally specified hierarchical relations between classes. For such relations, a principle is to ensure that each class has only one parent class. While it is not a strict requirement in ontologies to have a single parent class, it is a recommendation ( Arp *et al.*, 2015), and we adopted the recommendation for the BCIO as far as possible because it makes the ontology easier to maintain and browse. When selecting parent classes for a class in the HBO, behaviours were specified in terms of a behavioural class to which a behaviour *always* belongs, although it may sometimes belong to other classes. For example, ‘walking’ is *sometimes* a ‘travel behaviour’, but in other cases (e.g., walking on a treadmill) walking will not involve travelling. Instead, walking is *always* a ‘locomotive behaviour’ (as walking always involves the use of muscles to move relative to the immediate environment or as part of it). Therefore, ‘locomotive behaviour’ would be selected as the parent class of ‘walking’ in the HBO.

Additional relations needed to be specified for future use of the ontology, as a hierarchical classification system on its own would not be adequate to characterise the way that behaviours are described in the research literature. The same class of behaviour (e.g., ‘walking’) can be used to characterise different types of behaviour. For instance, walking on a treadmill could be done purely for physical exercise, whereas walking to a store would be a transportation behaviour. In the ontology, one way of characterising behaviours more precisely is by defining relations between classes, including behavioural attributes. From combinations of these relations, ***logically defined classes*** can be created where needed. For example, different types of walking behaviour can be specified more precisely through logical definitions: ‘walking’ that *occurs in* some ‘park’. In this example, *occurs in* is a defined relation that can link a behaviour to a range of contexts in the Intervention Setting Ontology within BCIO ( Norris *et al.*, 2020). Therefore, the research team decided to include a set of relations that can be specified between classes in the ontology.

It is important to note that these relations were created as a starting point to allow ontology users to characterise behaviours with more detail but specifying relations between granular classes (e.g., ‘physical performance behaviour’ and its relevant goals) was beyond the scope of the current work. In accordance with best practice ( Arp *et al.*, 2015), the relations were imported where possible from Basic Formal Ontology (BFO) and the Relations Ontology (RO) (which sits under BFO) ( Smith *et al.*, 2005). Where new relations had to be defined, they were specified as subclasses of relations in BFO or RO.

We also proposed an initial list of ‘behavioural attributes’ by drawing on the ‘Big Question’ addressed by the Human Behaviour-Change Project ( www.humanbehaviourchange.org): “What works, compared with what, for what behaviours, how well, for how long, with whom, in what setting, and why?” ( Michie *et al.*, 2017). This list was also informed by published characteristics of health-related behaviours ( McEachan *et al.*, 2010) and published ‘applied behavioural analysis’ literature ( Cooper *et al.*, 2020). Behavioural attributes were broadly defined as characteristics of a behaviour that help specify it. This definition was purposefully kept broad to identify various details that should be specified about a behaviour and thereby be included in the HBO.

To refine the behavioural attributes, two researchers independently applied these attributes to annotate 50 scales measuring some aspect of behaviour (see details of method to identify measurement scales in: https://osf.io/n7vhb). The researchers compared their annotations and discussed discrepancies, raising issues with the wider research team where necessary. These discussions informed changes to the behavioural attributes, e.g., labels and definitions were updated, or behavioural attributes were added. The research team then discussed how each attribute should be formally captured in the ontology, either as a new class and/or a relation between a behaviour and some other class. Ontological labels and definitions were written for these classes and relations.

To improve the structure and navigability of the ontology, we reviewed the upper-level classes to identify overarching parent classes. Where needed, new overarching classes were developed, labelled, defined and specified as parent classes of relevant classes.

### Step 7: Reviewing the ontology’s coverage by annotating constructs in behavioural theories

The HBO’s coverage was explored by applying it to annotate theoretical constructs defined as behaviours and relevant variables. This was part of an effort to map the BCIO to 1516 constructs in 76 theories of behaviour and behaviour change (Michie et al., in prep). One of three researchers (MS, MB & PS) annotated the constructs with the relevant class from the BCIO, including the HBO. These annotations were reviewed by a team of four or more researchers (JH, MB, SM, RW, PS, and/or LZ), who discussed any points of uncertainty or disagreement. When a new class was needed to adequately represent a behavioural construct, a researcher proposed a label and definition for the class and any relevant structural classes (e.g., parents). These were reviewed by the wider team before being incorporated into the HBO.

### Step 8: Making the ontology machine-readable and available online

When the HBO content was ready for its initial release, it was converted from the spreadsheet format into ***Web Ontology Language (OWL)*** ( Antoniou & van Harmelen, 2004) format, using a custom script which uses the ***ROBOT*** ontology toolkit library ( Jackson *et al.*, 2019). The OWL format is a standard format, thereby allowing the ontology to be compatible with other ontologies, and viewed and visualised on ontology software, such as Protégé ( Musen, 2015). A ROBOT template is a comma-separated values (CSV) file that can be prepared using spreadsheet software (e.g., Excel) for conversion from spreadsheet columns into OWL language and metadata attributes. In the input template spreadsheet, we used separate columns to capture the unique semantic-free class IDs (e.g., BCIO:01023), labels, definitions, parent class, informal definitions, relations with other classes, comments, examples and synonyms. The OWL version of the HBO is stored on the Human Behaviour Change Project’s project ***GitHub*** repository ( https://github.com/HumanBehaviourChangeProject). The GitHub repository supports ***versioning*** the ontologies and has an ***issue tracker***, which enables ontology users to provide feedback on issues to be addressed in future releases of the ontology ( https://github.com/HumanBehaviourChangeProject/ontologies/issues). Finding and examining individual classes in the ontology was made possible by building a bespoke website BCIOSearch ( https://BCIOsearch.org). Visualising the hierarchical relations between classes was made possible by building the bespoke BCIOVisualise website ( https://bciovis.hbcptools.org/). These tools are available via the BCIO website ( https://www.bciontology.org/).

## Results

### Step 1: Specifying the scope of the ontology

The scope of the HBO was the full range of potentially observable human behaviours investigated in the research literature, which allows any behaviour targeted by an intervention to be included in this ontology. This includes partially overlapping behavioural domains relating to immediate survival, hygiene, longer-term health, mental wellbeing, reproduction, transport, teaching and learning, economic activity, social activity, professional activity, environmental sustainability, social interaction, crime, violence, security, leisure, play, religion, culture and the arts.

Building on the definition of behaviour previously generated based on feedback from 24 behavioural science experts ( Davis *et al.*, 2015), the ontological definition of a class labelled ‘individual human behaviour’ was:

*“A bodily process of a human that involves co-ordinated contraction of striated muscles controlled by the brain.”*

The scope of the HBO included individual human behaviour (as defined), its subclasses, anything required to characterise these classes, as well as how individual behaviours combine (e.g., patterns of behaviours) or scale up (e.g. population-level behaviours). 

We excluded beliefs, intentions, decisions, perceptions and feelings. These were classified in the BCIO as mental processes, cognitive representations and dispositions rather than behaviours (see the Mechanism of Action Ontology within BCIO; Schenk *et al.*, 2024). Also excluded from the ontology were physical processes undertaken by bodies that: are not controlled by the brain (although they might be influenced by it) (e.g., spinal reflexes); do not involve striated (voluntary) muscles (e.g., peristalsis); or involve the activities of glands (e.g., sweating, salivating). Thus, for example ‘sleep’ was not in scope but behaviours related to sleep such as ‘going to bed’ and ‘lying down’ fell within its scope.

The ontology developers also recognised that a distinction needed to be made between behaviours of individuals and of populations in the HBO. Ontologically these are different things. Individual occurrences of a behaviour are single processes whereas population behaviours are multiple processes. Population behaviours are characterised in terms of incidence and prevalence (e.g., cigarette smoking prevalence). Therefore, to capture human behaviours, both on an individual and population level, a more general class was needed. In the ontology, ‘human behaviour’ was defined as “ *A process that is an individual human behaviour or a population behaviour*” (see Step 6 for the formal addition of this class). Its subclass ‘behaviour change intervention outcome behaviour’ (BCIO: 002000) was defined as “ *Human behaviour that is an intervention outcome.”*

In principle, any specific occurrence of a behaviour would have a parallel population and intervention outcome behaviour version. To avoid tripling the number of classes, it was decided to specify all the classes at the level of individual occurrences and focus on the subclasses of ‘individual human behaviour’. Due to this focus, the classes for ‘population behaviour’, ‘human behaviour’ and ‘behaviour change intervention outcome behaviour’ were noted down for formal inclusion in the ontology when structuring it in Step 6. However, it should be noted that classes in the HBO can be used to capture different outcome behaviours by double annotating the ‘behaviour change intervention outcome behaviour’ class and the relevant subclass of individual human behaviour (e.g., ‘walking’).

### Step 2: Identifying key classes and developing their preliminary definitions

The preliminary ontology included 85 classes, with 61 classes relating to individual human behaviours and 24 classes related to functions that may be realised by some of these behaviours (e.g., life maintenance, life enhancement, environment management, learning, social integration). Each class was connected to its parent class with an ‘is_a’ relation ( Smith *et al.*, 2005), e.g., ‘alcohol consumption’ *is_a* ‘consumption behaviour’. The 85 classes were organised on five hierarchical levels, and behaviours were organised at this step within 11 upper-level classes: functional, locomotive, posture, sexual, expressive, physical impact, grooming, goal-oriented, interpersonal, socially-evaluated, and object-involving behaviours (see these classes in: https://osf.io/v869m).

### Step 3: Refining the ontology by applying it to relevant literature

Based on the 334 example behaviours identified in the research literature, the labels and/or definitions of 51 classes were updated. In addition, 35 classes were added to the ontology. Most of these classes were broad enough to capture more specific examples of behaviour. However, some of these were added due to the need to distinguish between single occurrences of a behaviour and repeated occurrences of the same behaviour (behaviour patterns). While an ‘individual human behaviour’ is a single occurrence, behaviour patterns are multiple processes distributed over time. Behaviour patterns have characteristics such as frequency and temporal patterning (e.g., daily cigarette consumption). To avoid adding too much detail to the ontology, broad classes for behaviour patterns were specified (e.g., ‘behaviour pattern’) and subclasses for tobacco-related behaviour pattern classes were created as an example use case. Similarly, to capture changes in a behavioural occurrence or pattern, the class ‘individual human behaviour change’ and, as example use case, its subclasses for tobacco-related behavioural changes were added.

Of the 334 example behaviours, 217 could be captured under an existing class in the ontology and so were added as examples of these classes. For example, ‘urinating’ was added as an example of ‘excretion function behaviour’. A further 27 behaviours were allocated as examples for eight new classes in the ontology (e.g., ‘voting’ was added as an example of ‘political behaviour’). The remaining behaviours could not be allocated because they: (i) were too generic (e.g., ‘planning’; 23 behaviours), (ii) did not fit into the current definition of behaviour (e.g., ‘psychomotor agitation’; 18 behaviours), (iii) were duplicates (i.e., the class already existed in the ontology; 16 behaviours), (iv) were a grouping of behaviours rather than a single behaviour (e.g., ‘activities of daily living’; 13 behaviours), or (v) were too specific to be useful examples and were otherwise captured in the ontology (e.g., ‘switching on’; 6 behaviours) (see details of the behaviours that were recorded as examples or not allocated in: https://osf.io/7scz6).

At the end of this step, the ontology consisted of 120 classes across eight hierarchical levels (see classes in: https://osf.io/v9ymr).

### Step 4: Expert stakeholder review

Ten experts (eight behavioural scientists and two ontology experts) agreed to participate in the review and met the eligibility criteria. The two ontology experts’ primary domain was in biomedicine, where ontologies are more widespread. The behavioural experts worked in domains relating to health behaviours (e.g., treatment adherence, physical activity, dietary behaviours, developmental, parenting and care-taker behaviours, communication behaviours, and behaviours relating to disaster management). The majority had expertise in more than one behavioural domain. These experts worked in institutions based in: Australia (n = 1), Chile (n = 1), France (n = 1), Ireland (n = 1), United Kingdom (n = 2) and United States (n = 4).

Participant comments and the steps taken to address each comment or the rationale for not addressing specific comments were recorded (see this record in: https://osf.io/r8g6a). These comments were about the ontology’s overall structure, specific issues with class labels and definitions, suggestions to divide classes into subcomponents and classes missing from the ontology. Examples are:

- In relation to the ontology’s overall structure, a participant suggested considering a polyhierarchical structure (i.e., specifying multiple parent classes for a class). However, we retained the principle of only having one parent for each class because multiple inheritance in ontologies can lead to inconsistencies, ambiguities and semantic conflicts when different parent classes impose contradictory constraints or properties on the child class.
- On the class, ‘physical impact behaviour’, a participant commented that the class definition (“An individual human behaviour that makes forceful physical contact with something.”) did not capture ‘touch’, which was presented as an example of this class. The participant highlighted that touch did not involve forceful physical contact and suggested that a more general class for physical contact behaviour could better capture the example and serve as a parent to the class ‘physical impact behaviour’. In response, we added ‘physical contact behaviour’ as a class and touch as an example, and organised ‘physical impact behaviour’ as its subclass.

In response to all the comments, 25 class labels, 42 class definitions and the parent classes of 26 classes were updated. For 19 classes, examples were added or changed; for 23, comments were added or changed; for five, informal definitions were added.

Altogether, 51 classes were removed from the ontology, as it was considered that they were underspecified or overcomplicated the ontology. For example, some stakeholders pointed out a strong dependency between the definitions of functions and their corresponding functional behaviours. They suggested that this dependency made the definitions of functional behaviours circular and unclear. In response to these comments, we decided against the formal use of specific functions (e.g., ‘life maintenance function’) to specify the definitions of relevant behavioural classes (e.g. ‘life maintenance function behaviour’). We removed the detailed subclasses of ‘human life function’ from the ontology, only keeping the high-level classes for function (‘function’, ‘animal life function’ and ‘human life function’) to allow users to specify specific functions of behaviours when needed. Function-related behaviours were also kept as classes, if they were not captured by other behavioural classes, and their definitions were updated to reflect the ontology’s structural change.

The classes ‘outcome behaviour’ and ‘goal-related behaviour’ were also removed from the ontology as they were considered confusing by experts. For example, one participant stated that “ *I am totally confused about what an 'outcome' is in this definition... I would not know what behaviours belong to this group*”. Another participant wrote about goal-oriented behaviour: “ *Is “Goal-oriented behaviour” a class? This can only be assumed. This would be better as an attribute of a behaviour.”* While these classes were removed from the ontology in this step, the potential relations ‘has behavioural goal’ and ‘has behavioural outcome’ were recorded for Step 6. It should be noted that classes can be re-added to the ontology in the future should there be the need for them.

Finally, to address participants’ comments regarding missing classes, 59 classes (e.g., ‘physical contact behaviour’) were added. These changes resulted in an ontology with 128 classes, including 125 behavioural classes and 3 classes to capture functions on a high level (see updated version of the ontology in: https://osf.io/awgtp).

### Step 5: Testing inter-rater reliability of researchers applying the ontology to annotate research reports

In this step, only the ontology’s 125 behavioural classes were used to investigate the inter-rater reliability for annotating examples of behaviour, as the three high-level functional classes were not useful for annotating specific behaviours. The inter-rater reliability for two researchers familiar with the ontology was 0.63 which was marginally lower than the set threshold of 0.67 (see https://osf.io/95rbx). Annotation discrepancies were examined, and changes to the ontology and the ***annotation guidance manual*** were made (see details: https://osf.io/yrnph). Most changes were made to the annotation guidance, but examples were added or changed for three classes, and a synonym was added to one class. Four classes (e.g., ‘alcohol consumption’) were added to the ontology.

Using the updated version of the ontology and annotation guidance, two researchers unfamiliar with the ontology had an acceptable inter-rater reliability (α = 0.74) (see https://osf.io/ktfys). The ontology at this stage had 129 behavioural classes, with 34 serving as upper-level classes such as ‘antisocial behaviour’, ‘economic behaviour’, ‘environmental system management behaviour’, ‘personal bodily care behaviour’ and ‘providing healthcare’. Including the three function-related classes, the ontology had 132 individual human behaviour classes in total at the end of this step (see https://osf.io/rxcuf).

### Step 6: Revising classes and adding relations between classes

To structure the ontology’s highest-level, the classes for ‘population behaviour’, ‘population behavioural pattern’ and ‘human behaviour’ (a logical class to capture any human behaviour, including individual and population behaviours) were formally added. The class ‘behaviour change intervention outcome behaviour’ was also added and organised under ‘human behaviour’ to explicitly capture the individual and population outcome behaviours in an intervention (also see Step 1). Along with the high-level classes for individual human behaviour, their patterns and change (see the process for adding these to the ontology in Step 3), this resulted in seven high-level behavioural classes. To link the HBO to the wider BCIO, the class for behavioural intervention outcome was also formally linked to ‘behaviour change intervention scenario’ in the Upper-level BCIO ( Michie *et al.*, 2020): ‘behaviour change intervention scenario’ *has_process_part* ‘behaviour change intervention outcome behaviour’. The labels and definitions of these classes are presented in Table 3.

**Table 3.** Upper-level behavioural classes in the HBO.

Label	Definition	Parent class
individual human behaviour *BCIO:036000*	A <bodily process> of a human that involves co- ordinated contraction of striated muscles controlled by the brain.	bodily process
individual human behaviour pattern *BCIO:036100*	A uniform process aggregate whose member parts are behaviours of the same type and in the same person.	uniform process aggregate
individual human behaviour change *BCIO:050209*	A process that results in a difference in enactment of some individual human behaviour or individual human behaviour pattern from what would have been the case otherwise.	process
population behaviour *BCIO:034000*	An aggregate of individual human behaviours of members of a population.	process aggregate
population behaviour pattern BCIO:050448	A uniform process aggregate whose members are individual behaviour patterns of a population.	uniform process aggregate
human behaviour *BCIO:042000*	A process that is an individual human behaviour or a population behaviour.	process
behaviour change intervention outcome behaviour *BCIO:002000*	Human behaviour that is an intervention outcome.	human behaviour

To align with the Mechanism of Action Ontology, part of the wider BCIO ( Schenk *et al*., 2024), two new classes (‘habitual behaviour’ and ‘normative behaviour’) were added to the HBO. With these, there were 36 upper-level classes of individual human behaviour, resulting in an unwieldy structure. It was possible to group these into just ten classes relating to: (1) experiences (e.g., enjoyment behaviour, such as playing), (2) expressive (e.g., laughing), (3) habit (e.g., toothbrushing after waking up), (4) harm (e.g., self-injury behaviour), (5) health (e.g., utilising healthcare), (6) life function (e.g., breathing behaviour), (7) interacting with materials and objects (e.g., consumption behaviour), (8) personal bodily care (e.g., bodily hygiene behaviour), (9) position and location (e.g., travel behaviour) and (10) the social environment (e.g., human communication behaviour) (see definitions in https://osf.io/cqe2w). Of the ten classes, seven were new classes added to structure the ontology. Although there is some overlap between these classes - for example consumption behaviors can also be health-related behaviors (e.g., eating vegetables) - parent-child class assignments were determined based on which parent a class would consistently belong to. In the case of consumption behaviour, they would always involve interacting with some material or object and so were recorded as a subclass of ‘material entity-related behaviour’.

Changes were made to some class definitions to specify them more clearly (see the log of changes to the upper-level classes in https://osf.io/2wdsa). In addition, six classes (e.g., ‘crying’, ‘facial expression behaviour’ and ‘vocalisation behaviour’) were added to more fully capture expressive behaviours. The class ‘impulsive behaviour’ was removed, and ‘impulsiveness’ was noted down as a potential behaviour attribute instead. The class ‘animal life function’ was also removed from the ontology, as the HBO is specific to humans. This resulted in 149 classes. The immediate subclasses of the updated upper level (developed in this step) are presented in the OSF file: https://osf.io/cqe2w

***Adding behavioural attributes and relations*.** To capture additional characteristics of behaviour that help specify them (e.g., timing or location), the research team generated an initial list of attributes (see https://osf.io/dmu52). Examples were location, duration, frequency and physical exertion required during the performance of behaviours. Two researchers applied these attributes to annotate items in measures of behaviour; annotation issues were recorded and resolved (see https://osf.io/945nb). This resulted in 18 putative behavioural attributes, with the addition of three new attributes: ‘behaviour starting timepoint’, ‘behaviour end timepoint’ and ‘behaviour target person’. Through additional reviews of the attributes, the research team:

1. Captured 22 classes based on the list of behavioural attributes to include in the ontology, and further developed nine classes (‘behaviour attribute’, ‘impulsiveness’ ‘reflectiveness’, ‘intentionality’, ‘behaviour disposition’, ‘abstinence from a behaviour’, ‘abstinence duration’, ‘abstinence start point’ and ‘abstinence end point’), thereby adding 31 classes to the ontology,
2. Represented nine relations based on the list of attributes, and further specified five relations (e.g., ‘has behavioural goal’) between behavioural classes and ontology classes lying outside of the lower-level ontology, one to specify abstinence duration (‘has abstinence duration’) and one to link ‘human function’ to a person, resulting in 16 non-hierarchical relations being specified,
3. Removed two attributes that did not capture sufficiently clear or unique aspect of behaviour.

Where no relevant relation could be identified from the Relation Ontology ( Smith *et al.*, 2005), such as for ‘has behavioural goal’ and ‘has behavioural outcome’, new relations were developed. These relations were specified to allow potential ontology users to link a behaviour to a particular external class. For instance, to capture that the outcome of behaviour is the emotion ‘happiness’ from the Emotion Ontology ( Hastings *et al.*, 2011), the following relation can be specified: ‘individual human behaviour’ *has_behavioural_outcome* ‘happiness’ ( http://purl.obolibrary.org/obo/MFOEM_000042). The outcomes and goals of behaviours can involve any entity (e.g., processes or dispositions), and therefore these relations were designed to be used flexibly by ontology users by linking to any class.

In total, the ontology had 17 relations: the ‘is a’ relation, the ‘has behavioural attribute’ relation and 15 other relations (see Table 4). In order to represent many of the behaviours of interest in behavioural science, it is necessary to create classes using logical expressions that combine classes and relations ( Köhler *et al.*, 2011). For example, a class ‘moderate physical activity’ would be defined by the expressions (‘physical performance behaviour’) and (‘has behavioural attribute’ ‘moderate physical exertion expended on a behaviour’). At the end of the step, the ontology had 180 classes.

**Table 4.** Relations in the Human Behaviour Ontology.

Label	Definition * (domain/range ^#^)	Parent class	Relation in the HBO	Informal definition
*has start time* *BCIOR:000020*	A relation between an occurrent x and a one-dimensional temporal entity such that the one-dimensional temporal entity designates the earliest boundary of x's existence or manifestation. (occurrent/occurrent)	temporally related to *RO:0002222*	individual human behaviour *starts* one-dimensional temporal region	A relation that specifies a timepoint at which a behaviour starts.
has end time *BCIOR:000019*	A relation between an occurrent x and a one-dimensional temporal entity such that the one-dimensional temporal entity designates the latest boundary of x's existence or manifestation. (occurrent/occurrent)	temporally related to *RO:0002222*	individual human behaviour *ends* one-dimensional temporal region	A relation that specifies a timepoint at which a behaviour ends.
occupies temporal region *BFO:0000155*	p occupies_temporal_region t. This is a primitive relation between an occurrent p and the temporal region t upon which the spatiotemporal region p occupies_ spatiotemporal_region projects. (occurrent/occurrent)	exists at *BFO:0000108*	individual human behaviour *occupies_temporal_region* one- dimensional temporal region	A relation that specifies the duration of a behaviour.
happens during *RO:0002092*	X happens_during Y if: (start(Y) before_ or_simultaneous_with start(X)) AND (end(X) before_or_simultaneous_with end(Y)). (occurrent/occurrent)	temporally related to *RO:0002222*	individual human behaviour *happens_during* one- dimensional temporal region	A relation that specifies that the behaviour occurs during a specified temporal interval.
has abstinence period *BCIOR:000009*	A relation that links abstinence from a behaviour to a temporal region during which this personal attribute is true. (abstinence from a behaviour/ abstinence duration)	relation	abstinence from a behaviour *has_abstinence_period* abstinence duration	A relation that specifies the duration for which a person is abstinent from a behaviour.
occurs in *BFO:0000066*	b occurs_in c =def b is a process and c is a material entity or immaterial entity & there exists a spatiotemporal region r and b occupies_spatiotemporal_region r.& for all(t) if b exists_at t then c exists_at t & there exist spatial regions s and s’ where & b spatially_projects_onto s at t& c is occupies_spatial_region s’ at t& s is a proper_continuant_part_of s’ at t (occurrent/independent continuant)	relation	individual human behaviour *occurs_in* location	A relation that specifies a place where a behaviour happens.
has behavioural attribute *BCIOR:000010*	A relation that links an individual human behaviour to a behavioural attribute. (individual human behaviour/ behavioural attribute)	relation	individual human behaviour *has_behavioural_attribute* behavioural attribute	A relation that specifies the characteristics of a behaviour, such as its frequency, the effort it requires, its form and whether it’s intentional.
serves behavioural function *BCIOR:000011*	Realises the human life function of an individual human behaviour. (individual human behaviour/human life function)	realises *BFO:0000055*	individual human behaviour *serves_behavioural_function* human life function	A relation that specifies the biological or social function of a behaviour.
is attribute of *RO:0000052*	A relation between a specifically dependent continuant (the dependent) and an independent continuant (the bearer), in which the dependent specifically depends on the bearer for its existence. (specifically dependent continuant/ independent continuant)	depends on *RO:0002502*	Human life function *is_* *attribute_of* person	A relation that specified that a person has a human life function
has behavioural goal *BCIOR:000012*	Causally influenced by a cognitive representation of something the behaviour could bring about. (individual human behaviour/cognitive representation)	causally influenced by *RO:0002559*	individual human behaviour *has_behavioural_goal* cognitive representation	A relation that specifies the goal (as a cognitive representation) of a behaviour.
has behavioural outcome *BCIOR:000013*	Causal relation between two entities in which a behaviour brings into existence, causes to occur, destroys, prevents from occurring, or changes an entity. (individual human behaviour/entity)	causal relation between entities *RO:0002506*	individual human behaviour *has_behavioural_outcome* behavioural consequence	A relation that specifies the outcome of a behaviour.
is enacted by *BCIOR:000014*	Has participant that relates a behaviour to the person enacting the behaviour. (individual human behaviour/person)	has participant *RO:0000057*	individual human behaviour *is_enacted_by * person	A relation that specifies a person performing a behaviour.
uses *BCIOR:000015*	Has participant that relates a behaviour to a material entity that the person enacting the behaviour intends to enable or facilitate the behaviour. (individual human behaviour/material entity)	has participant *RO:0000057*	individual human behaviour *uses* material entity	A relation that specifies some object or substance that is used in a behaviour.
has behavioural companion *BCIOR:000016*	Has participant that relates a behaviour to another sentient being that accompanies the person enacting the behaviour. (individual human behaviour/animal)	has participant *RO:0000057*	individual human behaviour *has_behavioural_companion* animal	A relation that specifies a person or animal with whom the person(s) performs the behaviour.
has behavioural target *BCIOR:000017*	Has participant that relates the behaviour to an object that the person enacting the behaviour intends to influence. (individual human behaviour/person)	has participant *RO:0000057*	individual human behaviour *has_behavioural_target* person	A relation that specifies an object, person or animal that a person is trying to influence with their behaviour.
has process part *BFO:0000117*	Inverse of occurrent_part_of which is defined as: b occurrent_part_of c =Def. b is a part of c and b and c are occurrents.		behaviour change intervention *has_process_part* behaviour change intervention outcome behaviour ^†^	A relation that specifies that something is a component or part of a larger process.

^*^Definitions of relations imported from other ontologies can appear obscure, technical or tautological. We decided to import them anyway and provide informal definitions to help users to grasp their meaning. ^#^Domain refers to classes that can be the subject of a relation. Range refers to classes that can be the object of a relation. ^†^ This relation is presented in the upper-level BCIO.

### Step 7: Reviewing the ontology’s coverage by annotating constructs in behavioural theories

To map constructs from the 76 behavioural theories to the BCIO, several changes needed to be made to the HBO. Four classes (‘affiliation behaviour’, ‘avoidance behaviour’, ‘participating in healthcare treatment’ and ‘behavioural reflectiveness’) were changed and, altogether, 50 new classes were added to the ontology. These new classes included 30 individual human behaviours (e.g., ‘coping behaviour’ and ‘monitoring behaviour’), one class for population behaviour (‘collective behaviour’), one class for behavioural patterns (‘behaviour pattern maintenance’) and one class for behavioural change (‘behaviour change through group norm’). A class for ‘behaviour chain’ was also added and distinguished from behavioural pattern, as patterns involve the same type of behaviour, whereas behavioural chains involve causally linked behaviours that can be of different types (e.g., watching television followed by snacking while watching). Additional 15 classes were added to capture various aspects of behaviour, including behavioural consequences and attributes (e.g., ‘reflectively driven’). Finally, one broad class for ‘task complexity’ (definition “A process attribute that is the complexity of thought or behaviour required to achieve a goal.”) was added, as this class can be used to describe the context in which a behaviour is required. A log of the new classes mapped to one or more construct, as well as changed classes can be found here: https://osf.io/qy57j

The resulting ontology had 230 classes, organised onto eight hierarchical levels, made up of the following (see Human Behaviour Ontology version 1: https://osf.io/famkq):

1.    The class ‘individual human behaviour’ and 159 classes organised under it (160 classes overall; see upper-level classes and immediate subclasses in Table 5).

2.    The class ‘behavioural attribute’ and 24 classes under it (25 classes overall; see Table 6).

3.    The 21 classes used to characterise behaviours or abstinence from behaviour (and its parent class ‘personal attribute’), and one for task complexity (22 classes; see classes relevant to behaviour in Table 7).

4.    The class ‘function’ and its subclass ‘human life function’ (two classes).

5.    The class ‘behavioural chain’, the class ‘individual human behaviour pattern’, its two upper-level classes (i.e., its parent class and the subsequent upper-level class), one for pattern maintenance and five lower-level classes illustrating its use in relation to tobacco use behaviour (10 classes overall).

6.    The class ‘individual human behaviour change’, four lower-level classes illustrating the use of the class in relation to tobacco use cessation and behaviour change relating to norms (six classes overall).

7.    The ‘population behaviour’ (its subclass ‘collective behaviour’), ‘population behaviour pattern’, ‘human behaviour’ and ‘behaviour change intervention outcome behaviour’ classes (five classes).

**Table 5.** Upper-level individual human behavioural classes and their immediate subclasses in the HBO.

Upper-level behavioural classes (Level 1)	Behavioural subclasses (Level 2)	Definition
behavioural substitution *BCIO:050846*		An individual human behaviour in which a person substitutes a more desired behaviour for a less desired behaviour.
coping behaviour *BCIO:050809*		An individual human behaviour that has the goal to reduce harm or discomfort.
domestic behaviour *BCIO:050812*		An individual human behaviour within a residential facility.
experience-related behaviour *BCIO:050443*		An individual human behaviour that relates to something the person experiences.
	approach behaviour *BCIO:050841*	An experience-related behaviour that involves taking action to engage with some stimulus that is judged to be rewarding by the person.
	avoidance behaviour *BCIO:036073*	An experience-related behaviour that involves taking defensive action in order to avoid some stimulus judged to be aversive by the person.
	distress minimisation behaviour *BCIO:050421*	An experience-related behaviour that involves avoiding, reducing or escaping anxiety, stress, sorrow, shame and unhappiness.
	emotional behaviour *BCIO:050813*	An experience-related behaviour that is caused by an emotion process.
	enjoyment behaviour *BCIO:036047*	An experience-related behaviour that is performed to experience pleasure or satisfaction.
	learning behaviour *BCIO:036008*	An experience-related behaviour that involves improving knowledge or skill.
	mind-body behaviour *BCIO:036041*	An experience-related behaviour that aims to create a sense of interconnectedness between the mind and the body.
	regulatory behaviour *BCIO:050834*	An experience-related behaviour that involves monitoring and acting upon some parts of the person or their environment.
	sexual behaviour *BCIO:036030*	An experience-related behaviour that involves sexual arousal.
	spiritual behaviour *BCIO:036112*	An experience-related behaviour that is performed in line with a belief system that is grounded in reverence for a supernatural power or powers and provides meaning in life.
expressive behaviour *BCIO:050457*		An individual human behaviour that conveys a thought or feeling.
	creative expressive behaviour *BCIO:036021*	An expressive behaviour that involves consciously using some capability to create or shape an aspect of the environment to express an idea or emotion.
	crying *BCIO:050456*	An expressive behaviour that involves tears, and facial expressions of distress.
	emotionally expressive behaviour *BCIO:050815*	An expressive behaviour that conveys some emotion.
	facial expression behaviour *BCIO:050458*	An expressive behaviour that involves the muscles of the face.
	gesticulatory expressive behaviour *BCIO:050459*	An expressive behaviour involving a movement of the limbs, head or torso.
	human communication behaviour *BCIO:036034*	An expressive behaviour that involves the transmission of information between two or more people.
	laughing *BCIO:050374*	An expressive behaviour showing elation, amusement or scorn by means of facial expressions and repeated sharp exhalations.
	vocalisation behaviour *BCIO:050442*	An expressive behaviour involving vibration of the vocal chords.
goal-directed behaviour *BCIO:050818*		An individual human behaviour that has a behavioural goal.
	approval seeking behaviour *BCIO:050802*	A goal-directed behaviour whose goal is to obtain the approval of another person.
	opportunity-seeking behaviour *BCIO:050920*	A goal-directed behaviour whose goal is to search for and find opportunities that help meet needs or support attaining goals.
	threat-reducing behaviour *BCIO:050840*	A goal-directed behaviour that has a goal to reduce threat.
habitual behaviour *BCIO:006158*		An individual human behaviour that results from a learnt stimulus- behaviour co-occurrence.
harm preventing behaviour *BCIO:050819*		An individual human behaviour that has an outcome to prevent harm.
harmful behaviour *BCIO:036075*		An individual human behaviour that causes net harm.
	harmful behaviour to others *BCIO:050398*	A harmful behaviour that involves interacting with another animal * and thereby causing harm to its health, wellbeing or social functioning.
	self-injury behaviour *BCIO:036014*	A harmful behaviour that involves intentionally causing oneself physical harm.
health-related behaviour *BCIO:050437*		An individual human behaviour that relates to health of oneself or others.
	health-promoting behaviour	A health-related behaviour that improves the person’s health.
	physical performance behaviour *BCIO:036042*	A health-related behaviour that involves maintenance or improvement of flexibility, strength, balance or cardiovascular fitness.
	providing healthcare *BCIO:050413*	A health-related behaviour that involves assessing, monitoring, improving or maintaining an aspect of another person’s health.
	self-monitoring an aspect of health *BCIO:050410*	A health-related behaviour that uses a method to monitor and record an indicator of one’s health or wellbeing.
	utilising healthcare *BCIO:050399*	A health-related behaviour that involves interacting with a healthcare provider in order to assess, monitor, improve or maintain an aspect of one's health.
life function-related behaviour ^#^ *BCIO:050438*		An individual human behaviour that serves vital bodily functions.
	breathing behaviour *BCIO:036057*	A life function-related behaviour involves providing an appropriate level of oxygenation to body tissues.
	excretion behaviour *BCIO:036054*	A life function-related behaviour that involves eliminating excess or harmful chemicals produced by bodily functions.
	reproductive behaviour *BCIO:036056*	A life function-related behaviour that involves producing offspring based on combining DNA of two or more people.
material entity-related behaviour *BCIO:050439*		An individual human behaviour that relates to a material entity.
	consumption behaviour ^†^ *BCIO:036061*	A material entity-related behaviour that involves ingesting material into the body.
	environmental system management behaviour *BCIO:036007*	A material-entity related behaviour that involves creating, maintaining, adapting or destroying aspects of the physical or social environment system.
	object-using behaviour *BCIO:036027*	A material-entity related behaviour that uses a non-living object.
	physical contact behaviour *BCIO:050426*	A material-entity related behaviour that makes physical contact with something.
personal bodily care behaviour *BCIO:036024*		An individual human behaviour that attends to the person’s hygiene, comfort or appearance.
	appearance-based bodily behaviour *BCIO:050372*	A personal bodily care behaviour that attends to making changes to one's body to achieve a desired appearance.
	bodily hygiene behaviour *BCIO:050368*	A personal bodily care behaviour that attends to hygiene by cleaning or washing oneself or parts of the body.
	dressing behaviour *BCIO:050371*	A personal bodily care behaviour that involves wearing clothes providing comfort and protecting oneself from ambient conditions.
	sun protective behaviour *BCIO:050411*	A personal bodily care behaviour that involves protecting one's skin or eyes from the damaging effects of the sun.
position-related behaviour *BCIO:050440*		An individual human behaviour that relates to the enactor's posture or location.
	locomotive behaviour *BCIO:036026*	A position-related behaviour in which muscles are used by a person to move themselves relative to the immediate environment or part of it.
	posture behaviour *BCIO:036029*	A position-related behaviour that involves adopting a body configuration in relation to the immediate environment.
	travel behaviour *BCIO:036059*	A position-related behaviour that involves changing physical location.
reflective behaviour *BCIO:050832*		An individual human behaviour that is caused by a reflective mental process.
socially-related behaviour *BCIO:050441*		An individual human behaviour that relates to the social environment.
	antisocial behaviour *BCIO:036072*	A socially-related behaviour that a population judges to be is contrary to the laws or accepted current norms of social conduct within a specific social context and causes annoyance and or disapproval in others.
	economic behaviour *BCIO:036035*	A socially-related behaviour that involves the production, acquisition, distribution or exchange of money, goods or services.
	inter-personal behaviour ^‡^ *BCIO:036025*	A socially-related behaviour that involves an interaction between two or more people.
	normative behaviour BCIO:006095	A socially-related behaviour that is commonly enacted by people that are part of a social environmental system.
	nurture behaviour *BCIO:036086*	A socially-related behaviour that involves meeting the physical, psychological or social needs of another living being to promote its development.
	political behaviour *BCIO:036089*	A socially-related behaviour that aims to bring about or oppose political or social change.
	pro-social behaviour *BCIO:036066*	A socially-related behaviour that a population judges to accord with current norms of positive social conduct.
	social organisation behaviour *BCIO:036011*	A socially-related behaviour that involves a person contributing to the functioning of a social structure or a person in relation to a social structure.

^*^The term ‘animal’ is used in ontologies to refer to any animal, including humans, who are categorised as animals. ^#^The nutrition function is covered by consumption behaviour. †The parent class of ‘consumption behaviour’ was changed from ‘object-using behaviour’ to ‘material entity-related behaviour’. ‡For the class ‘human communication behaviour’, two parent classes were recorded: ‘interpersonal behaviour’ and ‘expressive behaviour’. However, in the hierarchy, its parent class will be shown as ‘expressive behaviour’.

**Table 6.** Behavioural attributes in the Human Behaviour Ontology.

Label	Definition	Parent class	Informal definition	Comment
behavioural attribute *BCIO:050435*	A process attribute of an individual human behaviour.	process attribute	An attribute of a behaviour.	-
behavioural ease *BCIO:050902*	A behavioural attribute that is the level of convenience, ease or comfort of a behaviour.	behavioural attribute		
behavioural form *BCIO:050430*	A behavioural attribute that is the physical way in which a behaviour is enacted.	behavioural attribute	The way in which the behaviour is performed, including the shape of one’s muscles and skeletal alignment during the behaviour.	-
physical exertion expended on a behaviour *BCIO:050432*	A behavioural attribute that is the level of musculoskeletal work expended on the behaviour to be enacted.	behavioural attribute	The physical effort required to perform a behaviour.	-
high physical exertion expended on behaviour *BCIO:050465*	Physical exertion expended on a behaviour that is high.	physical exertion expended on a behaviour		High physical exertion will mean different things depending on how this concept is operationalised. Therefore, when using this class, you would need to operationalise it for the relevant context (e.g., specify what high exertion means based on the measurement you use).
moderate physical exertion expended on behaviour *BCIO:050473*	Physical exertion expended on a behaviour that is medium.	physical exertion expended on a behaviour		Moderate physical exertion will mean different things depending on how this concept is operationalised. Therefore, when using this class, you would need to operationalise it for the relevant context (e.g., specify what moderate exertion means based on the measurement you use).
low physical exertion expended on behaviour *BCIO:050469*	Physical exertion expended on a behaviour that is low.	physical exertion expended on a behaviour		Low physical exertion will mean different things depending on how this concept is operationalised. Therefore, when using this class, you would need to operationalise it for the relevant context (e.g., specify what low exertion means.
mental exertion expended on a behaviour *BCIO:050431*	A behavioural attribute that is the level of mental effort expended on the behaviour to be enacted.	behavioural attribute	The mental effort required to perform a behaviour.	-
high mental exertion expended on a behaviour *BCIO:050464*	Mental exertion expended on a behaviour that is high.	mental exertion expended on a behaviour	High mental exertion will mean different things depending on how this concept is operationalised. Therefore, when using this class, you would need to operationalise it for the relevant context (e.g., specify what high exertion means based on the measurement you use).	
moderate mental exertion expended on a behaviour *BCIO:050472*	Mental exertion expended on a behaviour that is medium.	mental exertion expended on a behaviour	Moderate mental exertion will mean different things depending on how this concept is operationalised. Therefore, when using this class, you would need to operationalise it for the relevant context (e.g., specify what moderate exertion means based on the measurement you use).	
low mental exertion expended on a behaviour *BCIO:050468*	Mental exertion expended on a behaviour that is low.	mental exertion expended on a behaviour	Low mental exertion will mean different things depending on how this concept is operationalised. Therefore, when using this class, you would need to operationalise it for the relevant context (e.g., specify what low exertion means based on the measurement you use).	
cognitive exertion expended on a behaviour *BCIO:050433*	Mental exertion expended on a behaviour where the exertion involves cognitive processes.	mental exertion expended on a behaviour	The effort relating to thinking required to perform a behaviour.	-
high cognitive exertion expended on a behaviour *BCIO:050462*	Cognitive exertion expended on a behaviour that is high.	cognitive exertion expended on a behaviour		High cognitive exertion will mean different things depending on how this concept is operationalised. Therefore, when using this class, you would need to operationalise it for the relevant context (e.g., specify what high exertion means based on the measurement you use).
moderate cognitive exertion expended on a behaviour *BCIO:050470*	Cognitive exertion expended on a behaviour that is medium.	cognitive exertion expended on a behaviour		Moderate cognitive exertion will mean different things depending on how this concept is operationalised. Therefore, when using this class, you would need to operationalise it for the relevant context (e.g., specify what moderate exertion means based on the measurement you use).
low cognitive exertion expended on a behaviour *BCIO:050466*	Cognitive exertion expended on a behaviour that is low.	cognitive exertion expended on a behaviour		Low cognitive exertion will mean different things depending on how this concept is operationalised. Therefore, when using this class, you would need to operationalise it for the relevant context (e.g., specify what low exertion means based on the measurement you use).
emotional management exertion expended on a behaviour *BCIO:050434*	Mental exertion expended on a behaviour where the exertion involves control over emotions or their expression.	mental exertion expended on a behaviour	The effort a person has to exert to manage their emotions when performing a behaviour.	-
high emotional management exertion expended on a behaviour *BCIO:050463*	Emotional management exertion expended on a behaviour that is high.	emotional management exertion expended on a behaviour		High emotional management exertion will mean different things depending on how this concept is operationalised. Therefore, when using this class, you would need to operationalise it for the relevant context (e.g., specify what high exertion means based on the measurement you use).
moderate emotional management exertion expended on a behaviour *BCIO:050471*	Emotional management exertion expended on a behaviour that is medium.	emotional management exertion expended on a behaviour		Moderate emotional management exertion will mean different things depending on how this concept is operationalised. Therefore, when using this class, you would need to operationalise it for the relevant context (e.g., specify what moderate exertion means based on the measurement you use).
low emotional management exertion expended on a behaviour BCIO:050467	Emotional management exertion expended on a behaviour that is low.	emotional management exertion expended on a behaviour		Low emotional management exertion will mean different things depending on how this concept is operationalised. Therefore, when using this class, you would need to operationalise it for the relevant context (e.g., specify what low exertion means based on the measurement you use).
emotionally driven *BCIO:050814*	A behavioural attribute in which the behaviour is caused by an emotion process.	behavioural attribute		
identity congruence *BCIO:050822*	A behavioural attribute that is the extent to which one’s behaviour is believed to be consistent with one’s core, positive self-identity.	behavioural attribute		
impulsiveness *BCIO:036076*	A behavioural attribute that is to what extent the behaviour is a direct emotional, habitual or instinctive reaction to something.	behavioural attribute	How far a behaviour is enacted without thinking.	This class is a dimension and can be construed as the obverse of reflectiveness and so operationalised in terms of acting without thinking.
intentionality *BCIO:050447*	A behavioural attribute that is the extent to which the behaviour is caused by a behavioural intention.	behavioural attribute	How far a behaviour is enacted as a direct result of a conscious intention to enact it.	This class is a dimension and is differentiated from reflectiveness because a behaviour may be fully intentional but involve little reflective thought, e.g., when driving carelessly. In this class the intention relates to the behaviour itself. If a person intends to do one thing but accidentally does something else it does not count as intentional in this class, e.g., if someone intends to injure someone else and ends up killing them, that would not count as intentionally killing them.
behavioural reflectiveness *BCIO:050444*	A behavioural attribute that is the degree to which the behaviour is under the control of reflective motivation.	behavioural attribute	How far a behaviour is enacted after thinking about it and its consequences.	This class is a dimension and involves any conscious thought processes that lead to a behaviour in some way, even if those processes are themselves influenced by emotional processes and biases.
reflectively controlled *BCIO:050833*	Behavioural reflectiveness in which the behaviour is predominantly controlled by reflective motivation.	behavioural reflectiveness		
response cost *BCIO:050835*	A behavioural attribute that is the time, effort, financial cost, or aversiveness of enacting a behaviour.	behavioural attribute		

**Table 7.** Additional classes for characterising behaviours or abstinence from behaviour, and one for task complexity.

Label	Definition	Parent class	Informal definition
number of behavioural occurrences *BCIO:050429*	A data item that is about the number of times a behaviour has occurred.	data item	Number of times a person performs a behaviour.
behavioural frequency *BCIO:050428*	A data item that is about the number of times a behaviour occurs in a time period.	data item	Number of times a person performs a behaviour within a specific period.
behavioural duration *BCIO:050455*	A temporal interval within which an individual human behaviour occurs.	one-dimensional temporal region (temporal interval)	The time between the start and end of a behaviour.
behaviour starting point *BCIO:050454*	A temporal region that is the start of an individual human behaviour.	temporal region	A time point when a behaviour starts.
behaviour end point *BCIO:050453*	A temporal region that is the end of an individual human behaviour.	temporal region	A time point when a behaviour ends.
abstinence from a behaviour *BCIO:050451*	A personal attribute in which a person does not engage in a behaviour during a time period.	personal attribute	Not performing a behaviour for some period of time.
abstinence duration *BCIO:050449*	A temporal interval during which a person is abstinent from a behaviour.	one-dimensional temporal region (temporal interval)	The time a person is abstinent from a behaviour.
abstinence start point *BCIO:050452*	A temporal region that is the start of an abstinence period.	temporal region	A time point when a person starts being abstinent from a behaviour.
abstinence end point *BCIO:050450*	A temporal region that is the end of an abstinence period.	temporal region	A time point when a person stops abstaining from a behaviour.
behavioural disposition *BCIO:050416*	A bodily disposition that is realised as some behaviour.	bodily disposition	A tendency to behave in a particular way.
behavioural consequence *BCIO:050806*	An entity that is an outcome of behaviour.	entity	
harm prevention *BCIO:050820*	A behavioural consequence in which harm is prevented.	behavioural consequence	
impact of behaviour on environment *BCIO:050823*	A behavioural consequence that involves an outcome relating to the environment system.	behavioural consequence	
negative behavioural consequence *BCIO:050919*	A behavioural consequence that is negatively evaluated by an individual or a population.	behavioural consequence	
positive behavioural consequence *BCIO:050828*	A behavioural consequence that is positively evaluated by an individual or a population.	behavioural consequence	
social behavioural consequence *BCIO:050900*	A behavioural consequence of a member of the person’s social environmental system.	behavioural consequence	
family behavioural consequence *BCIO:050899*	A social behavioural consequence of some family member.	social behavioural consequence	
wider community behavioural consequence *BCIO:050901*	A social behavioural consequence that is beyond the person’s family.	social behavioural consequence	
reducing discomfort *BCIO:050830*	A process in which physical or mental uneasiness or distress is reduced.	process	
reducing harm *BCIO:050831*	A process in which damage or injury is reduced.	process	
task complexity *BCIO:050839*	A process attribute that is the complexity of thought or behaviour required to achieve a goal.	process	

### Step 8: Making the ontology machine-readable and available online

**Figure 2.**
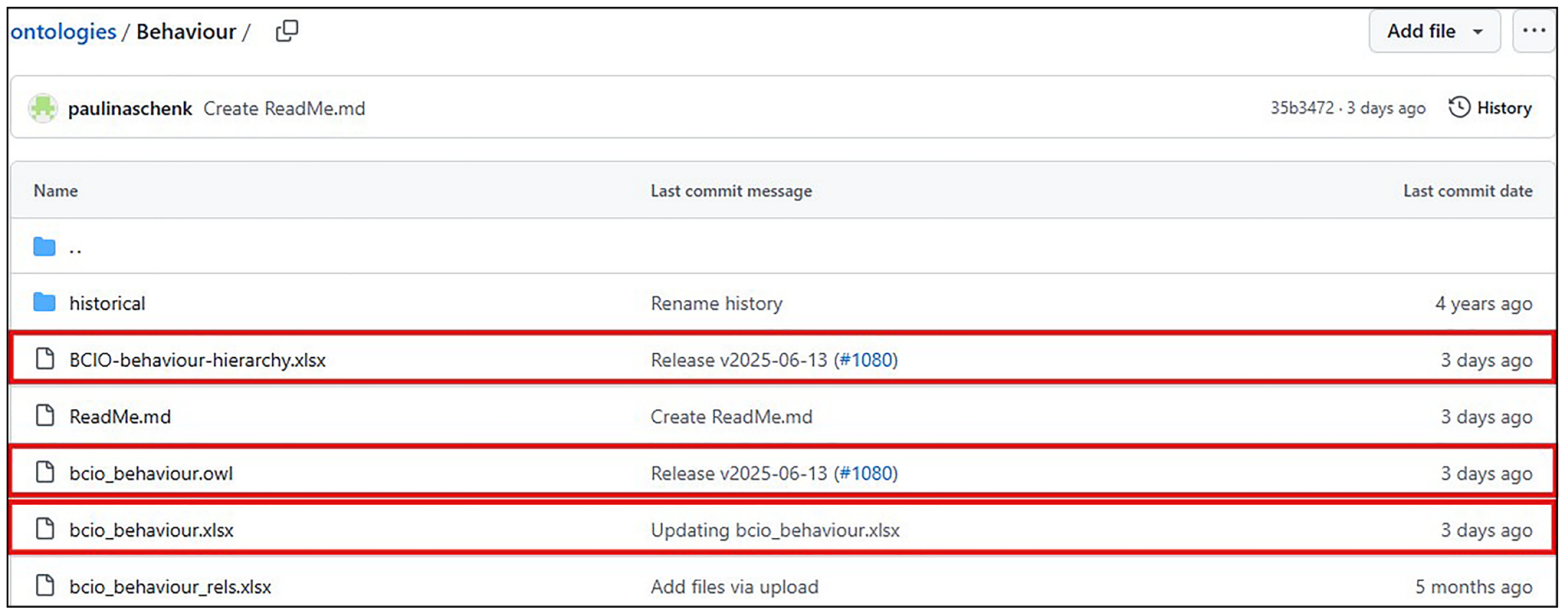
Screenshot of HBO files on GitHub.

The revised version of the HBO, with its 230 classes and 17 relations, was deployed on OSF ( https://osf.io/famkq) and GitHub ( https://github.com/HumanBehaviourChangeProject/ontologies/tree/master/Behaviour). On the GitHub link (see view in Figure 2), the file labelled ‘BCIO-behaviour-hierarchy.xlsx (in a hierarchical format) or ‘ bcio_behaviour.xlsx’ can be opened or downloaded to find the **most recent version** of the ontology as a spreadsheet. The OWL version can be found and downloaded in the file labelled ‘ bcio_behaviour.owl’ for ontology users interested in viewing the ontology on Protégé ( https://protege.stanford.edu/).

The ontology is also accessible through BCIO Search ( https://www.bciosearch.org/) to support browsing and viewing individual classes and visualised through BCIOVisualise ( https://bciovis.hbcptools.org/). In addition, the HBO, as part of the wider BCIO, is also available on Ontology Lookup Service (OLS): https://www.ebi.ac.uk/ols4/. This platform allows users to browse the BCIO (including the HBO) and other ontologies, to identify relevant content from various ontologies and identify their links. The ontology is a **live document**, and further classes and relations will be added and updated on **the ontology’s versions on GitHub, BCIOSearch** and **BCIOVisualise**. The ontology can be applied to behaviour change intervention reports with an annotation guidance, refined in Step 5 (available at https://osf.io/6e2c7). A bespoke BCIO website ( https://www.bciontology.org/) has been created to provide easy access to ontology tools and to bring together information about all the ontologies, including the HBO, into one place.

**Figure 3.**
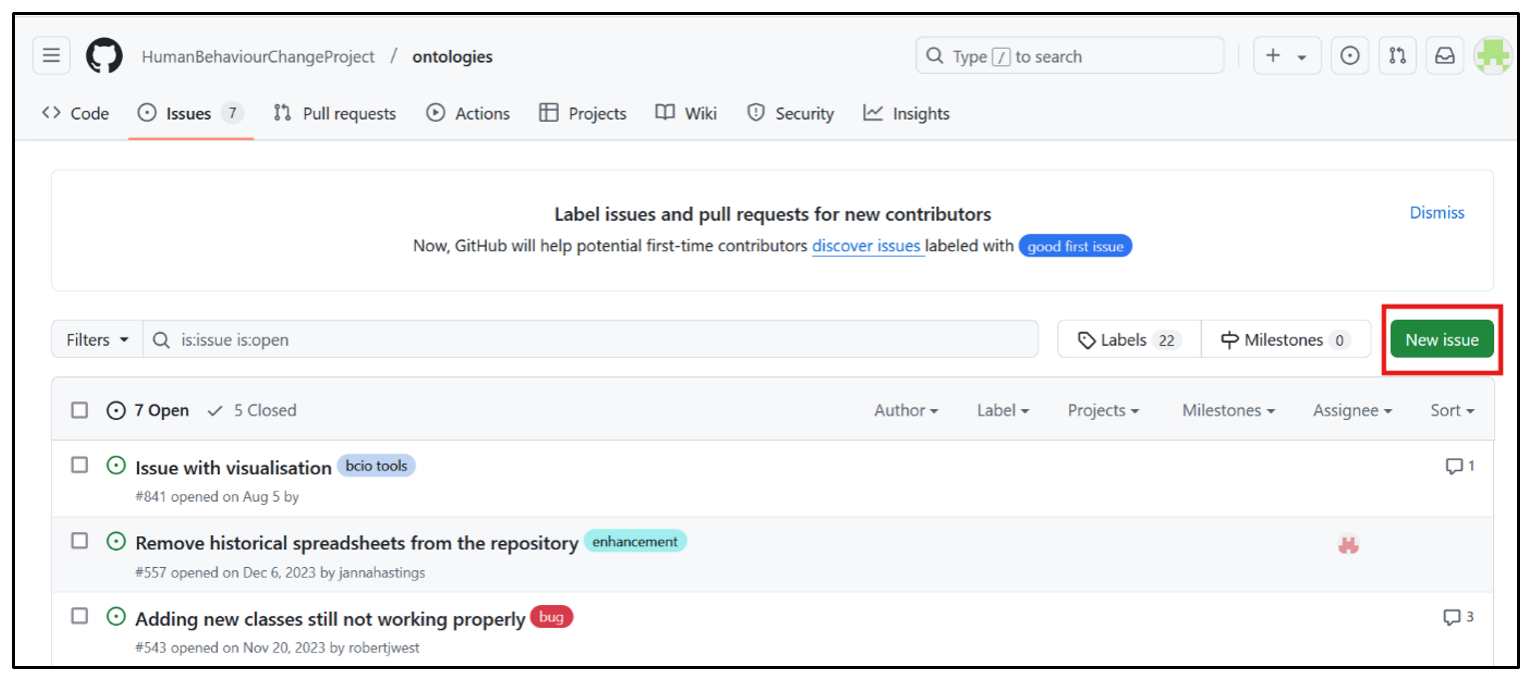
Screenshot of the BCIO Issue Tracker on GitHub.

**Figure 4.**
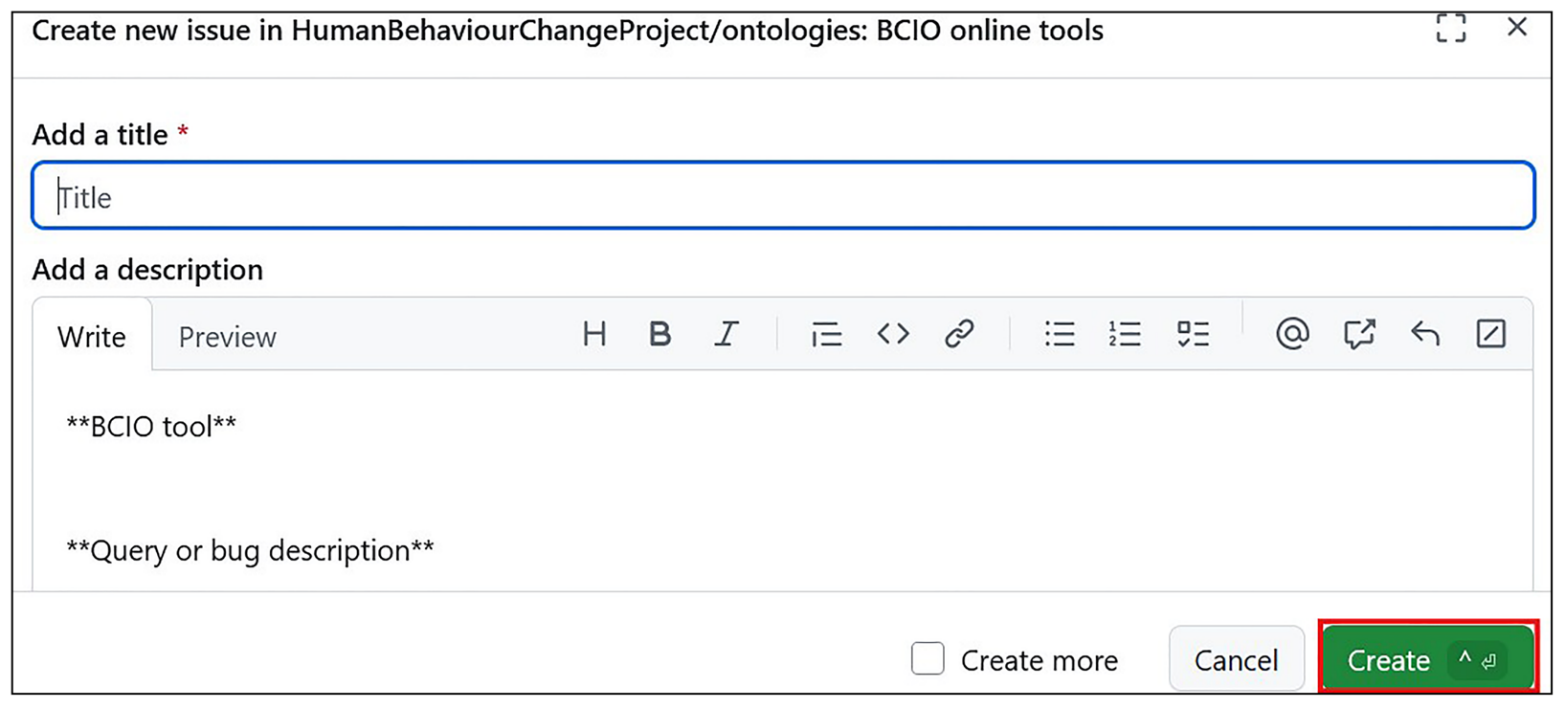
Screenshot of page to describe and submit a new issue on the GitHub BCIO Issue Tracker.

To suggest changes to the ontology, the GitHub Issues Tracker for the BCIO should be used ( https://github.com/HumanBehaviourChangeProject/ontologies/issues). On the GitHub Issue Tracker page, ontology users can submit their feedback on the ontology by clicking the button labelled “New Issue” (see Figure 3). They then need to provide a title for their issue, signpost which BCIO tool their issue is about (e.g., BCIOSearch or ontology class) and provide a description of this issue and, if relevant, what change needs to be made and why (see Figure 4). This issue tracker is used for the wider BCIO, with issues for any lower-level ontology (e.g., the HBO) being submitted and reviewed through this page.

## Discussion

The Human Behaviour Ontology (HBO) consisted of 230 classes and 17 relations. The classes were organised onto eight hierarchical levels to specify behaviours and details about them, as part of the BCIO ( Michie *et al.*, 2017; Michie *et al.*, 2020). The upper-level subclasses of ‘individual human behaviour’ were: (1) experience-related behaviour, (2) expressive behaviour, (3) reflective behaviour, (4) harmful behaviour, (4) health-related behaviour, (5) harm preventing behaviour, (6) coping behaviour, (7) domestic behaviour, (8) goal-related behaviour, (9) habitual behaviour, (10) health-related behaviour, (11) life function-related behaviour, (12) material entity-related behaviour, (13) personal bodily care behaviour, (14) position-related behaviour, (15) socially-related behaviour and (16) behavioural substitution. In addition, classes to characterise behaviours (e.g., ‘behavioural attribute’ and ‘number of behavioural occurrences’), and to broadly capture individual human behaviour patterns, behavioural chains, population behaviour, population behaviour patterns and abstinence from behaviour were included. Only a small fraction of behaviours that can be conceived of, or used in behavioural research, were included at this stage, but the ontology was designed to provide a framework that could form a useful basis for defining any of these, either as simple subclasses of behaviours in the existing ontology or as logically defined classes in which classes and relations are combined to form expressions.

Inter-rater reliability was found to be α = 0.63 for those familiar with the ontology and, following updates to the ontology and annotation guidance, acceptable for those unfamiliar with the ontology (α = 0.74). This suggests that the HBO and associated annotation guidance can be applied with at least acceptable consistency. However, with the expansion of the ontology in Steps 6 and 7, more detailed annotation guidance might be needed to support users (see Future Directions).

Several of the classification systems of behaviour identified as part of this study are useful for specifying behaviours within a certain domain, e.g., the International Classification of Health Interventions (ICHI; Fortune *et al.*, 2021) for behaviours relating to health. Building on these frameworks, the scope of the HBO is more extensive, covering behaviours relating to various domains, such as inter-personal and social dynamics, environment, and economics. The HBO can thus serve as a shared language to clearly specify, label and define behaviours when investigating and reporting them. For instance, by using this ontology when writing protocols, researchers can describe behaviours they are intending to investigate more precisely and use unique identifiers to unambiguously specify their target behaviour(s). With its extensive behavioural classes and detail, the ontology can also support categorising behaviours more precisely when synthesising evidence from various sources and predicting outcomes of interventions. The ontology’s technical facilities also enable developing algorithms for searching, information extraction and prediction about behaviour ( Hastings *et al.*, 2023; Michie *et al.*, 2017). Therefore, the ontology presents a basis for a potentially unifying classification about behaviour that is also computer readable, with opportunities for ontology users to provide feedback to refine the classes and structure ( Arp *et al.*, 2015; He *et al.*, 2018). Future work can also support aligning the ontology to broader (e.g., health or sustainability) frameworks that include relevant behavioural content, such as the Human Phenotype Ontology ( Talapova *et al.*, 2023), Cognitive Atlas ( Miller *et al*., 2010; Poldrack *et al.*, 2011) and The Cognitive Paradigm Ontology ( Turner & Laird, 2012).

The HBO forms part of the BCIO, currently comprising 11 other component ontologies: behaviour change techniques ( Marques *et al.*, 2023), mechanisms of action ( Schenk *et al.*, 2024), mode of delivery ( Marques *et al.*, 2020), source of delivery ( Norris *et al.*, 2021), style of delivery ( Wright *et al.*, 2023), dose (in preparation), schedule of delivery ( Marques *et al*., 2024), engagement (in preparation), fidelity (in preparation), setting ( Norris *et al.*, 2020) and target population ( Wright *et al*., 2025). These ontologies can be used together to organise and synthesise detailed evidence about various aspects of behaviour change intervention scenarios and their evaluations. Classes in the HBO can also be linked and reused by ontologies beyond the BCIO, such as the Addiction Ontology ( Hastings *et al.*, 2020).

As part of the Human Behaviour-Change Project, a foundry for ontologies in behavioural and social sciences has been established ( https://www.bssofoundry.org/; Hastings *et al.,* 2024). Collaborations formed through the BSSO Foundry can be used to support the development of alignments between ontological frameworks on behaviour. For instance, the TURBBO Project has reused some classes from the HBO in their ontology, specifying evidence-based relations between behavioural classes ( https://sites.google.com/sheffield.ac.uk/turbbo/additional-resources#h.4k2unbgh9vh3). In turn, the relations specified and refined in the TURBBO Project can inform enhancements to the relations between HBO classes and provide examples for applying the ontology to organise research data. More specific ontologies in the field, e.g., about physical activity ( Braun *et al.*, 2023; Braun *et al*., 2025; Kim *et al.*, 2019), could use the HBO’s classes (e.g., ‘individual human behaviour’), where relevant, and add more granular classes needed for their application (e.g., swimming). Feedback from the developers of these ontologies will, in turn, also refine the BCIO. The BSSO Foundry aims to support and strengthen a community of practice around behavioural and social science ontologies, enabling more collaborative, open and inclusive ontology development and refinement ( Hastings *et al*., 2024).

### Strengths and limitations

A strength of the HBO’s development is that it drew on diverse resources, including existing behavioural frameworks and other ontologies, published studies on behaviours and expert feedback. Thus, a range of perspectives was considered when developing and organising the 230 classes included in the ontology. This means that the HBO is likely to be a useful tool to a wide audience interested in behavioural science ( Amith *et al.*, 2018; http://obofoundry.org/principles/fp-010-collaboration.html). Another strength was structuring the HBO by drawing on the Basic Formal Ontology ( Arp *et al.*, 2015), as this enables future collaborations with other ontology developers using the same upper-level structure (e.g., Carlier *et al.*, 2022; Hastings *et al.*, 2020).

The use of ontological relations, beyond the hierarchical relations used in taxonomies, also allows users to characterise behaviours in detail. Some examples of applying the ontology’s classes and relations are provided in Table 8. It should be noted that ontology users might need to suggest new classes to capture the exact information they are interested in.

**Table 8.** Examples for using classes and relations in the HBO and other ontologies to specify behaviours in detail.

No	Description of behaviour targeted in an intervention	Behaviour expressed through formal relations between classes *
1	An intervention aims to increase participation in physical therapy in a healthcare setting for three hours.	• participating in physical therapy (BCIO:050405) ***is enacted by*** (BCIOR:000014) person (MF:0000016) • participating in physical therapy (BCIO:050405) ***occurs in*** (BFO:0000066) health care facility (OMRSE:00000102) • participating in physical therapy (BCIO:050405) ***occupies temporal region*** (BFO:0000155) behavioural duration (BCIO:050455)
2	An intervention to increase the frequency of woman walking in parks with their friends, in order to increase happiness.	• walking (BCIO:036108) ***is enacted by*** (BCIOR:000014) person (MF:0000016) ***has attribute*** (RO:0000053) female gender (BCIO:010111) • walking (BCIO:036108) ***occurs in*** (BFO:0000066) park (ENVO:00000562) • walking (BCIO:036108) ***has behavioural companion*** (BCIOR:000016) person (MF:0000016) ***has role*** (RO:0000087) friend (BCIO:010101) • walking (BCIO:036108) ***has behavioural outcome*** (BCIOR:000013) happiness (MFOEM:000042)
3	An intervention to target healthcare professionals prescribing behaviour for medications to a patient in order for him/her/them to fully recover from their condition.	• prescribing medication (BCIO:050355) ***is enacted*** **by** (BCIOR:000014) person (MF:0000016) ***has role*** (RO:0000087) health professional (BCIO:010008) • prescribing medication (BCIO:050355) ***has behavioural target*** (BCIOR:000017) person (MF:0000016) ***has role*** (RO:0000087) patient role (OBI:0000093) • prescribing medication (BCIO:050355) ***has behavioural goal*** (BCIOR:000012) complete remission (OGMS:0000120)

*Each of the examples capture interventions’ behavioural outcomes, therefore, in addition to the presented classes, the class ‘behaviour change intervention outcome behaviour’ would be annotated for these examples.

As the literature around behaviours is vast and behaviours can be specified at various levels of complexity, a challenge was deciding on the sources to draw on to identify and refine classes in the HBO. To ensure that the ontology’s structure is broad enough to allow the addition of more detailed classes in the future, we drew on behavioural frameworks and broad behavioural journals in Steps 2 and 3 and invited a range of behavioural scientists in Step 4. To test the inter-rater reliability of the ontology’s application, a pragmatic approach was used to select papers, namely papers that referenced – in any section - the BCTTv1 or the Theoretical Domains Framework. While these frameworks are widely cited, they are more commonly used for health behaviours and in certain countries (e.g., the UK), introducing potential bias into the papers included in the annotations using the HBO for the inter-rater reliability calculations. In addition, the selection of more recent papers, assumed to have clearer descriptions of behaviour, may have resulted in a higher inter-rater reliability than for older papers. This choice was made to allow us to test the use of the ontology itself, rather than reporting a lower inter-rater reliability due to the poor reporting of behaviours. Even so, there was considerable variation in the quality of descriptions across papers included in the current study. In future work, we will further investigate the ontology’s application on other corpuses of papers, and encourage other researchers to test the ontology, thereby improving the generalisability and usability of the ontology.

A related challenge was deciding how much detail needed to be added to the ontology without making it too complex and thereby difficult to use. Complex ontologies developed as part of the BCIO, such as the Mode of Delivery Ontology ( Marques *et al.*, 2020) and the Mechanism of Action Ontology ( Schenk *et al.*, 2024), were found to be more difficult to reliably apply than ontologies with simpler structures, such as the Intervention Source ( Norris *et al.*, 2021) and Setting ( Norris *et al.*, 2020) Ontologies. For this reason, we attempted to capture behaviours through broad classes, such as economic and political behaviour. However, more granular classes will be needed when reporting or synthesising information about specific behavioural domains. The examples recorded for these behavioural classes in the HBO can provide a starting point for ontology users to suggest new classes on the GitHub repository in the future. Moreover, other ontology developers could expand on these behavioural classes for their specific applications and suggest additions to the HBO.

As behaviours can be classified in various ways that are useful in different contexts, there is often overlap between classes. For instance, the class ‘walking’ is always a ‘locomotive behaviour’ but can sometimes also be a ‘travel behaviour’ or a ‘physical performance behaviour’. We only specified parent-child class relations (‘is_a’) if this relation would always hold, e.g., ‘walking’ is_a ‘locomotive behaviour’. While the other relations between behaviours and other classes were discussed in detail and iteratively refined, they were not tested in a specific use case. Future studies would need to investigate to what extent these relations can be understood and reliably applied, particularly by users who are new to ontologies. Finally, our annotation guidance can support ontology users to consistently apply these classes, e.g., annotating the most specific applicable class or annotating two classes where relevant to capture more information about a behaviour. Such guidance can be tailored based on the aims of a particular research project.

## Future directions

This is the first published version of the ontology. The ontology’s development and maintenance are iterative processes; no ontology is ever ‘finished’. The HBO will continue to evolve and is intended to provide a framework within which new classes can be added as required, or definitions can be updated. In some cases, based on the sources we drew on, behaviours have been defined to a level of granularity that would be adequate to characterise a behaviour while in others, classes need to be added as required. We hope that other domain experts will extend parts of the HBO by developing their own ontologies or suggest classes to add to the ontology.

Through wider application and testing of these classes and relations, we hope to increase these classes’ applicability and provide more guidance. As outlined above, to contribute new classes to the ontology, users should suggest the new class by creating a “New Issue” on the GitHub portal for the BCIO ( https://github.com/HumanBehaviourChangeProject/ontologies/issues). These issues will be monitored by the ontology developers, as a large new international project has been funded between 2024 and 2029 to advance, expand and increase the usability and interoperability of the BCIO: The Advancing Prevention Research in Cancer through Ontology Tools (APRICOT) Project ( Michie *et al*., 2024). This research team, including the developers of the current ontology, will review and respond to each issue and, where needed, update the HBO classes and structure. The log of issues and responses will be open to anyone to view through the GitHub Issue tracker. Expert users, e.g., expanding a part of the HBO (e.g., around communication behaviour), could also be given access to edit the ontology on GitHub as collaborators, allowing them to submit content directly. A study to explore the usability of the BCIO and its tools has been conducted, and is currently being prepared for publication.

We are also planning to further refine the behavioural attributes and relations, which have been proposed as a starting point in the HBO. For example, the classes for behavioural attributes around timing, such as behavioural duration, could be further developed to capture statistical values, such as minimum, maximum and mean duration of behaviour. This work will be informed by drawing on the lower-level ontology for intervention schedule of delivery ( Marques *et al.*, 2024) and applying the attributes to annotate details about behaviours in the literature. More specifically, in the APRICOT Project, we are hoping to develop the classes needed to precisely capture 24-hour movement behaviours (sedentary activities, physical activity and sleep-relate behaviours), serving as a use-case for applying and developing classes on behaviour and their attributes. This use-case can also inform adding more relations between specific classes to improve the logical reasoning that can be done using the HBO. In this current version of the ontology, we kept the structure relatively simple (e.g., limiting multiple parent-child class relations) to allow behavioural researchers to familiarise themselves with the ontology.

As the ontologies’ content can change over time, we recommend that ontology users report the publication date of the ontology version that they applied to their work. Users should also always report the classes’ unique identifiers since class labels and definitions can change over time (e.g., based on user feedback or new scientific evidence), but the unique identifier remains the same.

For future ontology development work, more inclusive and participatory approaches to ontology development should be considered. As Norris *et al*. (2021) underlined, stakeholder involvement is often underutilised in ontology development. The methods for developing the BCIO and its lower-level ontologies included explicit steps to involve stakeholders and transparently integrating their feedback ( Wright *et al*., 2020). As part of the GALENOS Project, which is developing a mental health ontology, there were efforts to further integrate participatory approaches into building the Mental Health Ontology ( Schenk *et al*., 2024). This work is incorporating biannual feedback from an international advisory group, who are reviewing the methods and, where feasible, the results of the work to structure this ontology. More broadly, the APRICOT Project aims to build a community of practice for ontology development and application. This will support creating more accessible and globally relevant practices and standards around developing and using ontologies in behavioural and social research.

## Conclusion

The HBO is a logically structured classification framework that characterises a wide range of human behaviours in the context of behaviour change interventions and beyond. This ontology can be used for detailed and precise reporting, evidence synthesis about behaviours and predicting intervention outcomes. Through refinements based on feedback from its users and collaborations with the other ontology developers, the HBO will become more reflective of wider views about behaviour. This ontology contributes to building a more unified and clear language to communicate about behaviours and hence advancing the science of behaviour and behaviour change.

## Ethics and consent

For this stakeholder review, ethical approval was granted by University College London’s Research Ethics Committee (CEHP/2020/579) in February 2020. Participants provided informed written consent via an online Qualtrics survey before starting the review.

## Data Availability

Open Science Framework: Human Behaviour-Change Project. https://doi.org/10.17605/OSF.IO/EFP4X ( West *et al.*, 2020) This project contains the following underlying data: Expert feedback on Human Behaviour Ontology; Raw feedback received from behavioural science and ontology experts; https://osf.io/r8g6a It should be noted that the stakeholders who are named in this section provided permission to be named. Open Science Framework: Human Behaviour-Change Project. https://doi.org/10.17605/OSF.IO/EFP4X ( West *et al.*, 2020) This project contains the following extended data: The list of systematic reviews and abstracts used in Step 3 to refine the ontology by applying it to relevant literature; https://osf.io/hsvp4 Expert feedback survey; Full survey provided to behaviour science experts in the review in Step 4; https://osf.io/dcmq4 The details of the method to identify papers to annotate behaviours with the Human Behaviour Ontology in Step 5; https://osf.io/csw65 Papers used in development of the Human Behaviour Ontology in Step 5 to test inter-rater reliability using the ontology; https://osf.io/rdw9f The details of the method to identify measurement scales to annotate behaviour attributes in Step 6; https://osf.io/n7vhb The classes hierarchically organised in the initial version of the Human Behaviour Ontology in Step 2; https://osf.io/v869m Record of the allocation of example behaviours that were extracted from the literature in Step 3; https://osf.io/7scz6 The classes hierarchically organised in the Human Behaviour Ontology at the end of Step 3; https://osf.io/v9ymr The classes hierarchically organised in the Human Behaviour Ontology at the end of Step 4; https://osf.io/awgtp Inter-rater reliability testing for annotations by researchers familiar with the Human Behaviour Ontology; https://osf.io/95rbx The issues recorded by researchers familiar with the Human Behaviour Ontology when applying it to annotate behaviours in interventions reports in Step 5 and responses to these issues; https://osf.io/yrnph Inter-rater reliability testing for annotations by researchers unfamiliar with the Human Behaviour Ontology; https://osf.io/ktfys The classes hierarchically organised in the Human Behaviour Ontology at the end of Step 5; https://osf.io/rxcuf Log of changes made to upper-level classes in the Human Behaviour Ontology in Step 6; https://osf.io/2wdsa Upper-level individual human behaviour classes and their immediate subclasses in Step 6; https://osf.io/cqe2w Initial list of behaviour attributes generated in Step 6; https://osf.io/dmu52 The issues recorded when applying the behaviour attributes to annotate measures of behaviour in Step 6 and responses to these issues; https://osf.io/945nb Log of the classes added or changed in Step 7 and the relevant theoretical constructs they were mapped onto; https://osf.io/qy57j Coding guidelines; Manual for coding using the Human Behaviour Ontology; https://osf.io/6e2c7 The updated completed version of the Human Behaviour Ontology reported in the paper; https://osf.io/famkq OSF page for the Human Behaviour-Change Project; Homepage for all outputs across the project; https://osf.io/h4sdy/ Zenodo: HumanBehaviourChangeProject/ontologies: HumanBe-haviourChangeProject/ontologies: Behaviour Change Technique Ontology, Mechanism of Action Ontology. https://doi.org/10.5281/zenodo.14882463 ( Hastings *et al.*, 2025) Data are available under the terms of the Creative Commons Attribution 4.0 International license (CC-BY 4.0).

## Software availability

Source code used to calculate alpha for IRR available from: https://github.com/HumanBehaviourChangeProject/Automation-InterRater-Reliability.

Archived code at time of publication: https://doi.org/10.5281/zenodo.3833816 ( Finnerty & Moore, 2020)

License: GNU General Public License v3.0 only
