# Supplementary material for: An ontological framework for organising and describing behaviours: The Human Behaviour Ontology
Source: Wellcome Open Res. 2025 Jun 30;9:237. Originally published 2024 May 8. [Version 2] doi: 10.12688/wellcomeopenres.21252.2 (PMC12334917; doi:10.12688/wellcomeopenres.21252.2)
Supplement: Peer review history

# Reviewer report: Reviewer response for version 2

**DOI:** 10.21956/wellcomeopenres.27065.r126811

**Published:** 2025-08-08

**Authors:** Mor Peleg

**Affiliations:** 1 University of Haifa, Haifa, Haifa District, Israel

The authors did a great job at attending to all of my comments. There is a single minor comment remaining:

 1) Emotional_management_exertion_expended_on_a_behaviour is a subclass of Individual_human _behaviour in the OWL but not in Table 5.

Is the work clearly and accurately presented and does it cite the current literature?

Partly

If applicable, is the statistical analysis and its interpretation appropriate?

Not applicable

Are all the source data underlying the results available to ensure full reproducibility?

Yes

Is the study design appropriate and is the work technically sound?

Yes

Are the conclusions drawn adequately supported by the results?

Partly

Are sufficient details of methods and analysis provided to allow replication by others?

Yes

Reviewer Expertise:

ontologies, clinical decision support, computer-interpretable guideline, digital behavior change interventions

I confirm that I have read this submission and believe that I have an appropriate level of expertise to confirm that it is of an acceptable scientific standard.

# Reviewer report: Reviewer response for version 1

**DOI:** 10.21956/wellcomeopenres.23505.r82458

**Published:** 2024-07-30

**Authors:** Jennifer Inauen

**Affiliations:** 1 University of Bern, Bern, Switzerland

**Summary:**

 This paper reports on the development of the human behavior ontology, a classification system for human behaviors, which will aid the advancement of a systematic science of behavior change. The 7-step iterative process of developing the ontology was explained in detail and with high transparency, linking to documents that enable the reader to verify the different versions and changes to the ontology during the process. This paper was very well written, and introduced key terms, so that readers unfamiliar with ontologies (like me) can follow. Congratulations to the authors for this impressive work, which I believe will be highly influential in the field of behavior change. I added comments below that will hopefully help to further improve clarity and understanding.

**Abstract:**

- Is “individual human behavior” the uppermost class? This could be clarified.
- There seems to be some overlap in the upper-level classes, which confused me. For example, physical activity could relate to health as well as position. This issue is explained in the discussion, but perhaps a comment could be added in the abstract as well.
- All presented classes are behaviors, except “behavioural attributes”. It wasn’t immediately clear to me why this would be included here. Perhaps this could be explained here.

**Introduction:**

- The introduction presents compelling arguments why an ontology of behavior is needed, citing the current literature adequately. Regarding the scope of the ontology, I found discrepant information in different parts of the manuscript. For example, in the introduction, the authors state that the aim of this paper is to create a human behavior ontology with regards to interventions. On the other hand, the scope of the ontology was defined more broadly under Step 1 in the results section. Personally, I would agree with the broad definition of the scope. In any case, it should be consistent.

- The description of ontologies is easy to understand, and the glossary in Table 1 is helpful to keep in mind the definitions of the terms that might not yet be familiar to many behavior change experts.
- The authors mention the TURBO project. How does this differ from the Human Behavior Change project? Do the two projects complement each other?

**Methods:**

- Step 2 (identifying candidate classes): It seems a daunting task to arrive at a set of classes to describe all human behaviors (relevant to interventions). How can the authors be sure that they have included all the behaviors? Would it have been advisable to more systematically include experts from different behavioral domains at this stage?
- Somewhat related to the above comment, in Step 3, the authors chose to focus on what they call “broad” journals of behavior (e.g. Nature Human Behavior) to sample articles to code using the HBO prototype. What was the rationale for focusing on the broad journals? Is there a risk that the ontology lacks specificity due to this choice? Actually, the rationale seems to come a bit later in the description of Step 3. It seems that the authors first tried to get what they call a “stable structure” of the ontology before going into too much detail. This could perhaps be made clear a priori, also briefly mentioning why that is important, in order to provide a clear rationale for the overall procedure of this Step and my previous point about the experts.
- For Step 4, the authors ensured geographical diversity of experts, which is great. However, I was wondering about the diversity of their expertise in terms of behavior change (see comment above). As the stakeholders were asked about missing classes, it would seem highly relevant that stakeholders were experts in different behavioral domains. Please provide information on this, discuss to what extent the behavioral domains were comprehensively covered, and how this might have influenced the ontology.
- Why was the database “Open Alex” chosen for selecting papers for Step 5 rather than more common databases of scientific publications (e.g. those chosen for Step 3)? Please provide a brief rationale.
- Further for Step 5, I wondered whether the inclusion criteria to only include papers that referenced the BCT taxonomy v1 or the theoretical domains framework might have limited the test of the ontology. These two papers are certainly seminal in the field of behavior change. However, this choice might have inadvertently limited the test to research that has used these frameworks, and might have neglected research using other behavior change frameworks. It would seem to extend the claim of comprehensiveness and generalizability of the ontology to include research using other frameworks as well.
- Additionally for Step 5, the authors mentioned that they only included recent papers because their behavioral descriptions might be clearer. Does this mean that the interrater reliability estimate might be a more optimistic estimate, i.e. that we might not necessarily expect the ontology to be as applicable to earlier work because of lack of clarity of earlier papers? That would be interesting to understand, considering that systematic reviews that might use the ontology would usually go back further in time (e.g. by coding some examples from earlier periods).
- Overall, the reporting of the inclusion criteria and paper selection for Step 5 could be written a bit more clearly. In particular, it would be most helpful to mention the inclusion criteria of the papers in one place (right now they are spread over a multiple paragraphs). Perhaps a flow diagram might be helpful (possibly in the supplementary document on this step).
- What was the rationale for selecting 100 papers for the interrater reliability? Was this related to statistical power?
- There’s a typo in Step 7: A “be” is missing in “can prepared” in the sentence “A ROBOT template is a comma-separated values (CSV) file that can prepared using spreadsheet software (e.g., Excel) for conversion from spreadsheet columns into OWL language and metadata attributes.”

**Results:**

- There’s a typo in Step 2: An “is” is missing in “The first iteration of the HBO recognised that a distinction needed to be made between behaviours of individuals and of populations.”
- In Step 2, it would be helpful to clearly indicate what the five levels are. It becomes clear with the linked OSF document, but it would be helpful to indicate the labels in the text. It’s also not clear to me, how exactly the authors came up with the five levels. This wasn’t described in enough detail in the methods either.
- Steps 4 and 5: Am I understanding the text correctly that the function-related classes were removed in Step 4 and then added again in Step 5? Why if they “did not capture behaviors”?
- Step 6: The 34 upper-level classes were collapsed into 9 classes. Is it a problem that there seems to be no clear distinction between some of these 9 classes? For example, it looks like behaviors from some upper-level classes could be considered as belonging to the health class as well. The authors discuss this later, but perhaps it would be useful to mention it here already that they only defined parent classes if they always held.
- Also in Step 6, I couldn’t understand where the class “individual human behaviour change” came from, or where they were illustrated.

**Discussion:**

 This section is very well written, provides useful examples, and outlines how the ontology can be used and cited. I have no additional comments to those mentioned above.

Is the work clearly and accurately presented and does it cite the current literature?

Partly

If applicable, is the statistical analysis and its interpretation appropriate?

Yes

Are all the source data underlying the results available to ensure full reproducibility?

Yes

Is the study design appropriate and is the work technically sound?

Yes

Are the conclusions drawn adequately supported by the results?

Yes

Are sufficient details of methods and analysis provided to allow replication by others?

Partly

Reviewer Expertise:

I am a behavior change expert. I was not familiar with ontologies prior to this review.

I confirm that I have read this submission and believe that I have an appropriate level of expertise to confirm that it is of an acceptable scientific standard, however I have significant reservations, as outlined above.

# Reviewer report: Reviewer response for version 1

**DOI:** 10.21956/wellcomeopenres.23505.r87000

**Published:** 2024-06-26

**Authors:** Mor Peleg

**Affiliations:** 1 University of Haifa, Haifa, Haifa District, Israel

A. *Is the work clearly and accurately presented and does it engage with the current literature?*

 I am not an expert on human behavior and thus I am not aware of specific ontologies covering this domain. However, I am aware of the top-level ontologies that the authors used (extended them to cover human behavior), such as Basic Formal Ontology and the Behavior Change Intervention Ontology, and they are highly appropriate.

 In terms of being accurate and clear – please see my Major comments because the descriptions should be more precise and consistent.

 B. *Is the study design appropriate and does the work have academic merit?*

 Yes. The 7-step process of developing the HBO is appropriate. In particular, the methodological search for sources (ontologies/classifications of human behaviors) was extensive.

 I appreciate very much the extensive work that went into reading all of the relevant literature of related ontologies and classifications, the identification of the many different classes related to human behaviors and their arrangement in a manageable taxonomy. I also agree with how the classes were classified into the Process hierarchy (occurrent) and Continuants. But there are some glitches that I noted in my Major Comments below.

 ***I found the 128 individual human behaviour classes organized into 9 superclasses to be highly appropriate in terms of covering different human behaviour that I have encountered in my research. I think that this is a very important contribution to the research community that will allow standard annotation of human behaviors and comparative analysis of studies.

 Regarding the evaluation in Step 5 and the refinement in Step 3: how the ontology could be used to annotate research reports -- I think that the examples (see my Major Comments per Table 7) should be more general and should relate to BCI Scenarios (per BCIO), which could be extracted from systematic reviews of BCIs.

 I appreciate very much that in step 4, 94 (!) behaviour change experts and four ontology experts were invited for the expert stakeholder review. You report that 10 experts (8 domain experts (in human behavior) and 2 ontology experts). Can you summarize (as the 65-page appendix in https://osf.io/r8g6a is very long to review) how thorough those reviews were (those of the ontology experts in particular)?

 As you note in Step 7, the ontology is a live document and there will be additional rounds of improvement. I think that at least one more round is needed (see my Major Comments), especially to improve the structure of the hierarchy and to arrive at a single consistent version that is shared. Representativeness and clarity are of good quality in my opinion.

 I was also hoping to have more axioms defined in the ontology as well as a specification of Domain and Range for relations. If your intention is just to provide a hierarchy of concepts, then this should be clarified in the paper. However, you specifically write: "Additional relations needed to be specified between classes,

 as a hierarchical classification system on its own would not be adequate to characterise the way that behaviours are described in the research literature."

 The inter-rater agreement in two-steps is appropriate, but you should consider two aspects: identifying the correct class in the hierarchy of HB, but also, related to Table 7, the proper relations that assemble these classes (concepts) into a BCI Scenario, which is a much more complex task, and the examples in Table 7 show that more work is needed to reach correct specification, meaning that (a) there should be more consistency in the ontology, and (b) more detailed examples provided as educational materials for users of the ontology.

 I commend you for basing your method for proposing an initial list of ‘behavioural attributes’ by drawing on the ‘Big Question': “What works, compared with what, for what behaviours, how well, for how long, with whom, in what setting, and why?”

 C. *Are sufficient details of methods and analysis provided to allow replication by others?*

 Yes.

 D. *If applicable, is the statistical analysis and its interpretation appropriate?*

 Not applicable

 E. *Are all the source data underlying the results available to ensure full reproducibility?*

 Yes but please note that there are many versions of the ontology – see my Major Comments.

 F. *Are the conclusions drawn adequately supported by the results?*

 I would revise the statement "The HBO is a coherent classification framework that characterises a wide range of human behaviours in the context of behaviour change interventions and beyond". I found some inconsistencies which I noted in my Major comments and also there was a problem with versions. I would remove the word "coherent" for now.

**MAJOR COMMENTS**

 1) An Individual Human Behavior is defined as a 'bodily process' that realizes some 'human life function'

 How does an Individual Human Behavior relate to a Behavior Change Intervention Scenario? The Scenario has an Outcome Behavior. Outcome Behavior is a Population Behavior (which is a Process Aggregate). A Process Aggregate is "An occurrent consisting exactly of a plurality of processes that are process_aggregate_member_parts_of that occurrent for all times at which it exists.", however I couldn't find the property "process_aggregate_member_parts_of" in the ontology. But if this property would be defined, and I understand correctly, then probably the connection would be made in the following way (I use "." to walk from an entity to its property and follow the property value and in '[ ]' I write the class type):

 Behaviour_Change_Intervention_Scenario.Outcome_Behaviour. process_aggregate_member_parts_of *contains* **([Process] Individual_Human_Behaviour or [Process] Individual_Human_Behaviour_Pattern)

 2) The ontology follows the minimal ontological commitment too much, that is, I think that the Domain and Range for properties should be added to prevent ontology developers and users from using the properties in a wrong way. At least the Range should be more constrained.

 3) There are very few axioms defined in the ontology. For example, the class "human_life_function" has only one axiom: is-a function, but nothing about this function being related to a 'human_being' class is specified formally.

 Moreover, the examples of usage for human_life_function are informative but are not specified formally in the ontology ("Learning function (e.g., knowledge development function and skill development function), knowledge or skill maintenance, protection of self, others and the environment, aesthetics function, bodily health function, spiritual function, social organisation function,, excretion function, nutrition function, reproductive function, respiration function,, rest function, travel function, thermoregulation function")"    >>while knowledge_development_behavior is a class in the ontology, knowledge_development_function is not. I'd like to note that I agree with the developers that 'knowledge_or_skill' class is-a 'expertise_of_person_source' and is not a function.

 All of the subclasses of individual_human_behaviour all have the single inherited axiom (bodily_process and realizes some human_life_function). So most of the knowledge is defined informally in textual definitions.

 I would like to see many more axioms, like you have defined for Behaviour_Change_Intervention_Scenario (8 axioms), which directs users of the ontology.

 I found a few defined classes. For example: 'human_communication_behaviour' is equivalent to: ('inter-personal behaviour' and 'expressive behaviour'). Also 'postural_expressive_behaviour' ('posture behaviour'  and 'expressive behaviour').

 4) The paper or the Zenodo and Github repositories should provide more direction on which owl file is the main one that we should open to view the ontology. For example, I am using Protégé 5.6 to view the ontology and I downloaded its parts: bcio, bcio_external, bcio_relations, bcio_upper_level, bcio_behaviour. Then I opened bcio.owl and I saw the axioms that I described in (1) above ('bodily process' that realizes some 'human life function') but I didn't see the definitions for 'human_communication_behaviour' and 'postural_expressive_behaviour', which I did see when I opened bcio_behaviour.owl (but then I didn't see the axioms as in (1)). Also, in bcio.owl I cannot see expressive_behavior (only creative_expressive_behavior and I cannot see postural_expressive_behaviour and 'human_communication_behaviour. I also cannot see these 3 classes in the BCIO Visualizer.

 ***Note also that in the paper (Table 3) you list 'creative expression behaviour' but in the bcio_behaviour.owl it is 'creative expressive behaviour'

 5) There was also a difference between the hierarchy of different subclasses of Individual_Human_Behaviour. For example 'Laughing' which in the bcio.owl is a direct subclass of expressive_behaviour, in the Visualizer it is a direct subclass of expressive_behaviour and of Vocalization and in bcio_behaviour it is a subclass of 'expressive_behaviour. The latter is agreement with Table 3 in the paper.

 When I view bcio_behaviour I see a hierarchy of 9 subclasses of individual_human_behavior , which is in agreement with Table 3. But when I view bcio.owl in Protégé I see 5 additional subclasses: habitual_behaviour, inter-personal_behaviour, laughing, normative_behaviour, and vocalization_behaviour and I don't see expressive_behaviour

 The same is true for Table 4, which is different than bcio_behaviour and different than bcio.owl and with the visualizer – so 4 different versions.

 Table 6: I couldn't find all of these relations in the Vizualizer, in the bcio and in the bcio_behaviour. For example, I found starts only in bcio.owl (but not in the Vizualizer). I couldn't find 'has abstinence period' anywhere (I also checked bcio_relations.owl). I didn't check all of the other relations.

 6) I found the BCIO Visualizer helpful but incomplete. For example, when I search for Behaviour Change Intervention Scenario I only see one class with no relations to other classes, which is different from what I see when I visualize in Protege and I see 8 axioms.

 7) Table 7:

 Row 1: the particular duration of "3" hours is not specified. Perhaps it could be specified with an individual of duration. Behavioural_duration (or duration in general) could have properties to specify the duration. There could be a minimum duration, a maximum duration for example. There are OWL ontologies for time that you could perhaps include.

 Row 2: has_behavioural_outcome: I couldn't find this relation in any version of the ontology. I think that it would be more useful to provide a more general example of a BCI Scenario that provides a recommendation for a BCI in which there is some kind of BCI that should result in some outcome behaviour. As we have written in our SATO paper ( https://www.sciencedirect.com/science/article/pii/S1532046422002817 - see Table 1), we think that a Scenario should have a clinical goal that could be achieved if there is a BCI that results in an Outcome Behaviour that could lead to the clinical goal. For example, I think that in this example, we could define a BCI Scenario that has a clinical goal of high happiness (disposition, as measured by some self-reported outcome that is higher than some threshold). Its Intervention_Outcome could be performing happiness-increasing interventions. One of the possible BCIs that could increase the happiness could be an intervention of walking with a friend. Its (intended) Outcome Behaviour could be Walking in the park 4 times a week (note that this should be a pattern). I think that connecting the human behaviour ontology to the previously developed BCIO is very important.

 Rows 2+3: I couldn't find relations of 'has behavioural goal' or 'has behavioural target' (BCIOR:000017) or 'has behavioural outcome'. I also couldn't find "complete remission' or "remission" in any version. Please use the correct relations and point to the classes that are the Range of those properties. Again, it would help if you could create and describe a particular BCI Scenario.

 8) "The classes ‘outcome behaviour’ and ‘goal-related behaviour’ were removed from the ontology as these constructs were judged to be better represented using the relations ‘has behavioural goal’ and ‘has behavioural outcome’ (see details in Step 6)."

 Step 6 does not provide enough details. Outcome_Behaviour is already specified in the BCIO. The example that you provide regarding happiness (p. 14) treats happiness as an emotion from the Emotion Ontology, yet in your ontology I see Happiness as a subclass of Emotion_Process, which is a subclass of Mental Process. So it is not an Individual Human Behaviour (which is correct, I agree). Please look at my comment #7, Row 2 regarding happiness as a disposition that can be measured and which could be the goal of a BCI Scenario (BCI_Scenario has_goal Happiness of >8 out of 10 on some Happiness Scale).

**MINOR COMMENTS:**

 1) The definition for Interoperability in Table 1 should refer to semantic consistency between the two integrated ontologies

 2) Logically-defined class in Table 1: it is possible to use the NOT operator. In addition, there are disjointness axioms that allow defining that certain classes are disjoint (there are no individuals that could be members of both classes).

 3) Ontology (Table 1): the definition is partial. An ontology is a representation of the types of entities in a given domain. It usually contains not just classes, but also their (datatype) properties or relationships (object properties).

 4) OWL definition in Table 1: perhaps add that it is a standard of the W3C, that it is a subset of RDF, and that it can be used to reason about ontologies: make inference based on ontological axioms, check for consistency.

 5) You provide an example of "Writing" as a human behavior. But Writing is not included in the ontology. You could add it to the ontology or you can switch the example to laughing.

 6) "‘urinating’ was added as an example of ‘excretion function behaviour’"

 Here, I would expect to find an Individual of 'urinating' which is a member of ‘excretion function behaviour’. Is my understanding correct? It would be good to explain this is terms or OWL terminology (eg, Individual, Class) and not just by providing annotations in text.

Is the work clearly and accurately presented and does it cite the current literature?

Partly

If applicable, is the statistical analysis and its interpretation appropriate?

Not applicable

Are all the source data underlying the results available to ensure full reproducibility?

Yes

Is the study design appropriate and is the work technically sound?

Yes

Are the conclusions drawn adequately supported by the results?

Partly

Are sufficient details of methods and analysis provided to allow replication by others?

Yes

Reviewer Expertise:

ontologies, clinical decision support, computer-interpretable guideline, digital behavior change interventions

I confirm that I have read this submission and believe that I have an appropriate level of expertise to confirm that it is of an acceptable scientific standard, however I have significant reservations, as outlined above.

## References

[1] : SATO (IDEAS expAnded wiTh BCIO): Workflow for designers of patient-centered mobile health behaviour change intervention applications. *J Biomed Inform* .2023;138: 10.1016/j.jbi.2022.104276 104276 10.1016/j.jbi.2022.104276 36586499 PMC9975785

# Reviewer report: Reviewer response for version 1

**DOI:** 10.21956/wellcomeopenres.23505.r84951

**Published:** 2024-06-10

**Authors:** Lauren Chan

**Affiliations:** 1 The University of Chicago, Chicago, Illinois, USA

Thank you for the opportunity to review this interesting manuscript from Schenk et al. about the development and evolution of the Human Behavior Ontology (HBO), a component of the Behaviour Change Intervention Ontology (BCIO). Within this paper, the authors provide a thorough description of their conceptualization and curation process for HBO.

 The manuscript is well outlined and highlights the unique landscape of behavior ontologies available at present and the opportunities to further refine standardized terminology for human behavior through the creation of HBO. A particular strength of this work was the stakeholder review process conducted including the use of educational videos to prepare the reviewers prior to them providing feedback and the inter-rater reliability evaluation of ontology annotations and subsequent revisions. 

 A few opportunities for potential clarification or addition include: 

1. A variety of examples of behavior terminologies are provided in Table 2, but there are some other resources related to behavior that may be of interest. These include the Human Phenotype Ontology (HPO) which contains some behavioral content, including that contained within the ‘Atypical behavior’ (HP:0000708)  branch. Additionally, projects such as Cognitive Atlas ( https://www.cognitiveatlas.org/) and The Cognitive Paradigm Ontology ( https://pubmed.ncbi.nlm.nih.gov/21643732/) have related content, although potentially a more specific scope than HBO. While I appreciate that HBO is focused on a more general approach to representing behavior as opposed to the more specific applications seen previously, cross references, mappings, and/or examples of harmonization between existing ontologies or terminologies with HBO would be helpful to improve future work utilizing HBO for data harmonization.
2. Given the promising work presented in this manuscript, further integration of HBO into the ontology landscape would be appreciated. On this note, while a specific browser for BCIO projects is good, hopefully HBO can be proposed for inclusion in other popular ontology browsing tools (e.g., OntoBee, OLS) so that community members using those tools can find and use this content should they not yet be aware of the BCIO browsing tools.
3. Further establishment and documentation of HBO on the BCIO GitHub pages would be helpful as I found it somewhat difficult to navigate to the appropriate OWL files for download and exploration of HBO. Additionally, greater information about how to become a contributor to HBO on the GitHub repository in a Read Me or other file is needed with information such as how to get an identifier range for term requests or guidelines for contributions. While they may be consistent with BCIO’s management strategy, information about how HBO will be maintained and versioned could be beneficial additions to this paper. In general, greater detail regarding long term plans for community involvement and maintenance of HBO would be helpful within the manuscript and on the GitHub repository.
4. Within the Strengths and Limitations section, there seems to be conflicting information regarding which GitHub issue tracker would be best used for HBO recommendations as two different repositories were mentioned. Clarification as to the most appropriate tracker for these requests is needed. 

One small potential typo that warrants correction is below:

- Step 7: first paragraph, line “A ROBOT template is a comma-separated values (CSV) file that can prepared using spreadsheet software…”, should say “can be prepared…”

Overall, I appreciate the work that has been put into HBO and the efforts to aggregate general behavior terminology. Small revisions as noted above would improve the reader experience and overall quality of the manuscript and HBO documentation, after which it is ready for approval. I look forward to applications of HBO in the future as well as greater integration of HBO into the broader biomedical ontology landscape.

Is the work clearly and accurately presented and does it cite the current literature?

Yes

If applicable, is the statistical analysis and its interpretation appropriate?

Yes

Are all the source data underlying the results available to ensure full reproducibility?

Yes

Is the study design appropriate and is the work technically sound?

Yes

Are the conclusions drawn adequately supported by the results?

Yes

Are sufficient details of methods and analysis provided to allow replication by others?

Partly

Reviewer Expertise:

nutrition, biomedical ontologies, data harmonization

I confirm that I have read this submission and believe that I have an appropriate level of expertise to confirm that it is of an acceptable scientific standard, however I have significant reservations, as outlined above.
